# Modelling viral encephalitis caused by herpes simplex virus 1 infection in cerebral organoids

**DOI:** 10.1038/s41564-023-01405-y

**Published:** 2023-06-22

**Authors:** Agnieszka Rybak-Wolf, Emanuel Wyler, Tancredi Massimo Pentimalli, Ivano Legnini, Anna Oliveras Martinez, Petar Glažar, Anna Loewa, Seung Joon Kim, Benedikt B. Kaufer, Andrew Woehler, Markus Landthaler, Nikolaus Rajewsky

**Affiliations:** 1 Organoid Platform, Berlin Institute for Medical Systems Biology (BIMSB), Max Delbrück Center for Molecular Medicine in the Helmholtz Association (MDC), Berlin, Germany; 2 Laboratory for RNA Biology, Berlin Institute for Medical Systems Biology (BIMSB), Max Delbrück Center for Molecular Medicine in the Helmholtz Association (MDC), Berlin, Germany; 3 Laboratory for Systems Biology of Gene Regulatory Elements, Berlin Institute for Medical Systems Biology (BIMSB), Max Delbrück Center for Molecular Medicine in the Helmholtz Association (MDC), Berlin, Germany; 4 Charité—Universitätsmedizin Berlin, corporate member of Freie Universität Berlin and Humboldt-Universität zu Berlin, Berlin School of Integrative Oncology (BSIO), Berlin, Germany; 5 System Biology Imaging Platform, Berlin Institute for Medical Systems Biology (BIMSB), Max Delbrück Center for Molecular Medicine in the Helmholtz Association (MDC), Berlin, Germany; 6 Institut für Virologie, Freie Universität Berlin, Berlin, Germany; 7 Institut für Biologie, Humboldt Universität zu Berlin, Berlin, Germany; 8 Charité—Universitätsmedizin, Berlin, Germany; 9 German Center for Cardiovascular Research (DZHK), Site Berlin, Berlin, Germany; 10 NeuroCure Cluster of Excellence, Berlin, Germany; 11 German Cancer Consortium (DKTK), Berlin, Germany; 12 National Center for Tumor Diseases (NCT), Site Berlin, Berlin, Germany; 13 Present Address: Centre for Genomics, Functional Genomics Programme, Human Technopole, Milan, Italy; 14 Present Address: Department of Infectious Diseases and Respiratory Medicine, Charité—Universitätsmedizin Berlin, corporate member of Freie Universität Berlin and Humboldt Universität zu Berlin, Berlin, Germany; 15 Present Address: Wellcome Sanger Institute, Wellcome Genome Campus, Hinxton, Cambridge, UK; 16 Present Address: Howard Hughes Medical Institute, Janelia Research Campus, Ashburn, VA USA

**Keywords:** Viral infection, Neuroscience

## Abstract

Herpes simplex encephalitis is a life-threatening disease of the central nervous system caused by herpes simplex viruses (HSVs). Following standard of care with antiviral acyclovir treatment, most patients still experience various neurological sequelae. Here we characterize HSV-1 infection of human brain organoids by combining single-cell RNA sequencing, electrophysiology and immunostaining. We observed strong perturbations of tissue integrity, neuronal function and cellular transcriptomes. Under acyclovir treatment viral replication was stopped, but did not prevent HSV-1-driven defects such as damage of neuronal processes and neuroepithelium. Unbiased analysis of pathways deregulated upon infection revealed tumour necrosis factor activation as a potential causal factor. Combination of anti-inflammatory drugs such as necrostatin-1 or bardoxolone methyl with antiviral treatment prevented the damages caused by infection, indicating that tuning the inflammatory response in acute infection may improve current therapeutic strategies.

## Abstract (web summary)

Modelling herpes simplex virus infection in brain organoids reveals that combined antiviral and anti-inflammatory treatment prevents neuronal damage caused by viral infection.

## Main

Herpes simplex virus type 1 (HSV-1) is a common pathogen affecting a large part of the human population worldwide^1^. Following primary replication in epithelial cells, HSV-1 establishes latency in the trigeminal ganglia and latent infections can persist over decades. However, stimuli such as stress signals and weakened immunity can cause the re-activation of HSV-1 from sensory neurons at any time^1^

Herpes simplex encephalitis (HSE), caused by re-activation of viral replication or by new lytic infection in the brain, is life threatening in absence of treatment^2,3^. HSV-1 is the most common cause of HSE, which accounts for 5–15% of infectious encephalitis in children and adults^4^.

Antiviral therapies strongly reduce the mortality rate of patients with HSE^3^. However, large proportions of recovered patients show moderate to severe neurological sequelae^2,3^. One possible explanation of brain injury is neuroinflammation^3^.

The current understanding of the mechanisms of HSV-1 infection, latency and re-activation in the central nervous system are based either on animal models or on in vitro differentiated neurons. These models cannot fully account for human host–pathogen interaction specificity or cellular and functional diversity in neural tissues, respectively^5,6^.

In recent years, human stem cell-derived brain organoids emerged as models that capture several important aspects of human brain development, tissue architecture and physiology^7–9^. Brain organoids have been used to study Zika virus neurotropism^10–13^, Japanese encephalitis virus^14^ and severe acute respiratory syndrome coronavirus 2 infection^15^. In the context of HSV-1, acute and latent-like infection states have been established in 3D neuronal culture, which demonstrated that HSV-1 can cause dramatic changes in neuronal morphology, processes and syncytium formation^5,16–18^. Furthermore, HSV-1 impairs identity of neuroepithelial and neuronal progenitor cells during early organoid development^16^. Given the complexity of these models, unbiased and high-throughput methods become necessary to get a complete picture of pathological changes and identify better therapeutic interventions.

In this Article, we applied single-cell RNA sequencing (scRNA-seq), neuronal activity measurements and imaging to capture the molecular and functional consequences of HSV-1 infection in human brain organoids. Real-time calcium imaging showed that HSV-1 infection massively decreases neuronal activity. An unbiased, exploratory gene set enrichment analysis (GSEA) identified tumour necrosis factor (TNF) signalling as the most highly and broadly induced pathway upon infection. Its activation was not stopped by antiviral acyclovir (ACV) treatment, although this significantly reduced viral load in all cell types. Only co-treatment with ACV and anti-inflammatory drugs such as necrostatin-1 (NEC-1) or bardoxolone methyl (CDDO-Me) reduced TNF pathway activation, prevented damages of neuronal processes, and preserved neuroepithelial integrity. Together, our data indicate that brain organoids serve as a powerful tool to study neurotropic/neuroinvasive viruses and to model future therapeutic strategies as exemplified here by identification of the synergistic effect displayed by a combinatorial antiviral/anti-inflammatory treatment.

## Results

### Cerebral organoids reflect complex 3D neuronal tissue

**Fig. 1:**
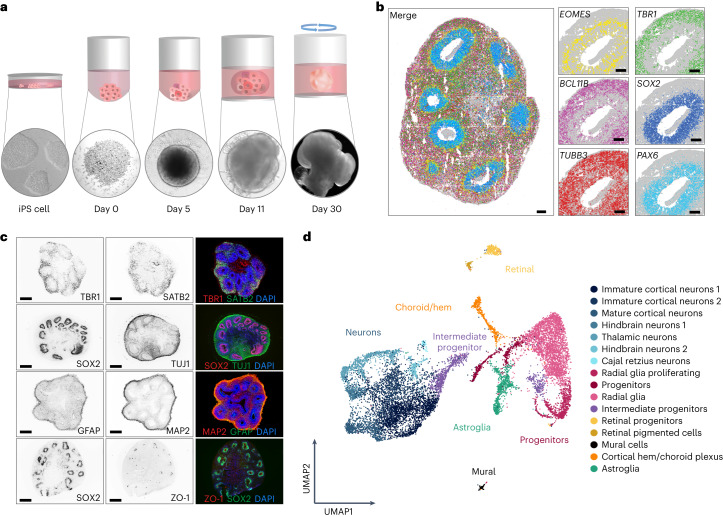
Cerebral organoids development at single-cell and spatial resolution. **a**, Schematic overview of cerebral organoids generation using iPS cells. Bottom: representative images of organoids at different developmental stages. **b**, Representative images from the Resolve Biosciences’ Molecular Cartography analysis of 60-day-old organoid from iPS cell line 1 for markers *EOMES* (intermediate progenitors), *SOX2/PAX2* (progenitors), *TBR1* (deeper-layer cortical neurons), *BCL11B* (upper-layer cortical neurons) and *TUBB3* (neurons). Scale bar, 100 µm. Representative image of one experiment with two biological replicates (*n* = 2). **c**, Exemplary immunohistochemistry of 60-day-old cerebral brain organoids showing expression of TBR1 (deeper-layer cortical neurons), SATB2 (upper-layer cortical neurons), SOX2 (progenitors), TUJ1/MAP2 (neurons), GFAP (astroglia) and ZO-1 (neuroepithelial junctions). Scale bar, 500 µm. Right: merged images co-stained with nuclear marker DAPI (blue). Representative image of three experiments from two independent iPS cell lines (*n* = 3 independent organoids/iPS cell line/experiment). **d**, UMAP plot of scRNA-seq data. Colours are mapped to the clusters (annotated on the right); broader cell-type definitions are overlayed on the plot. Data from two independent iPS cell lines, *n* = 2 biological replicates each.

**Extended Data Fig. 1:**
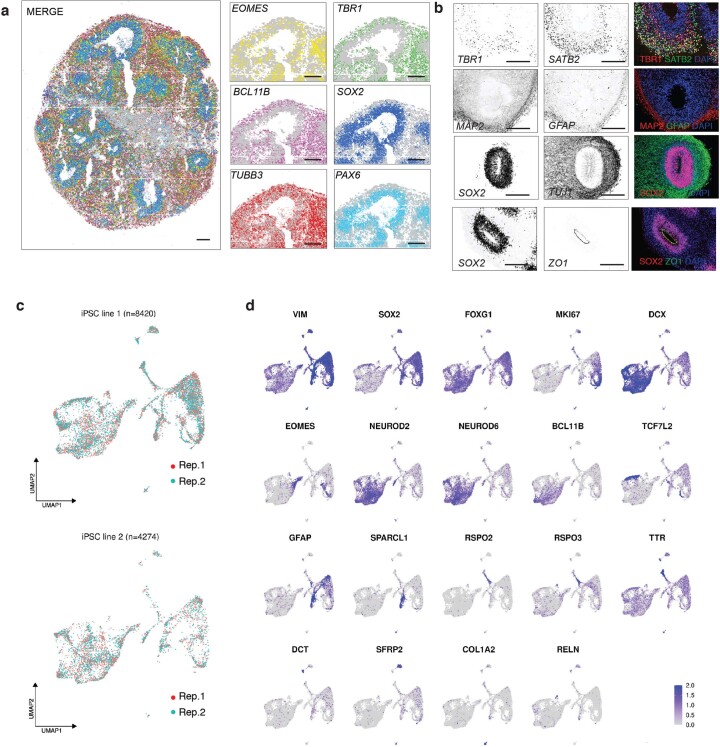
Cerebral organoids development at single cell and spatial resolution. **a**, Representative images from the Resolve Biosciences’ Molecular Cartography analysis of 60 days old organoid from iPSC line 2 for markers: *EOMES* – intermediate progenitors, *SOX2/PAX6* – progenitors, *TBR1 -* deeper layer cortical neurons*, BCL11B-* upper layer cortical neurons, *TUBB3*- neurons. Representative image of one experiment, n=2 biological replicates. Scale bar - 100 µm. **b**, High magnification of exemplary immunostaining of 60 days old cerebral brain organoids showing expression of TBR1 *-* deeper layer cortical neurons, SATB2*-* upper layer cortical neurons, SOX2- progenitors, TUJ1/MAP2 – neurons, GFAP- astroglia, ZO-1- neuroepithelial junctions. Representative of three independent experiments (n=3 independent organoids/ iPSC line / experiment). Scale bar - 100 µm. **c**, UMAP plots with cells colored by replicate (Rep) showing overlap between two biological replicates from control 60 days old cerebral organoids derived from induced Pluripotent Stem Cell (iPSC) line 1 (upper panel) and iPSC line 2 (lower panel). Scale bar - 100 µm **d**, UMAP plots depicting normalized expression of cell-type specific markers: *VIM, SOX2*-progenitors, *FOXG1*- telencephalic neurons and progenitors, *MKI67*-proliferating radial glia, *DCX*- neurons, *EOMES*-intermediate progenitors*, NEUROD2, NEUROD6* – cortical neurons, *BCL11B-* upper layer cortical neurons, *TCF7L2*- Thalamic neurons, *GFAP, SPARCL1*-astroglia, *RSPO2, RSPO3*- cortical hem, *TTR*-choroid plexus, *DCT*- retinal pigmented epithelium, *SFRP2*- retinal progenitors, COL1A2 – mural cells, RELN- Cajal Retzius neurons. All expression values are clipped to 2 for a better and homogenous color representation. (Two independent iPSC lines, each n=2 biological replicates).

We generated cerebral brain organoids from two genetically distinct induced pluripotent stem (iPS) cells lines, according to an optimized version of the Lancaster et al. protocol^7^ (Fig. 1a). These organoids had predominantly dorsal forebrain region specification and contained ventricle-like structures formed by SOX2^+^PAX6^+^ neural progenitors (apical radial glia), around ZO-1^+^ (tight junction) ring structures. With ongoing differentiation, EOMES^+^ intermediate progenitors and outer radial glia (oRG, HOPX, PEA15 and MOXD1) emerge radially therefrom. Finally, an early cortical-like structure appears at later timepoints, marked by MAP2^+^TUJ1^+^ neurons. At 60 days of differentiation, three main neuronal subtypes are observable, namely immature deep-layer projection neurons (*NEUROD2*, *NEUROD6* and *TBR1*), deep-layer corticofugal projection neurons (*BCL11B*), callosal projection neurons (*SATB2*) (Fig. 1b,c and Extended Data Fig. 1a,b), and GFAP astrocytes (Fig. 1c and Extended Data Fig. 1b,d). Our cerebral organoids thus contain the major cell types marking early human brain development^19^. Although organoids are not a direct equivalent of the adult temporal and frontal lobes, which are commonly affected in HSE, they represent the most complex human brain-like tissue system for studying viral infections of central nervous system in vitro^20^.

For an unbiased and comprehensive cell type characterization, we performed scRNA-seq analysis of 60-day-old organoids (Fig. 1d and Extended Data Fig. 1c,d). Combined highly reproducible single-cell transcriptomes from two biological replicates from two lines (shown in Extended Data Fig. 1c) showed 16 transcriptionally distinct clusters (Fig. 1d). These clusters were defined by cell-type-specific marker genes (examples in Extended Data Fig. 1d) in accordance with previous annotations^21,22^ and published atlases (Supplementary Fig. 1a^21^). Subsequently, we grouped them into progenitors, intermediate progenitors, neurons, astroglia, choroid plexus/cortical hem, retinal and mural cells (Fig. 1d).

### HSV-1 disrupts neuronal integrity and functionality

**Fig. 2:**
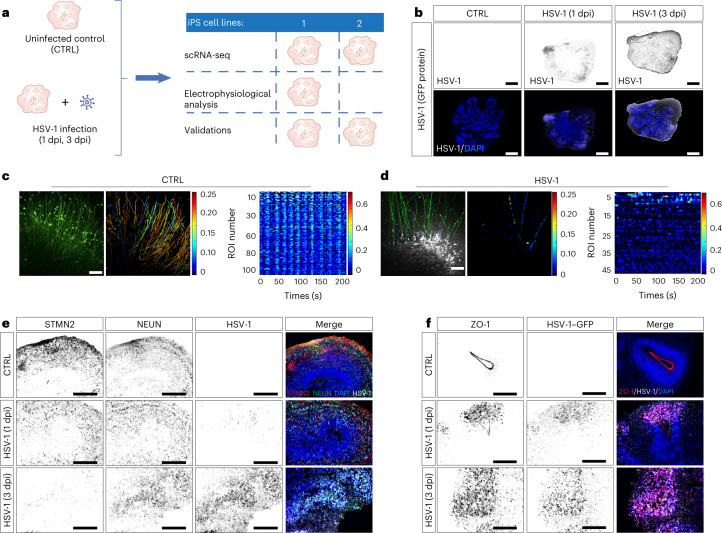
HSV-1 impairs synaptic activity and alters neuroepithelium identity. **a**, Schematic overview of experimental set-up. CTRL, uninfected control. **b**, HSV-1–GFP protein (black) expression in uninfected and 1 and 3 dpi HSV-1-infected 60-day-old organoids. Bottom: merged images showing HSV-1–GFP protein (white) and nuclear marker DAPI (blue). Scale bars, 500 µm. Images representative of three experiments. **c**, CalBryte590 AM-loaded (green) organoids (left); spike frequency illustrated by colour-coded image (middle); and spike detection plot for all regions of interest (ROIs) (right) in uninfected organoids. Scale bar, 100 μm. **d**, CalBryte590 AM-loaded (green) organoids showing HSV-1–GFP expression (grey) at 48 h post infection (hpi) (left); spike frequency illustrated by colour-coded image (middle); and spike detection plot for all ROIs (right) in 48 hpi HSV-1-infected (organoids. Scale bar, 100 μm. Images representative of one experiment, *n* = 3 biological replicates per condition. **e**, Immunohistochemistry for neuronal proteins STMN2 and NEUN, and HSV-1–GFP protein expression in uninfected and 1 and 3 dpi HSV-1-infected 60-day-old organoids. Right: merged images (HSV-1–GFP, white; STMN2, red, NEUN, green) co-stained with nuclear marker DAPI (blue). Scale bars, 100 µm. **f**, Immunohistochemistry for tight junction protein ZO-1 and HSV-1–GFP protein expression in uninfected and 1 and 3 dpi HSV-1-infected 60-day-old organoids. Right: merged images (HSV-1–GFP, white; ZO-1, red) co-stained with nuclear marker DAPI (blue). Scale bars, 100 µm. Images representative of three experiments, with two independent iPS cell lines (*n* = 3 independent organoids/condition/iPS cell line/experiment).

**Extended Data Fig. 2:**
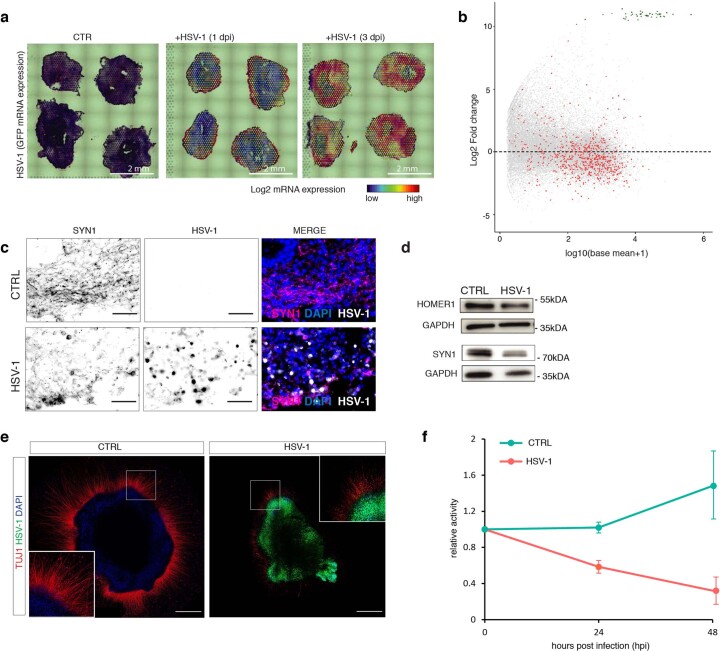
HSV-1 driven impairment of synaptic activity. **a**, Spatial transcriptomics analysis of HSV-1 GFP expression in uninfected (CTR) as well as 1 and 3 days post infection (dpi). Colors indicate log2 GFP mRNA expression. Representative image of one experiment, n=4 biological replicates. **b**, Fold changes (Log2FoldChange) of gene expression between control (CTR) and infected organoids 3 days post infection (+HSV-1 3dpi) versus log_10_ normalized mean transcript counts (baseMean). HSV-1 transcripts (green) and ‘CHEMICAL SYNAPTIC TRANSMISSION’ genes (red) are highlighted. Data from two independent iPSC lines, n=3 biological replicates/condition/iPSC line (see Methods). **c**, Exemplary immunohistochemistry for synaptic (SYN1 – black, magenta) neuronal, nuclei (DAPI – black, blue) and HSV-1 (GFP - white) in uninfected and infected organoids. Scale bar - 100 µm. Representative image of three independent experiments (n=3 independent replicates). **d**, Representative western blots of synaptic proteins (HOMER1, SYN1) and a housekeeping protein (GAPDH) of uninfected (CTRL) and infected (HSV-1) organoids. Representative image of three independent experiments, n=3 biological replicates. **e**, Mosaic image of the footprint of an uninfected organoid (CTRL) and HSV-1-GFP infected organoid (HSV-1) used in calcium imaging experiments showing TUJ1 expression (red), HSV-1-GFP expression (green) and DAPI (blue). Scale bar – 1 mm. Representative image of one experiment, n=3 independent organoids/condition were analyzed. **f**, Line chart showing the progression of the relative activity based on the mean spike frequency of uninfected (CTRL) and infected (+HSV-1) after 24 and 48 hours post-infection (hpi), n=3 independent replicates/condition, three measurements per timepoint. Error bars show the mean ± s.d. **g**, Exemplary images of HSV-1 GFP virus expression in sectioned infected and ACV treated organoids, immunostained with anti-GFP antibody. Infected (HSV-1 3dpi), ACV treated organoids (HSV-1 3dpi + ACV). Right panel: merged images, GFP (white), DAPI (blue). Scale bar - 500 µm. Representative image of three independent experiments (n=3 biological replicates). Source data

To balance between cellular complexity and increasing necrosis upon prolonged culturing^23^, we used 60-day-old organoids for all the infection experiments. We infected them with the green fluorescent protein (GFP)-expressing HSV-1 strain 17*syn*^+^ (ref. ^24^) and investigated molecular, cellular and physiological consequences of the infection (Fig. 2a). We analysed GFP mRNA expression using spatial transcriptomics data (10x Genomics Visium platform) and GFP protein using immunofluorescence and could show that the virus spreads into the organoid outer layers at 1 day post infection (dpi) and expands into deeper inner layers at 3 dpi (Fig. 2b and Extended Data Fig. 2a). Transcriptome changes of infected organoids were assessed using bulk and scRNA-seq at 1 and 3 dpi, and neuronal activity by calcium imaging after HSV-1 infection. Immunostainings and western blotting served as validation tools.

Previous reports showed that lytic HSV-1 neuronal infection caused synaptic dysfunction and disassembly of dendritic spines and upregulation of activity-regulated cytoskeleton-associated protein^25,26^. In our system, we observed massive downregulation of synaptic transmission genes (Extended Data Fig. 2b). Expression changes for synapsin-1 (*SYN1*) and postsynaptic density scaffolding protein 1 (*HOMER1*) were also confirmed at the p rotein level using immunostaining (Extended Data Fig. 2c) and western blot analysis (Extended Data Fig. 2d).

To analyse functional consequences of the infection, we measured intracellular calcium dynamics of spontaneous action potentials^27^. After plating the organoids, neurons (TUJ1^+^ cells) showed active migration and neurite extension towards the glass dish bottom (Extended Data Fig. 2e). Calcium imaging analysis indicated a high abundance of spontaneously active neurons in all organoids (day 0). Uninfected organoids had a high number of active neurites (Fig. 2c and Supplementary Video 1), which showed coordinated activity (Fig. 2c, right). In contrast, the robust initial spontaneous calcium activity decreased after 24 h upon infection and was almost completely abolished after 48 h (Fig. 2d, Extended Data Fig. 2f and Supplementary Video 2). The number of neurites was clearly reduced (Extended Data Fig. 2e), and the remaining ones were silent or showed limited and uncoordinated activity (Fig. 2d). Synaptic dysfunction is probably related to the strong downregulation of stathmin-2 (STMN2) a microtubule regulator important for axonal outgrowth and regeneration, and synaptic signalling^28,29^. Notably, STMN2 expression strongly declined after HSV-1 infection on protein level and RNA level (Fig. 2e and Supplementary Data Fig. 2a).

HSV-1 infection has been shown to impair neuroepithelial identity during the early stage of organoid development^16^. We tested for this by immunostaining of tight junction protein ZO-1, which is typically localized at the apical side of progenitors in neural rosettes^16,30^ (Figs. 1c and 2f). Notably, the ring structures of tight junctions were gradually lost with the progress of HSV-1-infection and ZO-1 protein was markedly overexpressed and mislocalized (Fig. 2f).

Lastly, we observed an induction of antisense transcription, as previously reported in primary fibroblasts^31^ (Supplementary Data Fig. 2b, two exemplary genes shown in Supplementary Data Fig. 2c).

### HSV-1 affects cellular composition in cerebral organoids

**Fig. 3:**
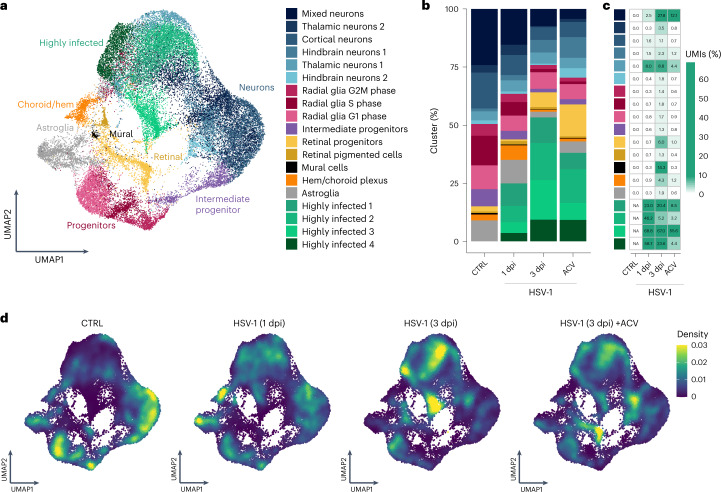
Cell-type-specific responses in HSV-1-infected and ACV-treated organoids. **a**, UMAP plot of cells from uninfected (CTRL), HSV-1-infected (1 and 3 dpi) and 3 dpi HSV-1-infected ACV-treated organoids. **b**, Proportion of cell types in uninfected, HSV-1-infected (1 and 3 dpi) and 3 dpi HSV-1-infected ACV-treated organoids. **c**, Mean viral load (percentage of viral UMI) in every cluster for each condition. Missing values (NA) are indicated for ‘highly infected’ clusters in uninfected organoids. **d**, UMAP plots of cells coloured by the density of cells from each condition in the specific UMAP area shown for uninfected, HSV-1-infected (1 and 3 dpi) and 3 dpi HSV-1-infected ACV-treated organoids, in which yellow and blue indicate increased and decreased density, respectively. Data from two independent iPS cell lines, *n* = 2 biological replicates/condition/iPS cell line. Source data

**Extended Data Fig. 3:**
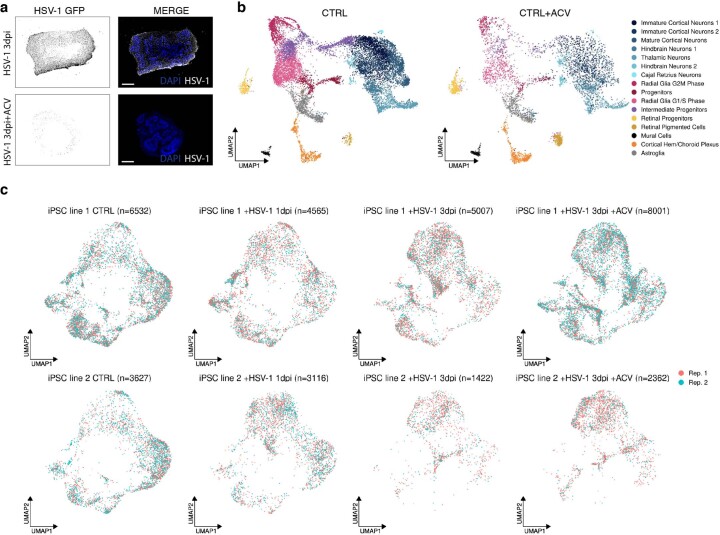
Acyclovir (ACV) treatment in control and HSV-1 infected organoids. **a**, Exemplary images of HSV-1 GFP virus expression in sectioned infected and ACV treated organoids, immunostained with anti-GFP antibody. Infected (HSV-1 3dpi), ACV treated organoids (HSV-1 3dpi + ACV). Right panel: merged images, GFP (white), DAPI (blue). Scale bar - 500 µm. Representative image of three independent experiments (n=3 independent replicates). **b**, UMAP plots of single cell RNA-seq data in control organoids (left UMAP) and ACV treated control organoids (right UMAP). Colors are mapped to the clusters (annotated on the right). **c**, UMAP plots of cells colored by replicate (Rep.) from uninfected organoids (CTRL), 1 day and 3 days post infection (HSV-1 1,3 dpi), and acyclovir treated, 3 days post infection (dpi) organoids derived from induced Pluripotent Stem Cell (iPSC) line 1 (left) and iPSC line 2 (right panel), n=2 independent replicates/condition/iPSC line.

To dissect cell-type-specific effects of HSV-1 infection in cerebral organoids, we performed scRNA-seq of 60-day-old organoids at early (1 dpi) and late (3 dpi) stages of infection (Fig. 3a). Current therapeutic strategies for HSE rely on the administration of viral replication inhibitors, such as ACV^32^. Accordingly, we also analysed organoids at 3 dpi, which were treated with ACV starting at 6–8 h after infection. Immunostaining showed efficient reduction of GFP protein expression throughout the entire organoid tissue by this treatment (Extended Data Fig. 3a). In addition, we performed scRNA-seq analysis of ACV-treated organoids in the absence of viral infection to control for non-specific effects of the treatment alone, and found no evidence of substantial effects on cellular transcriptomes (Extended Data Fig. 3b).

After quality filtering, we collected 36,392 single-cell transcriptomes across 16 samples from two independent cell lines (control, 1 dpi, 3 dpi, and 3 dpi ACV-treated, two replicates/organoids each), capturing host and viral polyadenylated transcripts. Numbers of recovered cells and cluster composition across replicates and cell lines were overall similar (Extended Data Fig. 3c,d).

To study cell-type-specific effects of viral infection, we hypothesized that cell types as defined at the transcriptomic level would not be changed upon infection. We therefore excluded viral transcripts for cell type labelling and performed data integration, low-dimensional embedding and clustering of control and infected samples (Fig. 3a,b). This analysis yielded 19 clusters, which we annotated on the basis of data from uninfected organoids (Methods). Notably, numerous (13.9–67.5%) cells from the infected samples formed four infection-specific clusters (Fig. 3a,b). The control cells detected in these clusters (1,760 cells representing 15.9% of these clusters and 14.8% of the control cells), exhibited about half the transcript counts (737 median unique molecular identifiers (UMIs) compared with 1,184 for the other control cells and 1,472 for cells from other timepoints in the same clusters) and were therefore excluded from downstream analyses.

**Extended Data Fig. 4:**
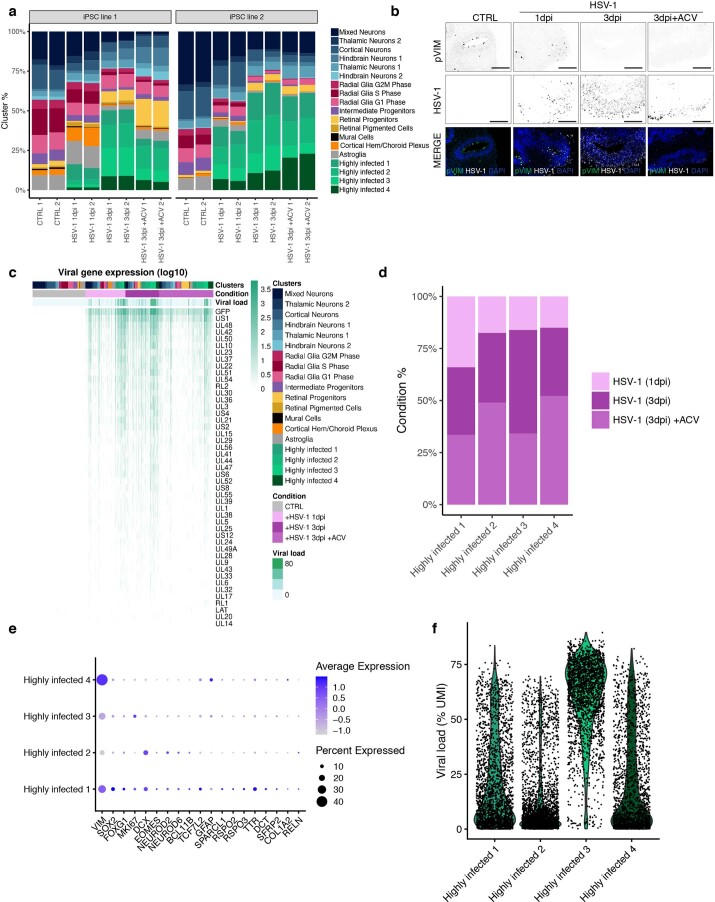
Cell-type specific responses in HSV-1 infected and Acyclovir (ACV) treated organoids. **a**, Proportion of cell types in uninfected, infected(1, 3dpi) and ACV treated organoids derived from iPSC line 1 (upper) and iPSC line 2 (lower panel). **b**, Representative immunohistochemistry for mitotic marker (pVIM – white) and merged images for mitotic marker (pVIM - green), HSV-1 (GFP - white), nuclei (DAPI - blue) in uninfected (CTRL), 3 dpi infected (HSV-1) and ACV treated organoids. Scale bar - 100 µm. Representative image of three experiment (n=3 biological replicates). **c**, Viral gene expression heatmap showing log10 expression values of detected viral genes. Genes (rows) are ordered by decreasing overall expression and cells (columns) are ordered and both by condition and cluster assignment. Cells are coloured by Condition, Clusters and Viral load. Data from two independent iPSC lines, each with n=2 biological replicates. **d**, Barplot showing the proportion of cells from each infected condition assigned to each Highly infected cluster. **e**, Dotplot showing the expression of selected marker genes in each Highly infected cluster. Color indicates average, scaled expression values and dot size shows the percentage of expressing cells in each Highly infected cluster. **f**, Violin plot showing the viral load (% of viral transcripts, that is Unique Molecular Identifiers or UMI) in each Highly infected cluster. Each dot represents a cell. (All data derived from two independent iPSC lines, each n=2, biological replicates).

In line with previous studies^16,33^, a prominent decrease in the number of proliferating cells (radial glia clusters) was observed (Fig. 3b and Extended Data Fig. 4a), which we also validated by immunohistochemistry using cell mitosis marker phospho-vimentin (Extended Data Fig. 4b).

The infection-specific cell clusters exhibited both extremely high viral loads (that is, the percentage of captured viral transcripts in each cell) and expression of numerous viral genes (Fig. 3c and Extended Data Fig. 4c). The loss of cellular identity (that is, the lack of co-clustering with the cell types in the control organoids) is therefore probably due to both high amounts of viral transcripts and perturbation of the host cell transcriptome. For simplicity, we annotated these cells as ‘highly infected’ (Fig. 3a–d). Highly infected cluster 1 was equally composed of cells from each condition, while highly infected clusters 2, 3 and 4 were mainly composed of cells from the HSV-1 3 dpi and 3 dpi ACV conditions (Extended Data Fig. 4d). In terms of canonical marker gene expression, highly infected cluster 2 expressed neuronal genes (for example, *DCX*, *NEUROD2* and *BCL11B*) and highly infected cluster 4 expressed progenitor genes (for example, *VIM* and *GFAP*), while highly infected clusters 1 and 3 did not exhibit clear expression signatures and probably represent a mixture of infected cells (Extended Data Fig. 4e). Of note, cells in highly infected cluster 3 showed an extremely high viral load (on average, almost 75% of recovered RNA; Extended Data Fig. 4f). Interestingly, despite considerably lower amounts of viral RNA in the ACV-treated organoids, the amount of highly infected cells was not markedly reduced. This could indicate that the strong perturbation of the cellular transcriptomes is a consequence of the infection itself and less of viral replication.

In summary, we observed a drastic change in the organoid cellular composition, which may be partially attributed to infected cells becoming no longer recognizable from scRNA-seq data alone. With the appearance of infection-specific clusters, the fraction of almost all cell types was reduced (for example, proliferating radial glia from 17.8% to 3.1%, Fig. 3b). Notably, highly infected cells already appear at 1 dpi, increase at 3 dpi reaching 53.3% and only partially decrease after 2 days of ACV treatment (Fig. 3b,c).

### TNF signalling activated by HSV-1 persists antiviral treatment

**Fig. 4:**
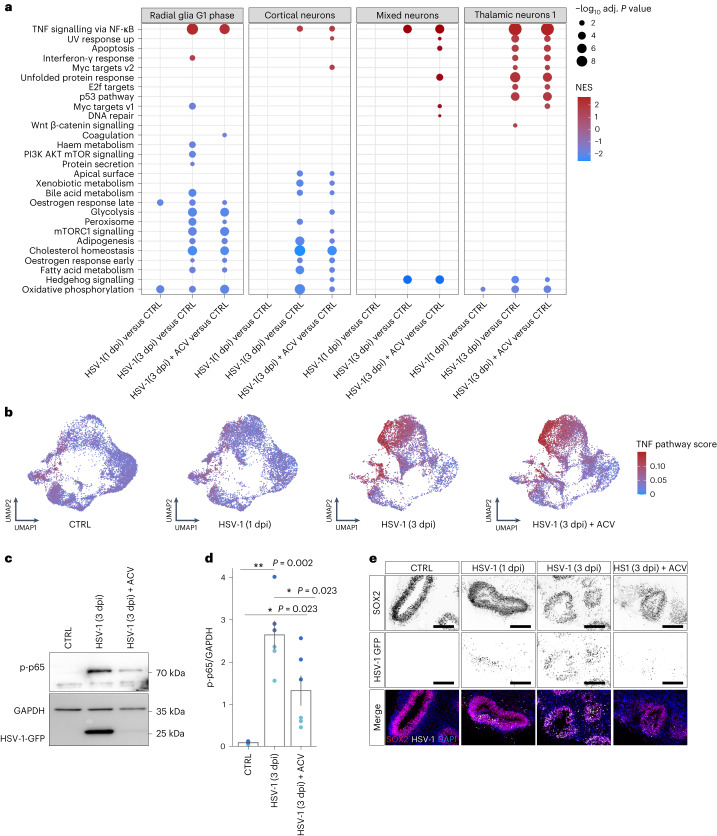
ACV does not prevent HSV-1-driven inflammatory responses. **a**, Enriched gene sets for the assessed cell types based on the comparison of HSV-1-infected (1 and 3 dpi) and 3 dpi HSV-1-infected ACV-treated organoids with uninfected organoids. Colour indicates normalized enrichment score (NES) values and dot size represents −log_10_-adjusted *P* value for statistically significant pathways according to gene set enrichment analysis (GSEA) analysis. **b**, UMAP plots of cells coloured by the TNF pathway activity score in each cell and separated in uninfected (CTRL), HSV-1-infected (1 and 3 dpi) and 3 dpi HSV-1-infected ACV-treated organoids, with red and blue indicating increased and decreased scores, respectively. Pooled data from two independent iPS cell lines, *n* = 2 biological replicates for each line. **c**, Representative western blot analysis of p-p65 expression in uninfected, 3 dpi HSV-1-infected and 3 dpi HSV-1-infected ACV-treated organoids; GAPDH serves as loading control, and GFP indicates virus expression (bottom). **d**, Semi-quantification of p-p65 protein expression by densitometry in uninfected, 3 dpi HSV-1-infected and 3 dpi HSV-1-infected ACV-treated organoids, normalized to GAPDH expression. *P < 0.05, **P < 0.01, Benjamini–Hochberg-corrected two-sided *t*-tests. *n* = 6 biologically independent samples; that is, six batches of pooled organoids from two iPS cell lines; colour indicates cell line of origin. Bar plots and error bars show mean ± standard error. **e**, Representative immunohistochemistry for SOX2 (top) and HSV-1–GFP protein expression (middle) in uninfected, HSV-1-infected (1 and 3 dpi), and 3 dpi HSV-1-infected ACV-treated organoids. Bottom: merged images (HSV-1–GFP, white; SOX2, red) co-stained with nuclear marker DAPI (blue). Scale bar, 100 µm. Representative image of three experiments (*n* = 3 independent organoids/condition/iPS cell line). Source data

**Extended Data Fig. 5:**
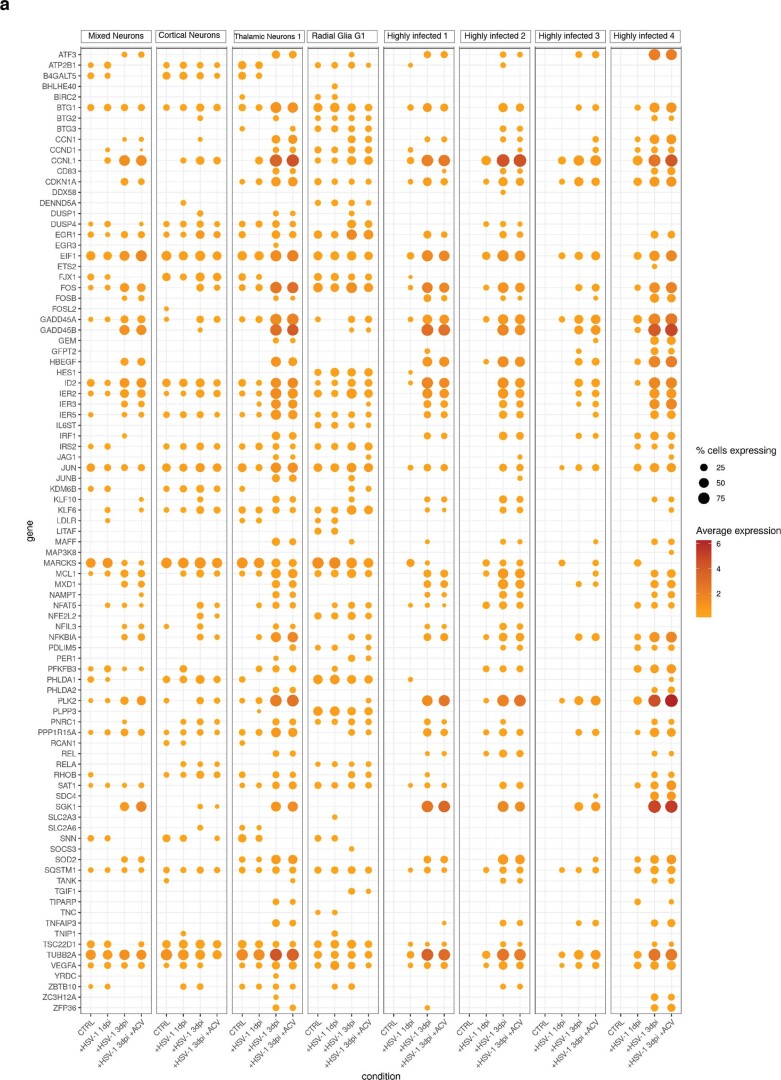
Viral infection induces inflammatory responses. **a**, Dotplot showing the expression of detected genes from the ‘TNF-α signaling via NF-κB’ HALLMARK gene set. Color indicates average, SCT-normalized expression values and dot size shows the percentage of expressing cells in each cluster. Only highly infected clusters and clusters included in the differential gene expression and gene set enrichment analysis are shown. Data pooled from two independent iPSC lines, each n=2, biological replicates.

**Extended Data Fig. 6:**
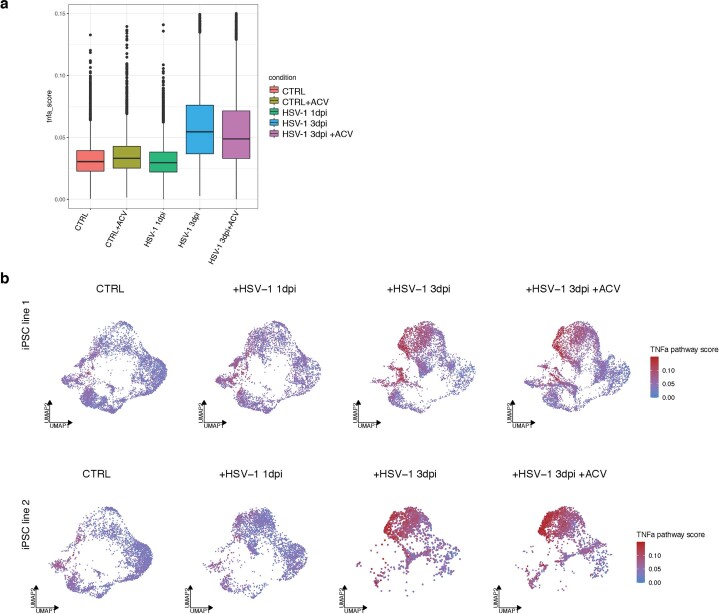
Acyclovir (ACV) treatment does not prevent inflammatory responses. **a**, TNF-α pathway activity score in each condition: uninfected (CTRL), uninfected ACV treated (CTRL +ACV), HSV-1-infected (HSV-1 1dpi, HSV-1 3dpi), and 3 dpi HSV-1-infected ACV-treated (HSV-1 3dpi +ACV) organoids. Boxplot centers represent median values, while the boxplot bounds represent the 25% and 75% quantiles. Boxplot whiskers represent the 25% quantile −1.5× interquartile range (IQR) and the 75% quantile +1.5× IQR, respectively. Data from two independent iPSC lines, n=2, biological replicates/condition/iPSC line. **b**, UMAP plots of cells colored by the TNF pathway activity score in each cell and separated in uninfected organoids, 1 day and 3 days post infection (dpi), and acyclovir treated, 3 days post infection (dpi) organoids, with red and blue indicating increased and decreased score, respectively. Data from two independent iPSC lines, n=2, biological replicates/condition/iPSC line.

To identify cell-type-specific molecular alterations induced by HSV-1 infection, we performed differential gene expression analysis comparing cells from each infected condition (1 dpi, 3 dpi, and 3 dpi ACV-treated) to uninfected cells in the same cluster (Methods). To increase the robustness of our analysis^34^, we aggregated randomly selected 50 cells per organoid sample and cluster (‘pseudobulk’) and focused on 4 cell types (‘mixed neurons’, ‘cortical neurons’, ‘thalamic neurons 1’, ‘radial glia G1 phase’) with at least 50 cells across most organoids (Methods; at least 80% or 13 out of 16 samples). Then, we performed GSEA leveraging the Hallmark gene sets (Methods). Notably, the term ‘TNF signalling via NF-κB’ was the only pathway being consistently upregulated in all analysed clusters. Interestingly, significant upregulation was detected only at 3 dpi and was not stopped by ACV treatment (Fig. 4a and Extended Data Fig. 5a). No effect was observed on the TNF signalling pathway in ACV-treated uninfected organoids (Extended Data Fig. 6a).

To extend our analysis to all clusters, we ranked genes by their relative expression in every cell and evaluated the enrichment of genes in the ‘TNF signalling via NF-κB’ gene set (Methods). Of note, TNF pathway activation was particularly strong in, but not restricted to, highly infected cells (Fig. 4b and Extended Data Fig. 6b).

The TNF cytokine is a key mediator of inflammation. The TNF and receptor superfamilies (TNFSF and TNFRSF) induce an intricate network of cellular signalling pathways, including activation of nuclear factor-kappa B (NF-κB) and its target genes^35^.

**Extended Data Fig. 7:**
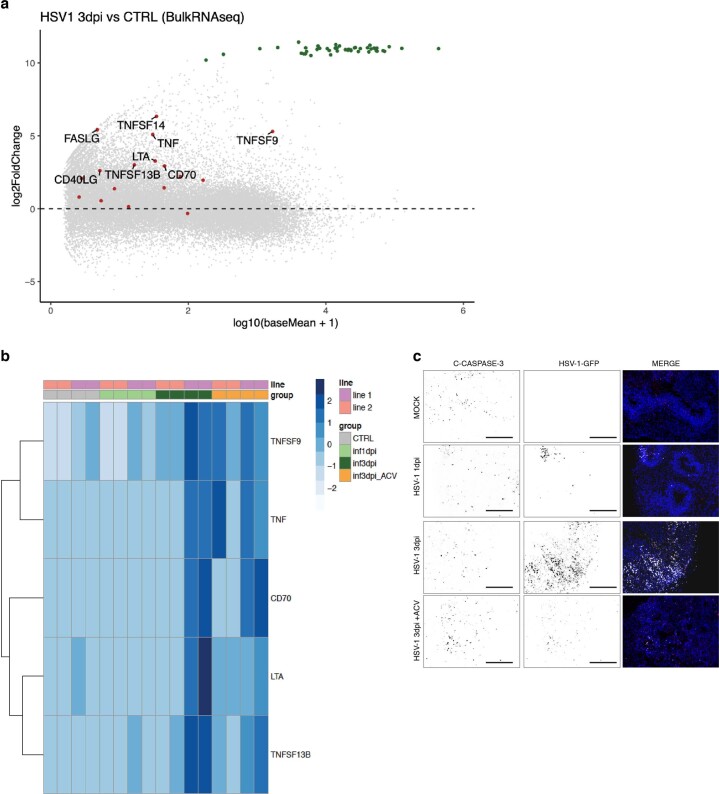
TNF ligands are upregulated upon infection. **a**, Fold changes (Log2FoldChange) of gene expression between control (CTR) and infected organoids 3 days post infection (+HSV-1 3dpi) versus log_10_ normalized mean transcript counts (baseMean). HSV-1 transcripts (green) and TNF ligands (red) are highlighted. Significantly differentially expressed TNF ligands with a log2 fold change > 2.5 are labelled. Transcript counts were measured from two independent iPSC lines, n=3 biological replicates/condition/iPSC line (see Methods). **b**, TNF ligands induced upon infection are not downregulated by acyclovir treatment. Row-mean normalized expression of upregulated TNF ligands in scRNA-seq data (pseudo-bulk). Transcript counts are shown for 2 replicates per condition (group) and two cell lines (line). **c**, Exemplary images of Cleaved Caspase 3 and HSV-1 GFP virus expression in sectioned and immunostained organoids, uninfected (Mock) infected with HSV-1 (HSV-1 1dpi, HSV-1 3dpi) and ACV treated (HSV-1 3dpi+ACV). Right panel: merged images, Cleaved Caspase 3(red), GFP (white), DAPI (blue). Scale bar - 100 µm, n=3 biological replicates/condition.

We quantified the expression of all TNF(R)SF members in bulk RNA-seq data from 3-day-infected organoids. *TNFSF9* and *TNF* were highly induced upon infection (Extended Data Fig. 7a). Using scRNA seq data, we then confirmed that most of the detected ligands are upregulated upon infection both with and without ACV treatment (Extended Data Fig. 7b).

We next examined whether HSV-1 infection leads to canonical NF-κB–RelA pathway activation, detectable by Ser536 phosphorylation in p65 (also known as RelA) in western blots. As expected, HSV-1 infection led to an increase of phosphorylated p65 (p-p65), which was only partially reduced with ACV treatment (Fig. 4c,d). Concomitantly, the impaired tissue integrity at 3 dpi HSV-1 infection was not stopped by ACV treatment, despite considerably lower amounts of GFP originating from the virus (Fig. 4e). We did not observe any increase in cleaved caspase-3 expression in infected organoids and treated organoids (3 dpi), and therefore excluded apoptosis as the cause of observed tissue damage (Extended Data Fig. 6c).

Furthermore, we specifically looked at the expression of NF-κB target genes, that is, having a NF-κB responsive site in the promoter or whose expression has been shown to be associated with NF-κB (ref. ^36^). While some genes were either constitutively expressed or mildly responding to the infection (for example, *HSP90AA1* and *YY1*), many were broadly activated upon infection and/or enriched in the ‘highly infected clusters’ (for example, *GADD45B*, *BCL2L11*, *NFΚBIA*, *CDKN1A* and *BNIP3*), whereas others were subject to more cell-type-specific changes (for example, *CD83*, *CDKN1A*, *PLK3* and *E2F3*) (Supplementary Data Fig. 3a).

These results indicate that antiviral treatment, although very effective when it comes to inhibition of viral replication, cannot stop the global inflammatory processes triggered by HSV-1 and possibly mediated by TNFSF members.

### Combinatorial treatment reduces defects in organoids

**Fig. 5:**
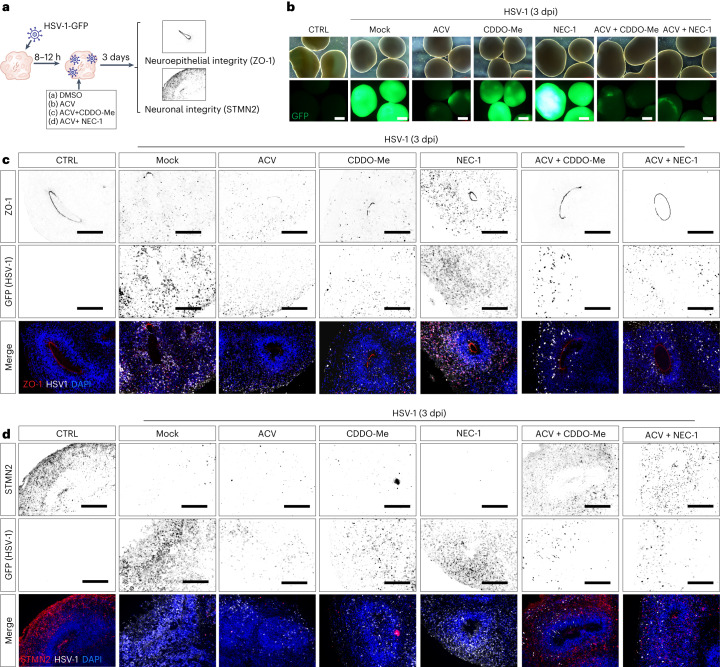
Combinatorial treatment strategies for HSV-1-driven neuroinflammation. **a**, Schematic overview of combinatorial treatment set-up. **b**, Exemplary images of intact organoids (top) and HSV-1–GFP protein expression (bottom) in uninfected (CTRL) and 3 dpi HSV-1-infected mock-treated (DMSO), ACV-treated, CDDO-Me-treated, NEC-1-treated, ACV and CDDO-Me-co-treated, and ACV and NEC-1-co-treated organoids. Scale bars, 800 µm. Representative image of three experiments from two different iPS cell lines (*n* = 3 independent organoids/condition/iPS cell line). **c**, Representative immunohistochemistry for ZO-1 (top) and HSV-1–GFP (middle) protein expression in uninfected and 3 dpi HSV-1-infected mock-treated, ACV-treated, CDDO-Me-treated, NEC-1-treated, ACV and CDDO-Me-co-treated, and ACV and NEC-1-co-treated organoids. Bottom: merged images (HSV-1–GFP, white; ZO-1, red) co-stained with nuclear marker DAPI (blue). Scale bars, 100 µm. Representative image of three experiments, two different iPS cell lines (*n* = 3 independent organoids/condition/iPS cell line). **d**, Representative immunohistochemistry for STMN2 (top) and HSV-1–GFP (middle) protein expression in uninfected and 3 dpi HSV-1-infected mock-treated, ACV-treated, CDDO-Me-treated, NEC-1-treated, ACV and CDDO-Me-co-treated, and ACV and NEC-1-co-treated organoids. Bottom: merged images (HSV-1–GFP, white; STMN2, red) co-stained with nuclear marker DAPI (blue). Scale bars, 100 µm. Representative image of three experiments, two different iPS cell lines (*n* = 3 independent organoids/condition/iPS cell line).

**Extended Data Fig. 8:**
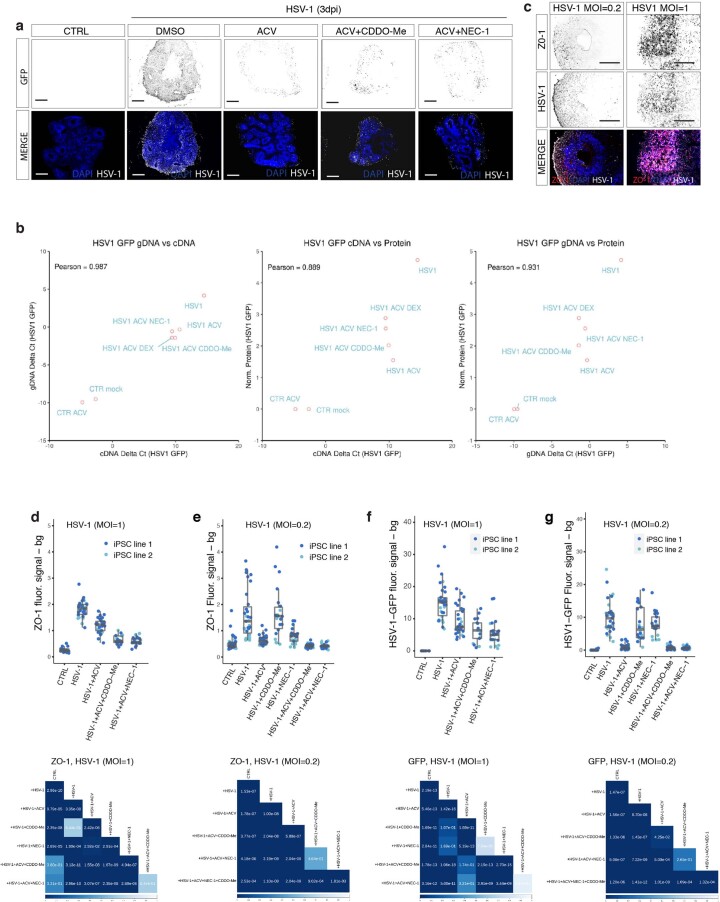
Combinatorial treatment strategies for HSV-1- driven neuroinflammation. **a**, Exemplary images of HSV-1 GFP virus expression in sectioned and anti-GFP antibody immunostained organoids. Uninfected (CTRL), infected (HSV-1), ACV treated organoids (ACV), ACV and CDDO-Me co-treated (ACV+CDDO-Me) and ACV and NEC-1 cotreated (ACV+NEC-1). Lower panel: merged images, GFP (white), DAPI (blue). Scale bar - 500 µm. Representative image of three independent experiments with two iPSC lines (n=3 biological replicates/condition/iPSC line). **b**, Scatterplots showing all comparisons between viral GFP RNA, measured as Delta Ct with respect to the human DICER1 gene in cDNA from whole organoids lysate, viral GFP gDNA, measured as before in genomic DNA from the same organoids, and viral GP protein, measured from Western blot densitometric analysis, normalized on GAPDH. All correlation coefficients (Pearson) are shown in the top left of each plot. Sample identity is indicated next to each dot, representing the mean between three biological replicates (n=3, biological replicates). **c**, Exemplary images of ZO-1 and HSV-1 GFP virus expression in sectioned and immunostained organoids, infected with high (MOI=1) and low (MOI=0,2) multiplicity of infection (MOI) of HSV-1. Lower panel: merged images, ZO-1(red), GFP (white), DAPI (blue). Scale bar - 100 µm. Representative image of three independent experiments with two iPSC lines. **d, e**, ZO-1 fluorescent signal quantification in sectioned and immunostained organoids infected with high (d) or low (e) multiplicity of infection (MOI) of HSV-1, each dot represents one neuroepithelial loop, colors indicated cell lines: iPSC line 1 (dark blue), iPSC line 2 (bright blue). Lower panel, p-values for the fluorescent signal quantification **f, g** HSV-1-GFP signal quantification in immunostained organoids infected with high (f) or low (g) multiplicity of infection (MOI) of HSV-1 virus, each dot represents one neuroepithelial loop, colors indicated cell lines. Lower panel: p-values (Benjamini-Hochberg-corrected pairwise Wilcoxon Mann Whitney test for the fluorescent signal quantification, n=14,34,32,17,23 individual neuroepithelial loops quantified from 3,5,6,4,4 individual organoids per condition for panels d and f, n=43,31,33,22,21,38,29 loops from 5,3,3,2,3,3,3 organoids for panels e and g, from two independent hiPSC lines and two MOIs. Boxplot show lower quartile, median and upper quartile, whiskers represent upper and lower quartile ± the smallest value within 1.5-fold of the interquartile range respectively. Source data

**Extended Data Fig. 9:**
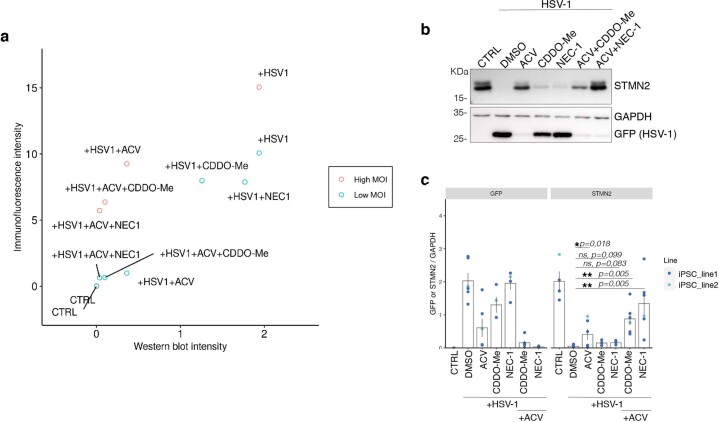
Combinatorial treatment reduces HSV-1- driven neuroinflammation. **a**, Scatterplot showing the comparisons between viral GFP protein measured from Western blot densitometric analysis (normalized on GAPDH) or immunofluorescence (signal intensity minus background, see methods), in green for ‘low MOI’ infections and red for ‘high MOI’ infections. Western blot was performed on ‘low MOI’ only. Sample identity is indicated next to each dot, representing the mean between all biological replicates from Supplementary Fig. 5C–F (IF) and Fig. 6B (WB). **b**, Representative western blots analysis of STMN2 expression in uninfected (CTRL), infected (HSV-1), ACV treated organoids (HSV-1+ACV), CDDO-Me treated (HSV-1+ CDDO-Me), NEC-1 treated (HSV-1+ NEC-1), ACV and CDDO-Me co-treated (HSV-1+ACV+CDDO-Me); and ACV and NEC-1 cotreated (HSV-1+ACV+NEC-1). GAPDH serves as loading control and GFP indicates virus expression (lower panel). Number of replicates is shown in panel c. **c**, Semi-quantification of STMN2 (right panel) and GFP (left panel) protein expression by densitometry in uninfected (CTRL), infected (HSV-1), ACV treated organoids (+ACV), ACV and CDDO-Me cotreated (+ACV+CDDO-Me) and ACV and NEC-1 cotreated (+ACV+NEC-1). P-values are indicated for the relevant comparisons, according to Benjamini-Hochberg-corrected two-sided t-tests (n=4 biologically independent samples for CTRL, HSV1+CDDO-Me and HSV1+NEC-1, n=6 for all others, that is 4-6 batches of pooled organoids; color indicates cell line of origin).Bar plots and error bars shown ± s.e. Source data

To test whether anti-inflammatory drugs can prevent the observed tissue and cellular damage observed upon infection, we used two different compounds targeting the TNF pathway, alone and in combination with ACV: first, necrostatin-1 (NEC-1), an RIP1-targeted inhibitor of TNF-induced necroptosis, and second, CDDO-Me, an NRF2 agonist that attenuates the NF-κB-mediated inflammatory response^37^. We infected 60-day-old organoids with low (0.2) and high (1.0) multiplicity of infection (MOI) of HSV-1 GFP virus and treated them 8–12 h after infection with different combinations of ACV, NEC-1 and CDDO-Me. As a readout, we analysed neuroepithelial integrity, neuronal damage and viral load by measuring ZO-1, STMN2 and GFP, respectively, using immunostaining and protein quantification (Fig. 5a and Extended Data Fig. 8a–c). In organoids treated with ACV alone or co-treated with anti-inflammatory agents, viral GFP expression was similarly reduced (Fig. 5b–d). Next, we assessed the localization of ZO-1 in infected organoids. Notably, immunohistochemistry analysis of ZO-1 expression revealed that ACV alone only partially rescued the architecture of organoid neuroepithelium, but co-treatment with both CDDO-Me or NEC-1 significantly improved neuroepithelium architecture and prevented ZO-1 hyperactivation in both iPS cell lines (Fig. 5c and Extended Data Fig. 8d–g). In addition, the downregulation of STMN2 expression, which was one of the strongest observed responses to HSV-1 infection in our system (Fig. 2e and Supplementary Fig. 2a), was significantly reduced in co-treated organoids, as shown by immunostaining and western blot analysis (Fig. 5d and Extended Data Fig. 9b,c). With a lower MOI, the phenotype was milder, and ACV alone showed stronger effect, although still superseded by co-treatments (Extended Data Figs. 8d–g and 9a). Treatment with CDDO-Me or NEC-1 alone did not prevent defects triggered by HSV-1, indicating that effectively restoring tissue architecture requires blocking both viral replication and inhibiting TNF signalling (Fig. 5c,d and Extended Data Fig. 9b,c).

**Fig. 6:**
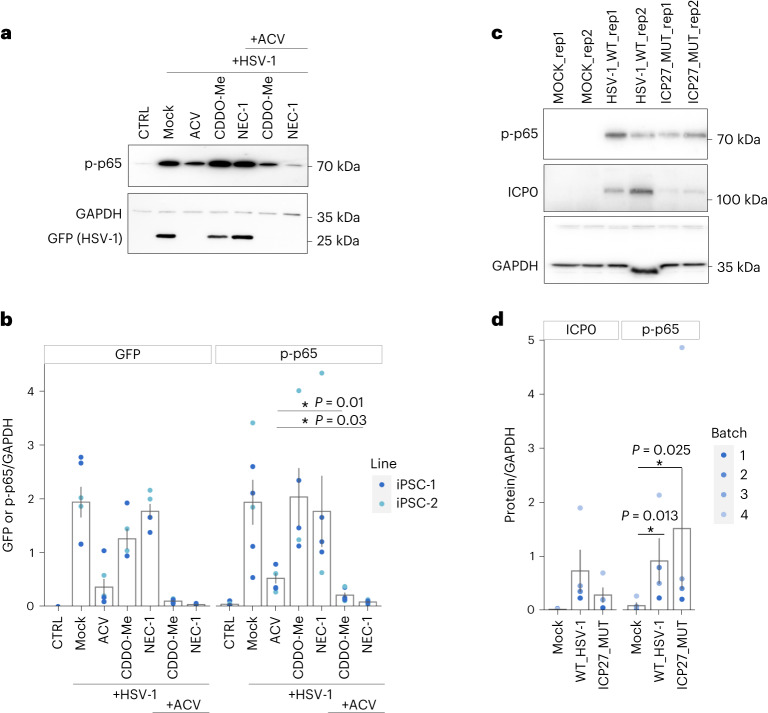
Combinatorial treatment reduces HSV-1-driven neuroinflammation in HSV-1-infected organoids. **a**, Representative western blots analysis of p-p65 expression in uninfected (CTRL) and 3 dpi HSV-1-infected mock-treated (DMSO), ACV-treated, CDDO-Me-treated, NEC-1-treated, ACV and CDDO-Me-co-treated, and ACV and NEC-1-co-treated organoids. GAPDH serves as a loading control and GFP indicates virus expression (bottom). **b**, Semi-quantification of HSV-1–GFP (left) and p-p65 protein expression (right) by densitometry in uninfected and 3 dpi HSV-1-infected mock-treated, ACV-treate, CDDO-Me-treated, NEC-1-treated, ACV and CDDO-Me-co-treated, and ACV and NEC-1-co-treated organoids, normalized to GAPDH expression. *P* values were determined by Benjamini–Hochberg-corrected two-sided *t*-tests (*n* = 5 biologically independent samples for CTRL, HSV1 + CDDO-Me and HSV1 + NEC-1, *n* = 6 for all others; colour indicates cell line of origin). Error bars indicate the mean ± standard error. **c**, Representative western blots analysis of p-p65 expression in uninfected (mock) organoids and 3-dpi organoids infected with wild-type (WT) HSV-1 KOS strain or HSV-1 KOS ICP27 mutant strain. GAPDH serves as a loading control and ICP0 indicates virus expression (bottom). **d**, Semi-quantification of HSV-1-ICP0 (left) and p-p65 protein expression (right) by densitometry in uninfected and 3-dpi organoids infected with wild-type HSV-1 KOS or HSV-1 KOS ICP27 mutant strain. *P* values were determined by Benjamini–Hochberg-corrected paired two-sided *t*-tests (*n* = 5 biologically independent samples coming from *n* = 4 batches of medium preparation, indicated by colour). Bar plots and error bars show mean ± standard error. Source data

**Extended Data Fig. 10:**
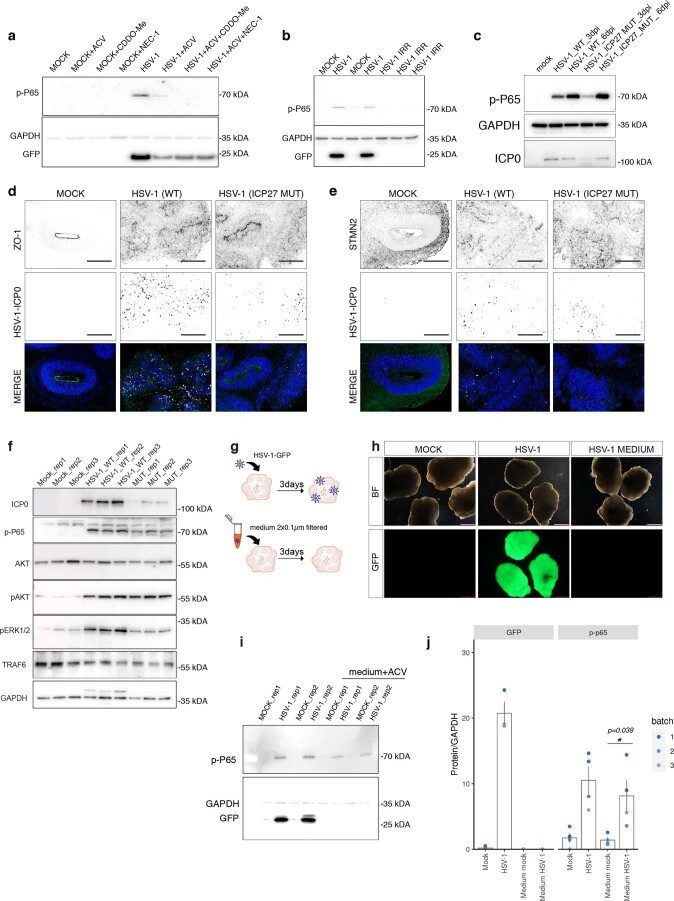
Infected organoids release signaling molecules capable of NF-κB pathway activation independently from viral infection. **a**, Exemplary western blots analysis of p-P65 expression in uninfected (MOCK), mock treated (ACV, CDDO-Me, NEC-1), infected (HSV-1), infected and treated (HSV-1+ACV, HSV-1+ CDDO-Me, HSV-1+ NEC-1) organoids. GAPDH – loading control and GFP -virus expression, n=3 independent experiments. **b**, Exemplary western blots analysis of p-P65 expression in uninfected (MOCK), infected (HSV-1), and organoids exposed to irradiated virus (HSV-1 IRR). GAPDH -loading control, GFP -virus expression (n=3 independent experiments). **c**, Exemplary western blots analysis of p-P65 expression in uninfected (MOCK) and HSV-1- WT virus (3dpi and 6dpi) or HSV-1-ICP27 mutant virus (3dpi and 6dpi) infected organoids. GAPDH – control, ICP0 - virus expression. n=3 independent experiments. **d, e**, Exemplary images of ZO-1 (d) and STMN2 (e) and HSV-1 ICP0 virus expression in: uninfected, mock treated organoids, and HSV-1- WT virus (3dpi) or HSV-1-ICP27 mutant virus (6dpi) infected organoids. Lower panel: merged images, ZO-1/STMN2 (green), ICP0 (white), DAPI (blue). Scale bar - 100 µm. Representative of more than three independent experiments. **f**, Exemplary western blots analysis of p-P65, pAKT, AKT, TRAF6, ERK1/2 expression in uninfected mock organoids, infected with HSV-1- WT virus and HSV-1-ICP27 mutant virus. GAPDH -loading control, ICP0 -virus expression, n=3 biological replicated. Samples were processed in parallel on several blots (see source data ED Fig. 10f). **g**, A scheme of experimental set-up. **h**, Exemplary images of HSV-1 GFP expression in infected organoids or organoids exposed to the medium (2x 0.1 µm filtered) collected from 3dpi infected organoids. **i**, Exemplary western blots analysis of p-P65 expression in uninfected mock (MOCK), HSV-1-GFP infected and organoids exposed to mock or infected organoid medium (MOCK medium, HSV-1 medium, respectively). GAPDH - loading control and GFP - virus expression (lower panel). **j**, Semi-quantification of p-P65 (right panel) and GFP (left panel) protein expression by densitometry in uninfected mock (MOCK), HSV-1-GFP infected and organoids exposed to mock or infected organoid medium (MOCK medium, HSV-1 medium, respectively). Data were normalized on the same sample in each batch. P-values are indicated according to Benjamini-Hochberg-corrected paired two-sided t-tests (n=4 biologically independent samples coming from n=3 batches of medium preparation, indicated by color). Barplots and error bars show mean ± s.e. Source data

To assess whether the combinatorial treatment suppresses HSV-1-driven neuroinflammation, we analysed p65 phosphorylation in treated and uninfected organoids. With both iPS cell lines, p65 phosphorylation was strongly induced at 3 dpi and partially reduced by ACV treatment (Fig. 6a,b), as previously shown in Fig. 4. Co-treatment with either CDDO-Me or NEC-1 significantly strengthened this effect. Treatment with CDDO-Me or NEC-1 alone did not reduce NF-κB–RelA pathway activation, indicating again that inhibition of viral replication is a prerequisite for effective treatment (Fig. 6a,b and Extended Data Fig. 10a). Also, no effect was observed on p-p65 phosporylation with irradiated (inactive) HSV-1–GFP virus (Extended Data Fig. 10b). These results show that anti-inflammatory treatment in combination with antiviral therapies can ameliorate the cellular and tissue damage induced by HSV-1 infection.

To determine whether the HSV-1-driven inflammation is directly dependent on HSV-1 replication, we compared wild-type HSV-1 with a mutant lacking ICP27, an essential protein for the expression of late genes and viral DNA synthesis. Because of its replication defect^38^, we compared p65 phosphorylation in organoids infected with wild-type virus for 3 days with those infected with the mutant strain for 6 days. Six days of infection with the mutant strain were sufficient to observe viral protein levels similar to those at 3 days with the wild-type virus (Extended Data Fig. 10c). However, already at 3 dpi the p65 phosphorylation level was increased in the mutant strain, despite the viral protein levels being lower than in the wild-type strain (Fig. 6c,d). Furthermore, *STMN2* expression and neuroepithelium integrity (ZO-1 expression) were affected by both viruses, but here the effect was weaker in the mutant than in the wild-type strain (Extended Data Fig. 10d,e). These observations suggest that the activation of NF-κB and the related phenotypic consequences on tissue integrity were at least partially independent of viral replication. We further investigated the activation of upstream factors leading to NF-κB p65 phosphorylation and observed that both wild-type and the ICP27 deletion mutant lead to activation of p-AKT and ERK1/2 (Extended Data Fig. 10f), supporting the hypothesis that viral replication is not prerequisite for activation of inflammatory responses.

To assess whether p65 phosphorylation in infected organoids is mediated by signalling molecules released during viral infection, we exposed naive brain organoids to the medium collected from infected organoids depleted of virus via two rounds of filtration with 0.1 µm filters (Extended Data Fig. 10g). No viral protein was detected in these organoids by western blot analysis (Extended Data Fig. 10h,i) and only a few infective viral particles were detected in the medium by plaque assay (~30 viral particles per organoid). p65 phosphorylation in these organoids was significantly elevated compared with mock medium exposed organoids (Extended Data Fig. 10i,j), suggesting that infected organoids release signalling molecules capable of NF-κB pathway activation independently from viral infection.

## Discussion

A comprehensive understanding of the pathogenesis of HSV-1-driven encephalitis and its association with brain tissue damage is still largely elusive. We demonstrated that human brain organoids, combined with a broad range of RNA, protein and electrophysiological analyses, can contribute to a better understanding of HSV-1 pathogenesis and serve as a model to study potential therapeutic strategies.

Our data indicate several HSV-1-induced defects at the molecular level, which may be related to brain tissue damage and neurodegeneration observed in patients with encephalitis^2^. Firstly, downregulation of synaptic proteins and the key axonal maintenance factor STMN2 may be associated with neuronal damage^28,29^. Calcium imaging data also supported that HSV-1 infection very rapidly alters neuronal function. Secondly, activation of TNF–NF-κB signalling stood out in an exploratory analysis of scRNA-seq data, indicating inflammation as a factor contributing to brain tissue damage. The immune response induced by HSV-1 during the early phase of infection is able to limit viral replication, but can also lead to sustained and brain-damaging neuroinflammation^39^. Antiviral ACV treatment, which is commonly prescribed in clinical settings for patients suspected of encephalitis, was able to significantly reduce viral replication, but not TNF–NF-κB signalling. Sustained inflammation can be toxic to neuronal cells, an effect potentiated by activated immune cells. This could also explain the fact that 70% of ACV-treated patients with HSE do not completely recover neurological functions and suffer from cognitive deficits^3^.

It has been previously shown in a mouse model that HSV-1 induces expression of TNF^40^, and that co-treatment with etanercept (an antibody toTNF) or glucocorticoids increased the survival rate of HSV-1-infected mice compared with those only treated with antiviral drugs^41,42^. Consequently, application of immunomodulatory strategies for HSE is currently being discussed^3^. Our data support this notion and suggest that antiviral treatment alone is not sufficient to treat HSE, and that a combinatorial therapy with anti-inflammatory drugs targeting the TNF–NF-κB pathway may be a valuable addition to current therapeutic approaches.

So far, mostly intranasal or intracerebral infection mouse models have been used to study HSE and the infection pattern observed was more diffuse than that observed in patients with HSE, indicating that the mechanism of virus spreading can be different in humans^40^. For example, the virus did not spread to the temporal and frontal lobes, which are the typical locations in HSE in humans^40^. HSV-1 is a human-specific pathogen, and therefore human iPS cell-derived brain organoids can be very valuable to more precisely model virus–host interaction, to develop preventive and therapeutic treatments. Of note, organoids can also be an improvement to 2D neuron culture, as, for example, prolonged infections or latency are difficult to model in 2D culture but possible in organoids^5^.

As a major limitation, we would like to emphasize that the brain organoids used in this study capture only some aspects of the complexity of the human brain, as they lack immune cells and blood vessels and better resemble foetal than adult brain tissues^43^. This, however, enables the study of a ‘pure’ neuronal immuno-defensive state, which is becoming a central topic in the field of neuroimmunology^44^. Indeed, we did observe a strong activation of the innate inflammatory cellular response, reflected by TNF–NF-κB pathway activation. This was accompanied by a lower and cell-type-specific interferon pathway activation. A complete lack of interferon activation was previously reported in early-stage HSV-1-infected organoids^16^, where treatment with exogenous IFNα2 was able to inhibit viral replication and thus prevent growth defects in this model. More complex brain organoids containing also immune cells may therefore be an important step forward to better understand infections with neuroinvasive viruses.

## Methods

### Study design

In this study, we used human cerebral organoids as a model for acute HSV-1. The aim of the study was as follows: (1) to assess cellular and molecular signatures associated with viral infection; (2) to study the outcome of the current antiviral therapy used in clinical practice (ACV); (3) to identify and test better potential therapeutic approaches based on the results of 1 and 2. We used two genetically distinct human iPS cell lines and performed exploratory transcriptomic analyses (bulk RNA-seq and scRNA-seq) in two replicates and three conditions (controls and two timepoints of infection, three replicates per condtion). For scRNA-seq, we additionally performed two replicates per line of infected and ACV-treated organoids. Drug concentrations as well as timepoints of infection were determined on the basis of pilot experiments assessing virus spreading over time, with the aim of modelling acute infection. Validation experiments and additional characterization experiments (western blots, immunhistochemistry and calcium imaging), for example, for assessing the outcome of anti-inflammatory treatments, were performed in two to five biological replicates per cell line. Organoids were assigned blindly to the various experimental groups. Data analysis could not be performed blind to the conditions of the experiments due to the GFP signal detection in infected organoids. No statistical methods were used to pre-determine sample sizes, but our sample sizes are similar to those reported in previous publications^16^.

### iPS cell lines

The human iPS cell lines iPSC-1 XM001 (ref. ^45^) and iPSC-2 (A18945, Thermo Fisher Scientific) were cultured in standard hypoxic conditions (37 °C, 4% CO_2_, 4% O_2_ and 100% humidity), in E8 Flex medium (Thermo Fisher Scientific).

### Generation of cerebral organoids

We generated iPS cell-derived cerebral organoids according to a protocol previously described with some modifications^7^. Shortly, after dissociation into single-cell suspension with accutase, we seeded 6,000 cells per one well of 96-well plates in 100 µl of embryoid body (containing Dulbecco’s modified Eagle medium (DMEM)/F12, 20% knockout replacement serum, 1× Glutamax, 1× Minimal Essential Medium-Non Essential Amino Acid (MEM-NEAA), 2% embryonic stem cell-qualified fetal bovine serum (FBS), 50 µM ROCK inhibitor, 10 µM basic fibroblast growth factor or STEMdiff Cerebral Organoid Basal Medium supplemented with Supplement A (STEMCELL Technologies) medium. After 4 days, we replaced the medium with EB medium without basic fibroblast growth factor and ROCK inhibitor. On day 5, we replaced the medium with a neural induction medium (DMEM/F12, 1× N2 supplement, 1× Glutamax, 1× MEM-NEAA, 10 µg ml^−1^ heparin solution). At days 7–9, we performed a liquid embedding of organoids into 2% Matrigel (Corning, 356234) and kept them in neural induction medium for 2 days, and in organoid differentiation medium containing 1:1 DMEM/F12: Neurobasal, 1× N2 supplement, 1× B27 + vitamin A supplement, insulin, 2-mercaptoethanol solution, Glutamax, MEM-NEAA and CHIR99021 for another four days. Next, we transferred the organoids to ultralow-attachment six-well plates and cultured them on an orbital shaker (80 r.p.m.) in organoid maturation medium containing 1:1 DMEM/F12:Neurobasal, N2 supplement, B27 + vitamin A supplement, insulin, 2-mercaptoethanol solution, Glutamax supplement, MEM-NEAA, sodium bicarbonate, vitamin C solution, chemically defined lipid concentrate, brain-derived neurotrophic factor (BDNF), glial cell line-derived neurotrophic factor (GDNF), cAMP and 1% Matrigel. Live organoid imaging was performed using NICON Eclipse Ts2 microscope.

### Viral infections

The following HSV-1 strains were employed in this study: strain 17 containing GFP under an MCMV promoter^46^ KOS (VR-1493; ATCC), and a KOS-based ICP27 knockout virus^38^. Around five to eight organoids cultured in six-well plates were infected with 75,000 or 15,000 plaque-forming units (PFU) of HSV-1 particles per organoid (corresponding to an MOI of 1 as calculated from the estimated number of cells at the surface of the organoids, or MOI of 0.2), and 24 h after the medium was replaced with fresh organoid maturation medium and organoids were cultured for an additional 1 or 3 days (1 dpi/3 dpi). The control organoids were culture in parallel in the same conditions, but without viral infection. For the anti-inflammatory treatment, around five to eight organoids cultured in six-well plates were infected with an MOI of 1 or MOI of 0.2, and 8–12 h after the medium was replaced with fresh organoid maturation medium supplemented with ACV (50 µM), NEC-1 (40 µM) or CDDO-Me (0.8 µM) or combination of ACV and organoids were cultured for an additional 1 or 3 days (1 dpi/3 dpi).

For the infected medium exposure, we infected organoids with 75,000 viral particles per organoid (8 organoids per well) for 3 days. We collected the medium from the infected organoids and filtrated it twice thought a 0.1 µm filter and added the medium to naive (uninfected) organoids by replacing half of the medium. Organoids were incubated with conditioned medium (medium HSV-1) for additional three days in the presence of ACV (to ensure no viral replication) before subequent analyses. The medium from mock-infected organoids, which was processed in the same way from mock-infected organoids, served as control (medium mock).

For ultraviolet irradiation experiment, the HSV-1 GFP virus stock was diluted to about 5 × 10^6^ PFU ml^−1^ in DMEM, and exposed to 254 nm ultraviolet light in 35 mm plastic dishes without lid. We used 10 min irradiation in a conventional Analytik Jena 254 nm crosslinker. After 10 min, the remaining activity of the virus was about 100 PFU ml^−1^.

### Immunostaining of brain organoids

For immunostainings, organoids were washed three times with phosphate-buffered saline (PBS) and fixed in 4% paraformaldehyde for 20–60 min (depending on the organoid size) at 4 °C, then washed with PBS three times for 10 min each. The tissue was incubated in 40% sucrose (in PBS) until it sunk (overnight) and then embedded in 13%/10% gelatin/sucrose. Frozen blocks were stored at −80 °C, before cryosections. Then 10–14 μm sections were prepared using a cryostat. Sections were incubated with warm PBS for 10–15 min to remove the embedding medium and then fixed for additional 10 min with 4% paraformaldehyde, washed three times with PBS and blocked and permeabilized in 0.25% Triton-X, 5% normal goat serum in PBS for 1 h. Sections were first incubated with primary antibodies (Supplementary Table 1) in 0.1% Triton-X, 5% normal goat serum overnight. The following primary antibodies were used: STMN2 (Novus Biologicals, NBP1-49461, 1:1,000), SOX2 (Merck, AB5603, 1:1,000), PAX6 (Biolegends 901301, 1:1,000), ZO-1 (Invitrogen 339100, 1:1,000), NEUN (Sigma-Aldrich, MAB377, 1:500), MAP2 (Sigma-Aldrich, MAB3418A5, 1:1,000), SYN1 (Abcam ab8, 1:1,000), TUJ1 (Sigma-Aldrich T2200, 1:1,000), GFAP (Milipore/Merck, 1:1,000), TBR1 (Abcam, ab183032, 1:1,000), GFP (Abcam, ab13970, 1:1,000), phospho-Vimentin (Biozol Diagnostics, MBL-D076-3, 1:2,000), SATB2 (Abcam ab34735, 1:1,000), C-Caspase3 (Cell Signaling 9661, 1:500) and ICP0 (Santa Cruz SC53070, 1:500). They were then washed three times for 10 min each with PBST (0.1% Triton X-100) and incubated with secondary antibodies (Alexa 488 anti-chicken (Abcam 150169, 1:500), Alexa 568 anti-mouse (Abcam ab157473, 1:500), Alexa 647 anti-mouse (Abcam ab150115, 1:500), Alexa 568 anti-rabbit (Abcam ab175471, 1:500), Alexa 647 anti-rabbit (Abcam ab150083, Alexa 647 anti-goat (Thermo Fisher A21-447 1:500)) at room temperature for 2 h, followed by staining with 4′,6-diamidino-2-phenylindole (DAPI) (final 1 µg ml^−1^) for 10 min and washed three times with PBST. The images were acquired using a Keyence BZ-X710 microscope.

### Western blotting

Organoids were lysed in RIPA buffer (150 mM NaCl, 5 mM EDTA, 50 mM Tris, 1% NP-40, 0.5% sodium deoxycholate and 0.1% SDS) in the presence of protease and phosphatase inhibitors. Subsequently, the samples were incubated on ice for 30 min and centrifuged at 14,000*g* for 20 min at 4 °C. The total protein concentrations were determined using the Pierce BCA assay (Thermo Fisher Scientific, 23225). Samples (10–20 µg) were boiled in standard SDS–PAGE sample buffer supplemented with 100 mM dithiothreitol, boiled at 95 °C and resolved in a 10–12% SDS–PAGE. Proteins were transferred onto Transblot Turbo Midi PVDF membranes (Bio-Rad, 1704157). The membrane was blocked with Tris-buffered saline containing 0.1% Tween-20 (TBST, Sigma-Aldrich) supplemented with 5% skimmed milk for 1 h at room temperature. Subsequently, the membranes were incubated with the primary antibodies (Supplementary Table 1) diluted in TBST with skimmed milk at 4 °C overnight. The following primary antibodies were used: AKT (Cell Signaling 9272, 1:1,000), p-AKT (Cell Signaling 9277S, 1:1,000), SYN1 (Abcam ab8, 1:1,000), TRAF6 (Santa Cruz 7221, 1:1,000), ICP0 (Santa Cruz SC53070, 1:800), GFP (Merck G1546, dilution 1:1,000), pERK1/2 (Cell Signaling 9102, dilution western blot: 1:1,000), HOMER1 (Synaptic Systems, 160011, 1:1,000), p-p65 (Cell Signaling 3033 T, dilution western blot: 1:2,000) and GAPDH (Sigma G8795, 1:2,000). After washing in TBST, the membranes were incubated with anti-rabbit or anti-mouse horseradish peroxidase-conjugated secondary antibody (secondary antibodies: goat anti-mouse IgG (H+L) HRP (Thermo Fisher Scientific 31430, 1:8,000), polyclonal goat anti-rabbit HRP, Dako P0448, 1:5,000)) for 1 h at room temperature. Blots were then developed with AmershamTM ECL select reagent (Cytiva, RPN3243) and visualized by Fusion FX (Vilber). Band intensities were quantified using ImageJ.

### RNA sequencing

For the total RNA sequencing we used 100 ng of total RNA, where ribosomal RNA was depleted using RNase H-based protocol. We mixed total RNA with 1 μg of a DNA oligonucleotide pool comprising 50-nt-long oligonucleotide mix covering the reverse complement of the entire length of each human rRNA (28S rRNA, 18S rRNA, 16S rRNA, 5.8S rRNA, 5S rRNA and 12S rRNA), incubated with 1 U of RNase H (Hybridase Thermostable RNase H, Epicentre), purified using RNA Cleanup XP beads (Agencourt), DNase treated using TURBO DNase rigorous treatment protocol (Thermo Fisher Scientific) and purified again with RNA Cleanup XP beads. We fragmented the rRNA-depleted RNA samples and processed them into strand-specific complementary DNA libraries using TruSeq Stranded Total LT Sample Prep Kit (Illumina) and then sequenced them on NextSeq 500, High Output Kit, 2 × 76 cycles. We performed differential gene expression analysis using the DESeq2 (version 1.20.00) R package.

### RNA-seq data analysis

Bulk RNA sequencing data were analysed using the PiGx-RNA seq pipeline version 0.0.3 (ref. ^47^). Briefly, RNA-seq reads were mapped to the custom human-viral reference genome using STAR version 2.7.3a (ref. ^48^) and those uniquely mapping to exons have been used for gene expression quantification. Default settings were used, with the exception of ‘–outFilterMismatchNoverLmax 0.05’. Reads were counted using htseq-count (version 0.9.1; ref. ^49^) with GENCODE v27 genome annotation reference^50^.

### Differential gene expression analysis

Differential gene expression analysis was done using DESeq2 version 1.34.0 (ref. ^51^), using default options, and multi-factor design that measures the effect of infection while accounting for differences between cell lines (design = ~ line + group). Significance threshold was set to adjusted *P* value of 0.05. The list of TNF ligands was obtained from the HUGO Gene Nomenclature Committee^52^ database under the gene group ‘tumour necrosis factor (ligand) superfamily’. The list of synaptic genes was obtained from the Gene Ontology database under the biological process ‘chemical synaptic transmission’.

### Antisense transcription analysis

Antisense transcribed regions were identified and defined from STAR-aligned reads using the running sum algorithm previously described^31^. Overlapping antisense transcripts from different RNA-seq libraries were collapsed together. All putative antisense transcripts with an annotated upstream sense gene within 1 kb were discarded as potential readthroughs, and those with a downstream sense gene within 10 kb were discarded as potential transcripts from an upstream transcription start site. We extended the GENCODE v27 genome annotation reference with detected antisense transcripts and counted the mapping reads using htseq-count 0.9.1, as described earlier.

### Tissue processing and Visium data generation

Brain organoid samples, frozen in isopentane and embedded in OCT (TissueTek Sakura) and cryosectioned at −12 °C (Thermo Cryostar). Sections were placed on chilled Visium Spatial Gene Expression Slides (2000233, 10x Genomics). Tissue sections were then fixed in chilled methanol and stained according to the Visium Spatial Gene Expression User Guide (CG000239 Rev A, 10x Genomics). Libraries were prepared according to the Visium Spatial Gene Expression User Guide (CG000239; ref. ^53^). Libraries were sequenced on a NovaSeq 6000 System (Illumina) using a NovaSeq S4 Reagent Kit (200 cycles, 20027466, Illumina), at a sequencing depth of approximately 250–400 M read pairs per sample. Sequencing of 10 µm organoid slices (four organoids per condition) yielded, for uninfected organoids, on average 8,000 UMIs, which quantified ~3,700 genes per spot. Data were analysed using the Spaceranger 1.2.0 software (10x Genomics).

### scRNA-seq

Methanol-fixed cells were centrifuged at 3,000–5,000*g* for 5 min, rehydrated in 1 ml PBS + 0.01% bovine serum album (BSA) supplemented with RNAse inhibitors (1 unit μl^−1^ RiboLock, ThermoFisher), pelleted and resuspended again in 0.5 ml PBS + 0.01% BSA in the presence of RNAse inhibitors. Cells were manually counted by means of a haemocytometer and diluted to a suspension of typically ~300 cells μl^−1^ in PBS + 0.01% BSA. Cells were encapsulated together with barcoded microparticles (Macosko-2011-10 (V+), ChemGenes Corp.) using the Dolomite Bio Nadia instrument, using the standard manufacturer’s dropseq-based^54^ scRNA-seq protocol.

Droplets were broken immediately after collection and cDNA libraries generated as previously described^54^. First-strand cDNA was amplified by equally distributing beads from one run to 24 Smart PCR reactions (50 μl volume; 4 + 9–11 cycles). Then 20 μl fractions of each PCR reaction were pooled (total 480 μl), then double-purified with 0.6× volumes of AMPure XP beads (Beckman Coulter). Amplified cDNA libraries were assessed and quantified on a BioAnalyzer High Sensitivity Chip (Agilent) and the Qubit dsDNA HS Assay system (ThermoFisher). Then 600 pg of each cDNA library was fragmented, amplified (13 cycles) and indexed for sequencing with the Nextera XT v2 DNA sample preparation kit (Illumina) using custom primers enabling 3′-targeted amplification. The libraries were purified with AMPure XP Beads, quantified and sequenced on Illumina NextSeq500 sequencers (library concentration 1.8 pM; NextSeq 500/550 High Output v2 kit (75 cycles) in paired-end mode; read 1 = 20 bp using the custom primer Read1CustSeqB^49^ read 2 = 64 bp).

### scRNA-seq data analysis

#### Quality control and data pre-processing

Raw sequencing data were converted to fastq format using bcl2fastq (v.2.19). Single-cell digital gene expression matrices (DGEs) were generated using the spacemake pipeline (v.0.4.3)^55^ with Drop-seq tools (v.2.5.0) (https://github.com/broadinstitute/Drop-seq) and STAR (v.2.6.0)^48^. Briefly, read 1 was used to determine the cell barcode (base pairs 1–12) and the UMI (13–20), while read 2 was mapped to a custom human-viral reference genome built by simple concatenation of the human (hg38) and HSV1 genomes (barcode_flavors: default, run mode: scRNA_seq). For the HSV-1 genome, we started out from the known previously published sequence^24^ and refined it on the basis of the total RNA-seq data. For the mapping, we removed the terminal repeats to avoid multimappers. The employed sequence and annotation is available on the National Center for Biotechnology Information Gene Expression Omnibus entry accompanying this manuscript (GSE163952).

Only reads mapping to exons have been used for gene expression quantification (count_intronic_reads: false) and only the 10,000 barcodes with the highest UMI counts have been used for downstream analyses (n_beads: 10,000). DGEs were further analysed in R (v4.1) using Seurat (v.4.0.4)^56^. We initially adopted a very conservative filtering strategy removing cells with fewer than 250 detected genes and genes detected in fewer than 5 cells (CreateSeuratObject function with min.cells of 5 and min.features of 250). We normalized gene expression and scaled gene expression for each individual organoid (SCTransform function with method ‘glmGamPoi’ and vst.flavor ‘v2’)^57^. We then integrated cells from control organoids to analyse cell composition at baseline and integrated DGEs from all organoids to analyse the response to infection. To do so, we selected the 3,000 genes most variable across the datasets (SelectIntegrationFeatures function with nfeatures 3,000), computed missing residuals (PrepSCTIntegration function), identified cells representing mutual nearest neighbours^58^ or pairwise anchors between datasets (FindIntegrationAnchors function)^59^ and finally performed iterative pairwise dataset integration starting from the most similar ones (IntegrateData function). We then performed dimensionality reduction (RunPCA function) and selected the first 30 principal components to identify shared nearest neighbours (FindNeighbors with dims 1:30), clustering (FindClusters function with resolution 0.4 for control organoids and 0.8 for all organoids) and compute a two-dimensional embedding to visualize the data (RunUMAP with dims 1:30). We removed two control clusters (clusters 3 and 14) and four clusters across all organoids (clusters 6 and 10) that were characterized by a substantially lower UMI count and/or higher mitochondrial UMI percentage. Furthermore, to remove low-quality cells in a cell-type-specific way, we removed cells with UMI counts below the 25th percentile in each cluster. Afterwards, we repeated gene expression normalization, integration, dimensionality reduction and clustering as described above and identified 16 clusters in control organoids (resolution 0.4) and 20 across all organoids (resolution 0.8).

### Cluster annotation

We identified marker genes in control organoids clusters (FindAllMarkers with only.pos = T) and annotated each cluster leveraging published references^21,60,61^. We relied both on overlapping marker genes between our control clusters and published references and on label transfer results for ref. ^21^. Label transfer was performed using the standard Seurat pipeline. Briefly, a subset of ‘features’ (that is, genes) identified by the SelectIntegrationFeatures function with default parameter and then pairs of ‘anchors’ (that is, cells) between the two datasets have been identified using the FindTransferAnchors function (normalization.method ‘SCT’, reference.assay ‘SCT’, query.assay ‘integrated’, reduction ‘pcaproject’, dims 1:20, features ‘features’, nn.method ‘rann’, eps 0.5) and finally the TransferData function (anchorset ‘anchors’, prediction.assay ‘TRUE’, weight.reduction ‘pcaproject’, dims 1:20, eps 0.5) was leveraged to score each of the cells in the query dataset for similarity with labelled cell types in the reference dataset. Finally, LabelTransfer scores were inspected and analysed together with marker gene expression to annotate clusters in control organoids. To annotate clusters in all organoids, we leveraged the annotation of control cells and used the same annotation for the whole cluster when control cells accounted for at least 33% of the cluster or at least 10% but accounted for at least 50% of the specific control cluster. The remaining clusters have been annotated as ‘highly infected’ and ‘hindbrain’ based on the expression of viral and HOX genes, respectively. We further removed control cells falling in ‘highly infected’ clusters as they exhibited substantially lower UMI counts.

### Differential gene expression and GSEA

To identify differentially regulated genes upon infection in robust and reproducible way, we performed a pseudobulk analysis using the edgeR package^62^. For selected clusters, we generated a pseudobulk expression profile for each organoid by summing the expression of 50 randomly selected cells (with replacement). We then used a multifactorial design to account for intercell line differences (function model.matrix(~line + condition)). We then filtered genes with low expression values (filterByExpr function with min.count 10 and min.total.count 50), normalized expression values by library size (calcNormFactors function), estimated negative binomial dispersions (estimateDisp function) and fitted a generalized linear model (glmQLFit function). For each comparison (1 dpi versus CTRL, 3 dpi versus CTRL, 3 dpi ACV versus CTRL), we identified differentially expressed genes (glmQLFTest function) and adjusted *P* value was calculated using Bonferroni correction for multiple testing correction (p.adjust function). To identify enriched pathways associated with HSV infection, we performed GSEA using clusterProfiler^63^. For each cluster and each comparison, genes were ranked according to decreasing log fold change to perform gene set enrichment against the Hallmark gen sets (GSEA function). Only pathways with a Benjamini–Hochberg-adjusted *P* value lower than 0.05 were considered as significantly enriched.

### Calcium imaging

For calcium imaging experiments, organoids were plated in 35 mm glass-bottom imaging dish coated with poly-d-lysine and Geltrex. Two days after plating, organoids were loaded with 5 μM CalBryte590 AM (AAT Bioquest) and 0.02% pluronic acid in organoid maturation medium for 30 min at 37 °C. Imaging was performed in a Dragonfly spinning disk confocal microscope (Andor, Oxford Instruments) at a frequency of 5 Hz for 5 min. The resulting images were then background subtracted, and the relative changes in intensity over time were computed. Activity-based segmentation was performed as previously described^64^. Spike detection was performed on the intensity traces from each region of interest (ROI) to calculate action potential firing frequency.

### Statistical analysis

No statistical methods were used to pre-determine sample sizes, but our sample sizes are similar to those reported in previous publications. Data distribution was assumed to be normal for datasets with a low number of replicates (*n* < 10), but this was not formally tested, while non-parametric tests were applied to larger datasets. Relevant details of the statistical tests used for each comparison as well as multiple testing corrections are provided for all relevant panels in the figure legends and reported below.

#### Western blot quantification

Band intensities were quantified using ImageJ (version 1.52q, functions from Analyze/Gels toolbar). Statistical significance of the observed differences between each condition was evaluated, after normalization to GAPDH, with a Benjamini–Hochberg-corrected *t*-test in RStudio Server v.1.4.1106 (R v.4.1.2).

#### Immunostaining quantification

Images were then analysed by quantifying the fluorescent signal in each channel with a jlm script within fiji imagej 2.0.0-rc-69/1.53 f/java1.8.8_172 (64 bit), after background subtraction with a rolling ball radius of 20. Statistical significance of the observed differences between each condition was evaluated with a Benjamini–Hochberg-corrected rank-sum test, with the pairwise.wilcox.test, function in RStudio v.1.2.5033 (R v.4.1.2).

#### scRNA-seq

Marker genes used for the annotation of each single-cell cluster were identified using a Wilcoxon rank-sum test comparing the expression of all genes in the cluster of interest with all the remaining cells as implemented in the FindAllMarkers function in the Seraut R package (v.4.0.4). Differentially expressed genes in the different conditions were identified for selected clusters using a quasi-likelihood negative binomial generalized linear model as implemented in the glmQLFit function in the edgeR R package (v.3.36.0). Computed *P* values were adjusted using Bonferroni correction for multiple testing. Enriched gene sets have been identified as implemented in the GSEA function in the clusterProfiler R package (v.4.2.2), and only pathways with a Benjamini–Hochberg-adjusted *P* value lower than 0.05 were considered as significantly enriched.

### Reporting summary

Further information on research design is available in the Nature Portfolio Reporting Summary linked to this article.

## Supplementary information

Supplementary Information Supplementary Figs. 1–3, Table 1 and figure legends.

Reporting Summary

Peer Review File

Supplementary Video 1 Calcium imaging in 60-day-old organoids.

Supplementary Video 2 Calcium imaging in 60-day-old HSV-1-infected organoids (2 dpi).

## Data Availability

Single-cell and bulk RNA-seq data from human brain organoids infected with HSV-1, at different timepoints post infection, are available at the National Center for Biotechnology Information under Gene Expression Omnibus identifier GSE163952. Source data are provided with this paper.

## Source data

Source Data Fig. 3 Statistical source data.
Source Data Fig. 4 Unprocessed western blots.
Source Data Fig. 4 Statistical source data.
Source Data Fig. 6 Unprocessed western blots.
Source Data Fig. 6 Statistical source data.
Source Data Extended Data Fig. 2 Unprocessed western blots.
Source Data Extended Data Fig. 2 Statistical source data.
Source Data Extended Data Fig. 8 Statistical source data.
Source Data Extended Data Fig. 9 Unprocessed western blots.
Source Data Extended Data Fig. 9 Statistical source data.
Source Data Extended Data Fig. 10 Unprocessed western blots.
Source Data Extended Data Fig. 10 Statistical source data.

## Code availability

Scripts for the analysis of single-cell and bulk RNA-seq data and the generation of the related figure plots are publicly available on GitHub (https://github.com/rajewsky-lab/HSV1_cerebral_organoids).
