# Supplementary material for: Modelling viral encephalitis caused by herpes simplex virus 1 infection in cerebral organoids
Source: Nat Microbiol. 2023 Jun 22;8(7):1252–66. doi: 10.1038/s41564-023-01405-y (PMC10322700; doi:10.1038/s41564-023-01405-y)
Supplement: Supplementary file 1 — Supplementary Figs. 1–3, Table 1 and figure legends. [file 41564_2023_1405_MOESM1_ESM.pdf]

nature microbiology

Article https://doi.org/10.1038/s41564-023-01405-y

Modelling viral encephalitis caused by herpes simplex virus 1 infection in cerebral organoids

In the format provided by the authors and unedited

Supplementary Fig. 1

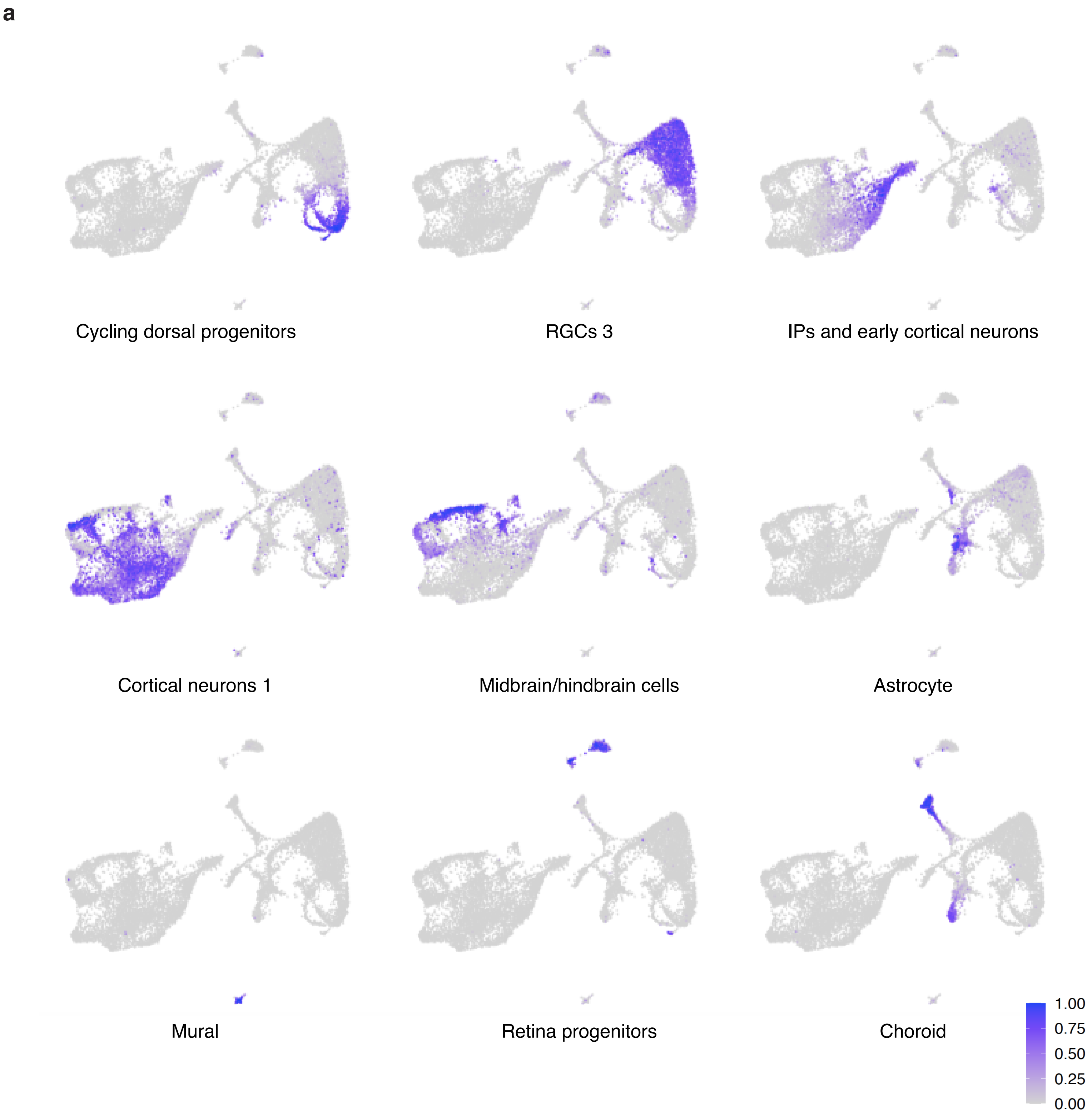

Supplementary Figure 2

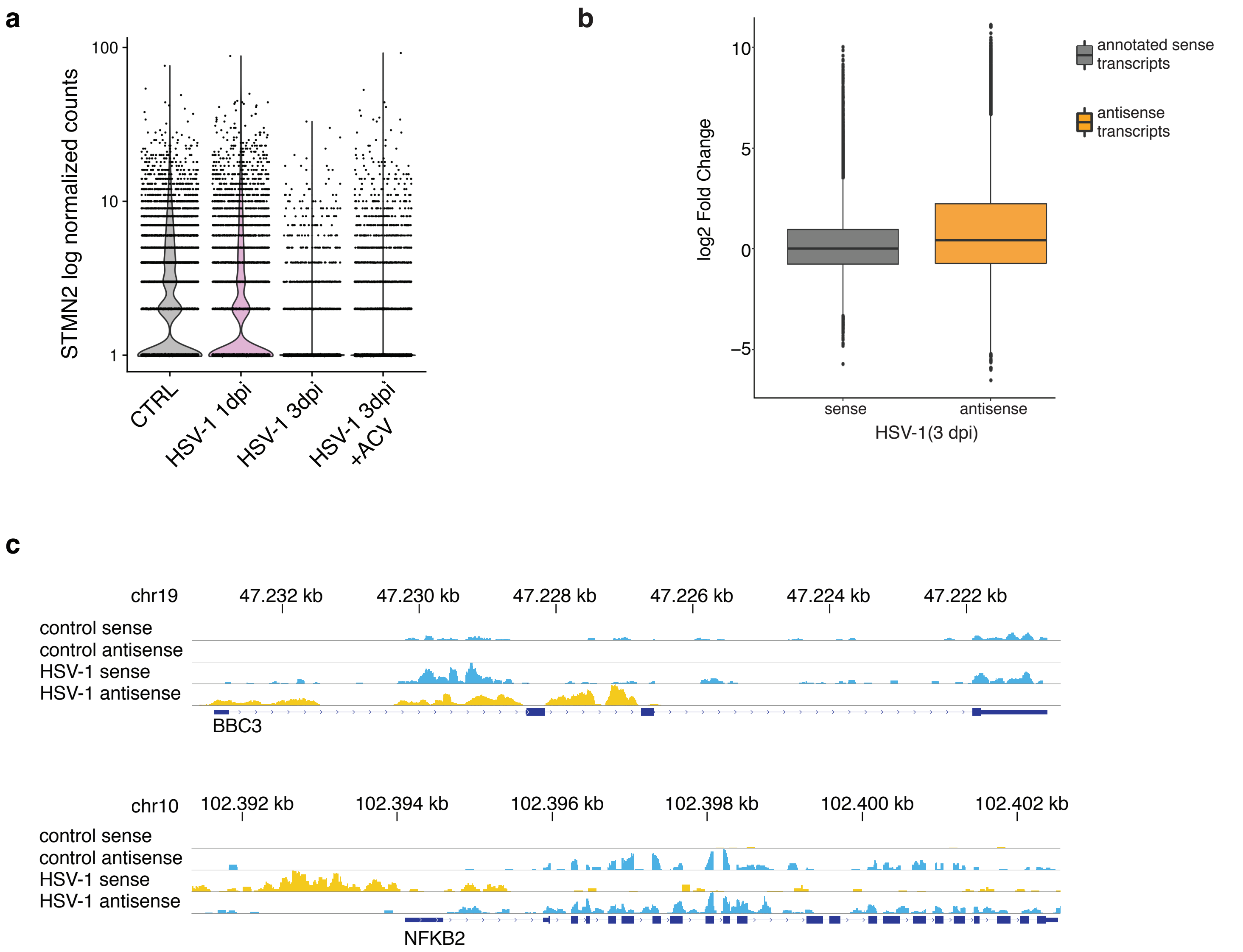

Supplementary Fig.3

g

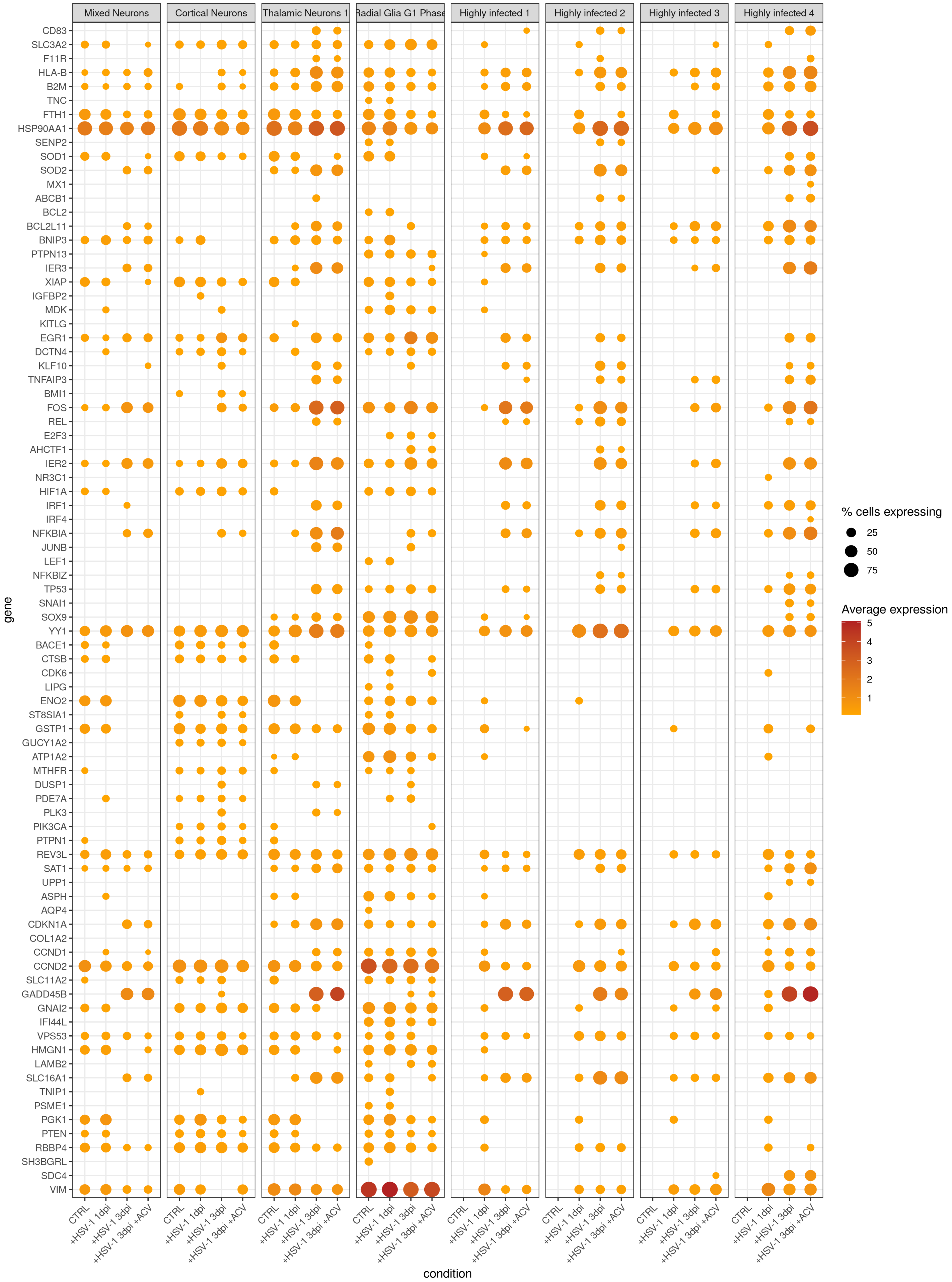

Table 1. Antibodies list

Antibody	Supplier	Dilution
STMN2	Novus Biologicals, NBP1-49461	IS: 1:1000
SOX2	Merck, AB5603	IS: 1:1000
PAX6	BioLegend, 901301	IS: 1:1000
ZO-1	Thermo Fischer Scientific, 339100	IS: 1:1000
NeuN	Sigma-Aldrich, MAB377	IS: 1:500
MAP2	Sigma-Aldrich, MAB3418A5	IS: 1:1000
SYN1	Abcam, ab8	IS: 1:1000
TUJ1	Sigma T2200	IS: 1:1000
GFAP	Milipore/Merck AB5804	IS: 1:1000
TBR1	Abcam, ab183032	IS: 1:1000
phosopho-p65 (p-p65)	Cell Signaling, 3033T	WB: 1:2000, IS: 1:1000
GFP	Abcam, ab13970	WB: 1:2000, IS:1:1000
Phosphor-Vim (pVIM)	Biozol Diagnostics, MBL-D076-3	IS: 1:2000
SATB2	Abcam, ab34735	IS: 1:1000
AKT	Cell Signaling, 9272	WB: 1:1000
pAKT	Cell Signaling, 9277S	WB: 1:1000
TRAF6	Santa Cruz sc-7221	WB: 1:1000
ICP0	Santa Cruz sc-53070	WB: 1:800, IS:1:500
pERK1/2	Cell signaling 9102	WB: 1:1000
C-Caspase3	Cell signaling 9661	IS: 1:500
Secondary antibodies		
Goat anti-Mouse IgG (H L) HRP	Thermo Fisher Scientific 31430	1:8000
Polyclonal Goat Anti rabbit HRP	Dako P0448	1:5000
Alexa 488 anti chicken	Abcam ab150169	1:500
Alexa 568 anti mouse	Abcam ab157473	1:500
Alexa 647 anti mouse	Abcam ab150115	1:500
Alexa 647 anti goat	Thermo Fisher Scientific A-21447	1:500
Alexa 568 anti rabbit	Abcam ab175471	1:500
Alexa 647 anti rabbit	Abcam ab150083	1:500

Supplementary data Figure legends

Supplementary data Fig. 1 Cerebral organoids development at single cell and spatial resolution.

a, Comparison of annotations CTRL organoids with a published reference atlas (Kanton et al, REF). 9 UMAP plots are shown with cells colored by their Label Transfer score, which indicates their similarity to selected clusters in the reference atlas.

Supplementary data Fig. 2 HSV-1 activates antisense transcription.

a, Single-cell mRNA expression of stathmin-2 (*STMN2*). Each dot represents a single cell. Statistically significant gene expression is observed only if a violin-shaped fitting area can be calculated. (Merged data from two independent iPSC lines, each n=2). **b,** Box plots showing log2 fold changes of sense annotated genes (grey) and antisense transcripts 3 days post infection (dpi) (two independent iPSC lines, each n=3). Boxplot centers represent median values, while the boxplot bounds represent the 25% and 75% quantiles. Boxplot whiskers represent the 25% quantile − 1.5 × interquartile range (IQR) and the 75% quantile + 1.5 × IQR, respectively.

c, Examples of antisense transcripts: NFIC (antisense internal) and POLR3Eas (antisense divergent). Coverage profiles of total RNA-sequencing data from 60 days old cerebral organoids (two independent iPSC lines, each n=3). Sense genes in blue (orange) oriented left to right (right to left). Transcript annotation in dark blue.

Supplementary data Fig. 3 HSV-1 activates inflammatory responses.

a, Dotplot showing the expression of detected direct NFkB targets. Color indicates average, SCT-normalized expression values and dot size shows the percentage of expressing cells in each cluster. Only highly infected clusters and clusters included in the differential gene expression and gene set enrichment analysis are shown. (Merged data from two independent iPSC lines, each n=2).

Supplementary Table 1. List of antibodies

Supplementary video 1. Calcium imaging in 60 days-old organoids. Time lapse series showing the fluctuations of CalBryte590 fluorescence. Images were acquired at 5 Hz for 5 min and relative changes in intensity over time computed.

Supplementary video 2. Calcium imaging in 60 days-old organoids HSV-1 infected organoid (2 dpi). Time lapse series showing the fluctuations of CalBryte590 fluorescence. Images were acquired at 5 Hz for 5 min and relative changes in intensity over time computed.
