# Supplementary material for: Modelling viral encephalitis caused by herpes simplex virus 1 infection in cerebral organoids
Source: Nat Microbiol. 2023 Jun 22;8(7):1252–66. doi: 10.1038/s41564-023-01405-y (PMC10322700; doi:10.1038/s41564-023-01405-y)
Supplement: Supplementary file 2 — Reporting Summary [file 41564_2023_1405_MOESM2_ESM.pdf]

nature portfolio

Corresponding author(s): Rajewsky Nikolaus, Agnieszka Rybak-Wolf

Last updated by author(s): Apr 11, 2023

Reporting Summary

Nature Portfolio wishes to improve the reproducibility of the work that we publish. This form provides structure for consistency and transparency in reporting. For further information on Nature Portfolio policies, see our Editorial Policies and the Editorial Policy Checklist.

Statistics

For all statistical analyses, confirm that the following items are present in the figure legend, table legend, main text, or Methods section.

n/a	Confirmed	
☐	☒	The exact sample size (*n*) for each experimental group/condition, given as a discrete number and unit of measurement
☐	☒	A statement on whether measurements were taken from distinct samples or whether the same sample was measured repeatedly
☐	☒	The statistical test(s) used AND whether they are one- or two-sided<br>*Only common tests should be described solely by name; describe more complex techniques in the Methods section.*
☐	☒	A description of all covariates tested
☐	☒	A description of any assumptions or corrections, such as tests of normality and adjustment for multiple comparisons
☐	☒	A full description of the statistical parameters including central tendency (e.g. means) or other basic estimates (e.g. regression coefficient) AND variation (e.g. standard deviation) or associated estimates of uncertainty (e.g. confidence intervals)
☐	☒	For null hypothesis testing, the test statistic (e.g. *F*, *t*, *r*) with confidence intervals, effect sizes, degrees of freedom and *P* value noted<br>*Give P values as exact values whenever suitable.*
☒	☐	For Bayesian analysis, information on the choice of priors and Markov chain Monte Carlo settings
☒	☐	For hierarchical and complex designs, identification of the appropriate level for tests and full reporting of outcomes
☒	☐	Estimates of effect sizes (e.g. Cohen's *d*, Pearson's *r*), indicating how they were calculated

Our web collection on statistics for biologists contains articles on many of the points above.

Software and code

Policy information about availability of computer code

Data collection	Sequencing was performed on Illumina NextSeq500/NovaSeq 6000 devices, and fastq files generated using bcl2fastq (v. 2.19). Commercial software provided with laboratory equipment: ; Vilber Fusion FX imager (Evolution-CaptEdge - Fusion FX Edge 18.09), NICON Eclipse Ts2, BZ-X Viewer 01.03.01.01 (Keyence)
Data analysis	Fiji imagej 2.0.0-rc-69/1.53f/java1.8.8_172 (64bit); Spaceranger 1.2.0 (10X Genomics); RStudio Server (v. 1.4.1106, with R v. 4.1.2), RStudio (v. 1.2.5033, with R v. 4.1.2); Drop-seq tools (v. 2.5.0), STAR (v. 2.6.0), Seurat (v. 4.0.4), MASS (v.7.3-53), DESeq2 (v. 1.34.0), PiGx-RNA seq pipeline (v. 0.0.3), htseq-count (v. 0.9.1)<br>Additional in-house developed software: Spacemake 0.4.3

For manuscripts utilizing custom algorithms or software that are central to the research but not yet described in published literature, software must be made available to editors and reviewers. We strongly encourage code deposition in a community repository (e.g. GitHub). See the Nature Portfolio guidelines for submitting code & software for further information.

Data

Policy information about availability of data

All manuscripts must include a data availability statement. This statement should provide the following information, where applicable:

- Accession codes, unique identifiers, or web links for publicly available datasets
- A description of any restrictions on data availability
- For clinical datasets or third party data, please ensure that the statement adheres to our policy

All raw and processed sequencing data as well as the HSV-1 annotation used have been deposited on GEO (accession GSE163952). The following database/ ressources were used HUGO gene nomenclature committee (https://www.genenames.org), Gene Ontology database (http://geneontology.org), GENCODE v27 (https://www.gencodegenes.org)

Human research participants

Policy information about studies involving human research participants and Sex and Gender in Research.

Reporting on sex and gender	NA
Population characteristics	NA
Recruitment	NA
Ethics oversight	NA

Note that full information on the approval of the study protocol must also be provided in the manuscript.

Field-specific reporting

Please select the one below that is the best fit for your research. If you are not sure, read the appropriate sections before making your selection.

☒ Life sciences ☐ Behavioural & social sciences ☐ Ecological, evolutionary & environmental sciences

For a reference copy of the document with all sections, see nature.com/documents/nr-reporting-summary-flat.pdf

Life sciences study design

All studies must disclose on these points even when the disclosure is negative.

Sample size	Sample sizes were chosen considering the feasibility of experimental manipulation while maintaining biological, technical and experimental replication. We used sample sizes that are very consistent with those used in our field that balance the required statistical power. For example, regarding number of lines used all previous publications addressing viral infection of brain organoids use either one, e.g.: https://www.science.org/doi/epdf/10.1126/science.aaf6116 and https://www.science.org/doi/10.1126/sciadv.aay8828<br>Or with two iPSC lines, e.g. https://www.embopress.org/doi/full/10.15252/embj.2020106230 and https://journals.plos.org/plosbiology/article?id=10.1371/journal.pbio.3001845
Data exclusions	No data were excluded
Replication	All findings were replicated and all replicates are shown either in the figures or in the associated raw data. Multiple samples were analyzed by independent approaches (ie. histological, molecular analysis, functional analysis) and all experiments were performed more than once, majority cases three times as indicated in figure legends.
Randomization	Brain organoids were randomly selected for either infection or no infection.
Blinding	Most eperiments were not performed blindly. When applicable, this is indicated in the Methods section.

Reporting for specific materials, systems and methods

We require information from authors about some types of materials, experimental systems and methods used in many studies. Here, indicate whether each material, system or method listed is relevant to your study. If you are not sure if a list item applies to your research, read the appropriate section before selecting a response.

Materials & experimental systems

n/a	Involved in the study
☐	☒ Antibodies
☐	☒ Eukaryotic cell lines
☒	☐ Palaeontology and archaeology
☒	☐ Animals and other organisms
☒	☐ Clinical data
☒	☐ Dual use research of concern

Methods

n/a	Involved in the study
☒	☐ ChIP-seq
☒	☐ Flow cytometry
☒	☐ MRI-based neuroimaging

Antibodies

Antibodies used

Immunostaining: Primary antibodies: STMN2 (Bio-Techne, NBP1-49461, 1:1000), SOX2 (Merck, AB5603, 1:1000), PAX6 (Biolegends 901301, 1:1000), ZO-1 (Invitrogen 339100, 1:1000), NeuN (Sigma, MAB377, 1:500), MAP2 Sigma, MAB3418A5, 1:1000), SYN1 (Abcam ab8, 1:1000), TUJ1 (Sigma T2200, 1:1000), GFAP (Milipore/Merck, 1:1000), TBR1 (Abcam, ab183032, 1:1000), GFP (Abcam, ab13970, 1:1000), Phosphor-Vim (pVIM) (Biozol Diagnostics, MBL-D076-3, 1:2000), SATB2 (Abcam, ab34735, 1:1000), C-Caspase3(Cell signaling 9661, 1:500), ICP0 (Santa Cruz SC53070, 1:500). Secondary antibodies: Alexa 488 anti chicken (Abcam 150169, 1:500), Alexa 568 anti mouse (Abcam15747, 1:500), Alexa 649 anti mouse (Abcam150115, 1:500), Alexa 568 anti rabbit (Abcam175473, 1:500), Alexa 649 anti rabbit (Abcam175473, ThermoFisherA21447, 1:500), Alexa 649 anti goat (ThermoFisherA21447, 1:500)
WB: Primary antibodies: AKT (Cell Signaling 9272, 1:1000), pAKT (Cell Signaling 9277S, 1:1000), SYN1 (Abcam, ab8, 1:1000), TRAF6 (Santa Cruz 7221, 1:1000), ICP0 (Santa Cruz SC53070, 1:800), GFP (Merck G1546, dilution 1:1000), pERK1/2 (Cell signaling 9102, dilution WB: 1:1000), HOMER1 (synaptic Systems, 160011, 1:1000), phosopho-p65 (p-p65) (Cell Signaling, 3033T, dilution WB: 1:2000), GAPDH (Sigma, G8795, 1:2000) Secondary antibodies: Goat anti-Mouse IgG (H L) HRP (ThermoFisher Scientific 31430, 1:8000), Polyclonal Goat Anti rabbit HRP, Dako P0448, 1:5000),

Validation

All antibodies used in this study are commercial, and validation information is available on the manufacturer's website.

Eukaryotic cell lines

Policy information about cell lines and Sex and Gender in Research

Cell line source(s)

XM001 hiPSC cells were kindly provided by the Heiko Lickert (Technical University Munich) under a Material Transfer Agreement with our institution. Gibco Human 18 Episomal iPS line 1E6 (Gibco/ThermoFischer scientific, cat#A18944, lot #2036936).

Authentication

XM001 and Gibco Human 18 are maintained and validated by the Stem Cells core facility and Organoid platform at our institution. Quality checks were done using karyotyping, cell identity (STR ANALYSIS), undifferentiated phenotype, differentiation potential, reprograming vector clearance, sterility (mycoplasma, yeast, bacteria).

Mycoplasma contamination

All batches and lines were regularly tested for mycoplasma contamination. All cell lines were tested negative (data available upon request).

Commonly misidentified lines
(See ICLAC register)

No commonly misidentified lines were used in this study.
