# Supplementary figures and images for: Modelling viral encephalitis caused by herpes simplex virus 1 infection in cerebral organoids
Source: Nat Microbiol. 2023 Jun 22;8(7):1252–66. doi: 10.1038/s41564-023-01405-y (PMC10322700; doi:10.1038/s41564-023-01405-y)

Source data Fig 4c

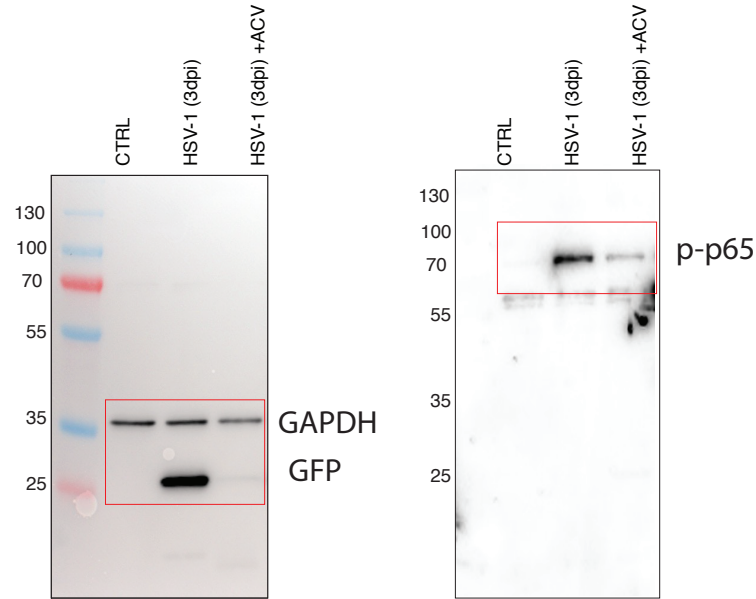

Supplement: Source Data Fig. 4 — Unprocessed western blots. [file 41564_2023_1405_MOESM7_ESM.pdf]

Source data Fig 6

a

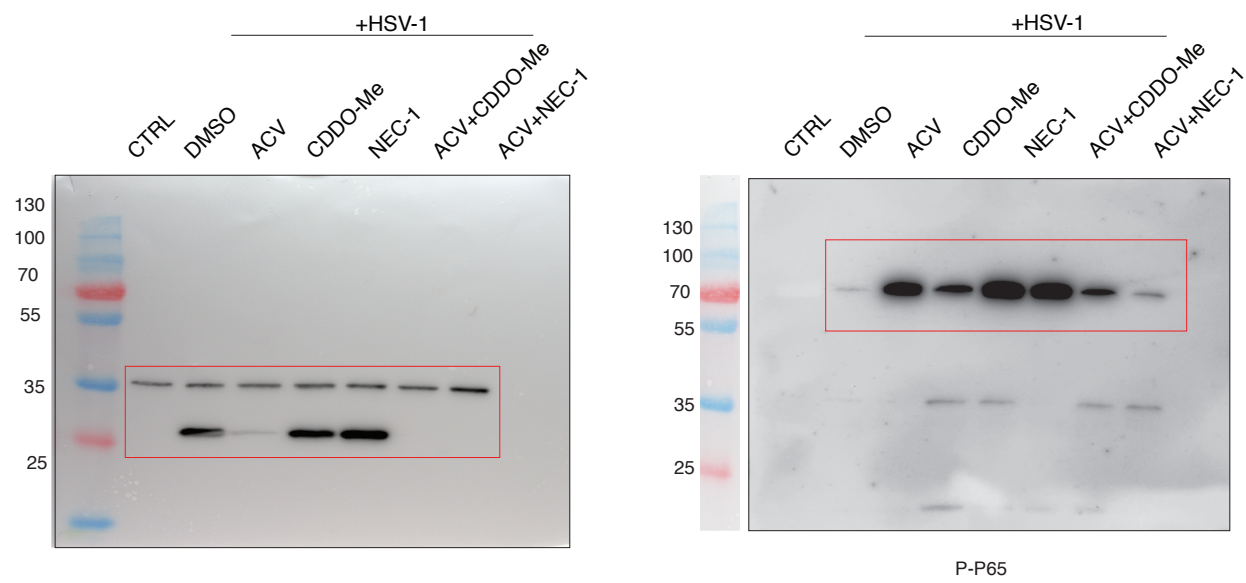

c

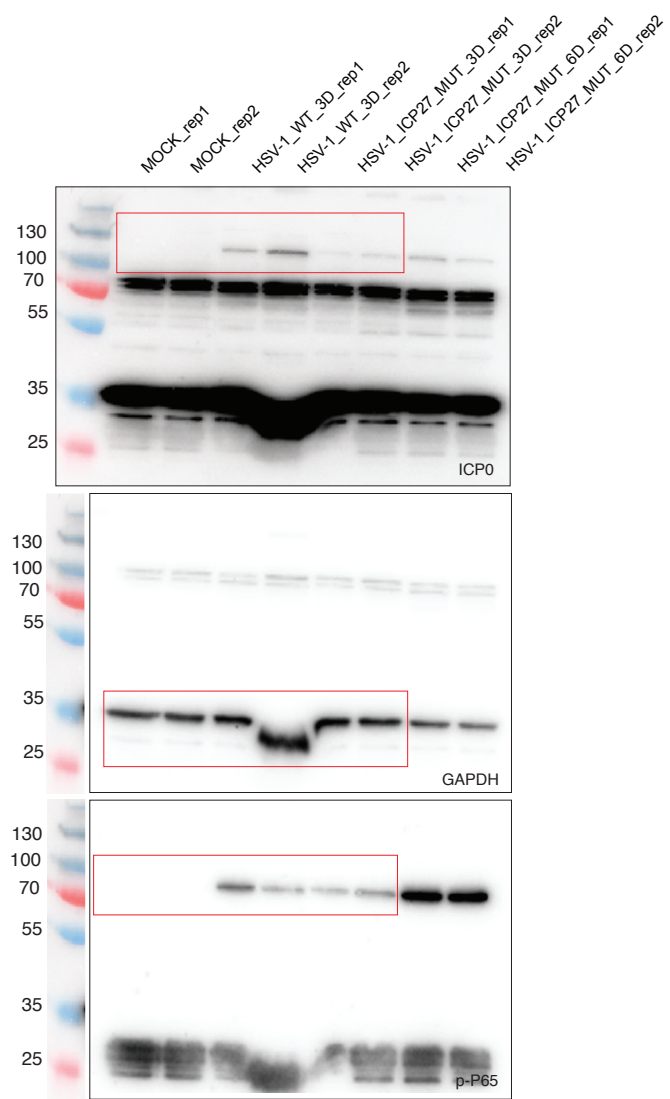

Supplement: Source Data Fig. 6 — Unprocessed western blots. [file 41564_2023_1405_MOESM9_ESM.pdf]

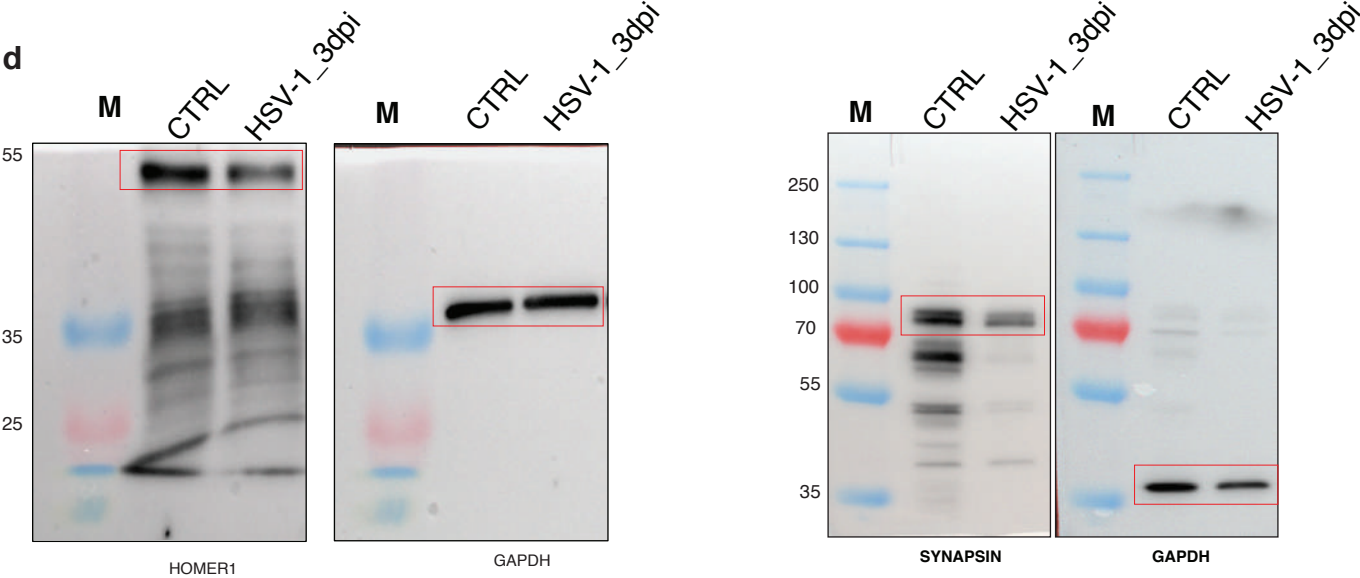

Supplement: Source Data Extended Data Fig. 2 — Unprocessed western blots. [file 41564_2023_1405_MOESM11_ESM.pdf]

i

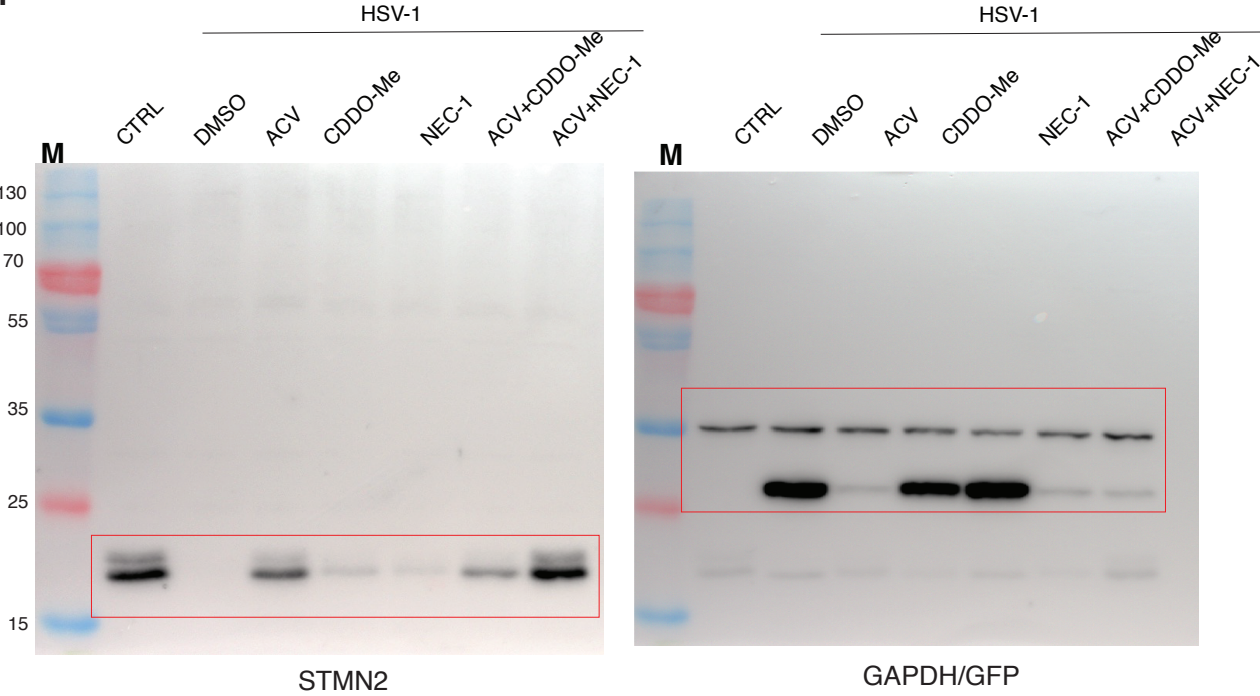

Supplement: Source Data Extended Data Fig. 9 — Unprocessed western blots. [file 41564_2023_1405_MOESM14_ESM.pdf]

**Source data Extended data Fig.10** Uncropped scans of blots

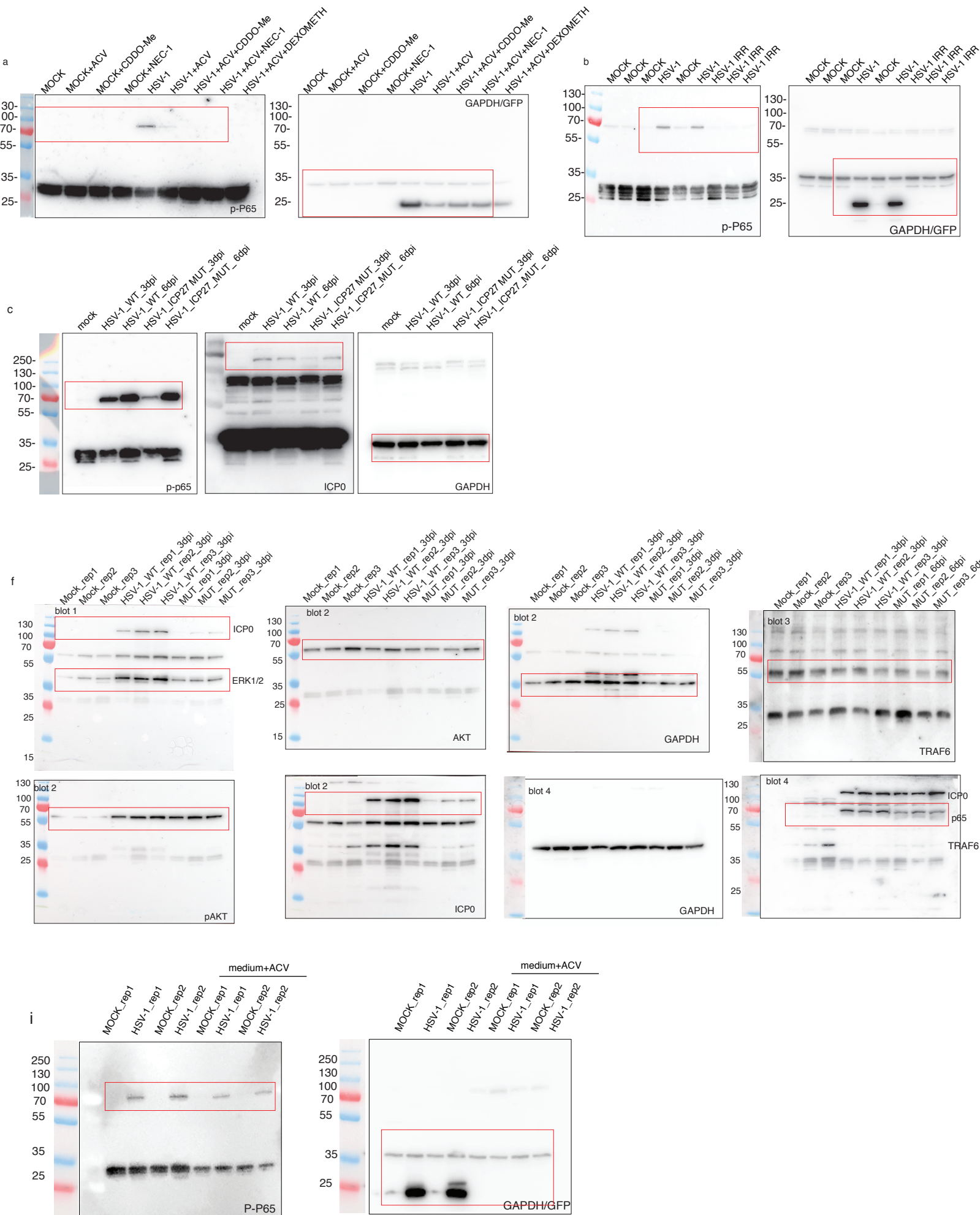

Supplement: Source Data Extended Data Fig. 10 — Unprocessed western blots. [file 41564_2023_1405_MOESM16_ESM.pdf]
