# Supplementary material for: Modelling viral encephalitis caused by herpes simplex virus 1 infection in cerebral organoids
Source: Nat Microbiol. 2023 Jun 22;8(7):1252–66. doi: 10.1038/s41564-023-01405-y (PMC10322700; doi:10.1038/s41564-023-01405-y)
Supplement: Supplementary file 3 — Peer Review File [file 41564_2023_1405_MOESM3_ESM.doc]

Peer Review Information

**Journal: Nature Microbiology**

**Manuscript Title:** Modelling viral encephalitis caused by herpes simplex virus 1 infection in cerebral organoids

**Corresponding author name(s): Professor Nikolaus Rajewsky, Dr Agnieszka Rybak-Wolf**

**Decision Letter, initial version:**

| **Message:** | 10th October 2022  Dear Professor Rajewsky,  Thank you for your patience while your manuscript "Modeling herpes simplex virus 1 infection in cerebral organoids reveals new potential therapeutic approaches for viral encephalitis" was under peer review at Nature Microbiology. It has now been seen by our referees, whose expertise and comments you will find at the end of this email. In the light of their advice, we have decided that we cannot offer to publish your manuscript in Nature Microbiology.  From the reports, you will see that while they find your work of some potential interest, the referees raise concerns about the advance your findings represent over earlier work and the descriptive nature of the findings that are sufficiently important as to preclude publication of your work in Nature Microbiology.  I am sorry that we cannot be more positive on this occasion, but hope that you find the referees' comments helpful when preparing your paper for resubmission elsewhere.  Yours sincerely,  [redcated]   ******************* Reviewer Expertise:  Referee #1: Organoid models for viral disease Referee #2: HSV-1 infection of the brain Referee #3: Herpesvirology  Reviewers Comments:  Reviewer #1 (Remarks to the Author):  In this manuscript, Wolf, Wyler, and Pentimali et al establish cerebral organoids as models for HSV-1 infection. The author apply single cell RNASeq of infected organoids to define the host pathways induces by HSV-1 infection. In addition, they show that while treatment with acyclovir inhibited viral replication, as expected, it was unable to control the induction of signaling pathways such as the TNF pathway, indicating that antiviral therapy alone may not be sufficient to prevent HSV-1-mediated neuronal damage. The manuscript is overall well-done and the most of the conclusions justified by the data provided. However, the lack of data to define the pathway(s) by which inflammatory pathways are activated in the absence of replication and the lack of depth in the studies regarding the combinatorial approaches suggested (e.g., in vivo) detract from the other aspects of the manuscript. My specific comments are below.  1. Cerebral organoids vary by size greatly and it is unclear how the authors calculate MOI based on "estimated number of cells at the surface of the organoid". More details are required regarding the calculation of MOI, which may be quite difficult given the organoid system.  2. An important control is missing as a ACV alone condition would be important to ensure that the pathways being induced are not associated with this treatment.  3. Figure 3D is not mentioned in the text.  4. Single cell reconstruction of cerebral organoids has been published by others (Kanton et al, Fiorenzano et al, Camp et al) and the authors should provide a more detailed discussion regarding these studies.   Reviewer #2 (Remarks to the Author):  Review „Modeling herpes simplex virus 1 infection in cerebral organoids reveals new potential therapeutic approaches for viral encephalitis”  The manuscript by Rybak-Wolf et al. describes human stem cell derived cerebral organoids demonstrating morphological as well as functional tissue integrity changes upon HSV-1 infection. HSV-1 is one of the most common pathogens leading to fatal human encephalitis. The pathogenesis is largely unclear. However, treatment with acyclovir improves outcomes of the disease, but patients still suffer from severe neurological sequelae. The authors of the present study particularly figured out that in contrast to antiviral therapy with acyclovir alone, tissue damage of organoids is minimal or even absent with combined anti-viral and anti-inflammatory therapy. The results of this study are presented in a comprehensive and comprehensible manner, although the progress in knowledge regarding better therapy options is not entirely new (please see Boivin et al. 2013). Boivin and colleagues used a murine mouse model of herpes simplex virus encephalitis and made similar conclusions on combined therapy for HSE. Nevertheless, I recommend to accept the manuscript for publication with minor points which have to be modified as listed below.  Lines 59-61: It is well-known that animal models for HSE largely lack analogies to human disease. Please describe the most important weaknesses of animal models in more detail and emphasize the advantage of your organoid model compared to murine models for instance. Line 97: The organoids revealed dorsal forebrain region. Is it morphologically comparable to the temporal/frontal lobe which is commonly affected in HSE? Line 118: I would prefer to designate the full name of the virus strain used. Lines 152-154: Figure 2E indicates immunohistochemistry/immunofluorescence. How did the authors quantify the signals? Please clarify/modify the corresponding figure (as indicated in the material and methods section). Lines 155-161: Neuroepithelial identity changes were analyzed by ZO-1 immunostaining. To underline this finding, is there another useful cell polarity marker? Line 169: The term “affects cellular identities” is confusing. Would it be better to use “cellular composition” instead of “cellular identities”? Lines 199: Please specify what “highly infected” means. Lines 213-215: How long are cells “highly infected”? Did the authors look beyond 3 dpi? Lines 264-265: Please specify “tissue and cellular damage”. For the reader it is not clear which morphological changes or lesions are meant: necrosis, apoptosis, etc. Line 425: Please modify: “For immunostaining, … and fixed in 4%...” Line 435: Please modify: “…overnight, and washed three times…” Line 436: Please modify: “…at RT for 2h, stained…” Lines 432-437: Please shorten this long sentence. Figure 6: Please change figure caption: “B” is designated as “G”.    Reviewer #3 (Remarks to the Author):  Mechanisms driving HSV-1 infection of the human brain and associated pathologies are poorly understood. Given the fact that HSV-1 causes encephalitis and the possibility that it may affect neurodegeneration in the CNS, multiple attempts have been made to model the consequences of HSV-1 infection via human iPSC or ESC 3D cultures often termed as brain organoids or in this case, cerebral organoids. Past studies (summarized in Depla et al., Viruses, 2022; Cairns et al., 2020, Sci Adv) and a 2021 bioRxiv paper by the authors (Rybak-Wolf et al.) elegantly demonstrate the use of such 3D brain organoid in HSV-1 infection and infection-associated neuronal loss. A major strength of the manuscript is that it is well presented and well written. A few major weaknesses however significantly reduce enthusiasm. A major problem is that the findings are mostly descriptive, lack proper controls and often fail to go too much beyond what is already known in the field. In other words, it lacks depth and the novelty is incremental. Some other issues are listed below. 1. Fig. 1, very little new information is gained from this figure. Similar information can also be found in their 2021 bioRxiv paper. 2. Fig. 2, a large portion of the results shown here have already been described elsewhere using 3D brain organoids (please see the references above). Calcium imaging data lack evidence whether active viral replication is required or not. Long time ago Cheshenko et al. (JCB, 2003) showed that Ca signaling is needed during viral entry. Use of a replication deficient virus can provide more knowledge on this phenomenon and better explain the observations made in this figure. 3. Fig. 3, did the authors use sucrose density gradient purified virus here and in the previous figure? If infected cell lysates were used (most common), then many inflammatory/toxic substances present in the lysates can also cause the observations made here. Their control is no viral infection; a true scientific control would be lysates from noninfected cells if the infected cell lysates were used for the infection. Fig 3D, ACV alone control is missing. 4. Figs. 4/5, It is gratifying that TNFa signaling and the inability of ACV to reduce its activity are also recapitulated well in this model. However, this knowledge already exists from previous animal studies and human clinical observations. The data shown here are descriptive and provide very little new knowledge. Again, ACV alone control is missing, which makes this study less rigorous. 5. Fig. 5C/D GFP intensity for the treated organoids never reaches the control levels. Still the data in Fig.6 claims near complete inhibition of the infection. How to truly reconcile the two apparently contrasting results? If GFP intensity has no real meaning, then why to show the data? How deep into the tissue the drugs can penetrate? What would be the mechanism? Since they are basically reproducing the prior knowledge from other model systems, the authors should deliver the neurovirulent Y34.5 protein of HSV-1 to demonstrate a viral determinant of the cytopathic/inflammatory effects observed in their cerebral organoids. This way the study will demonstrate a molecular mechanism by which HSV-1 causes damage in the organoids and be less descriptive. Alternatively, the non-neurovirulent HSV-1 Y34.5 deletion virus should be compared with the wildtype virus.      *******************   ** I suggest that you consider revising and transferring a suitable revised version to Nature Communications. To transfer your manuscript there, please use our [redacted] You will not have to re-supply manuscript metadata and files, unless you wish to make modifications, but please note that this link can only be used once and remains active until used. For more information, please see our <a href="http://www.nature.com/authors/author_resources/transfer_manuscripts.html?WT.mc_id=EMI_NPG_1511_AUTHORTRANSF&WT.ec_id=AUTHOR">manuscript transfer FAQ</a> page.  Note that any decision to opt in to In Review at the original journal is not sent to the receiving journal on transfer. You can opt in to <i><a href="https://www.nature.com/nature-portfolio/for-authors/in-review">In Review</a></i> at receiving journals that support this service by choosing to modify your manuscript on transfer. In Review is available for primary research manuscript types only. |
| --- | --- |

**Author Rebuttal to Initial comments**

# Point-by-point response and revision plan

Here we reply to all the reviewers’ points, report two additional requested datasets and analyses, and propose four additional experiments to address the specific points.

**Reviewer #1 (Remarks to the Author):**

*In this manuscript, Wolf, Wyler, and Pentimali et al establish cerebral organoids as models for HSV-1 infection. The author apply single cell RNASeq of infected organoids to define the host pathways induces by HSV-1 infection. In addition, they show that while treatment with acyclovir inhibited viral replication, as expected, it was unable to control the induction of signaling pathways such as the TNF pathway, indicating that antiviral therapy alone may not be sufficient to prevent HSV-1-mediated neuronal damage. The manuscript is overall well- done and the most of the conclusions justified by the data provided. However, the lack of data to define the pathway(s) by which inflammatory pathways are activated in the absence of replication and the lack of depth in the studies regarding the combinatorial approaches suggested (e.g., in vivo) detract from the other aspects of the manuscript. My specific comments are below.*

1. *Cerebral organoids vary by size greatly and it is unclear how the authors calculate MOI based on "estimated number of cells at the surface of the organoid". More details are required regarding the calculation of MOI, which may be quite difficult given the organoid system.*

We have calculated the surface cell number based on the total number of cells and the organoid size. This of course was only an estimation. We can also rephrase it and simply say that we used certain number of viral particles per organoid.

1. *An important control is missing as a ACV alone condition would be important to ensure that the pathways being induced are not associated with this treatment.*

We have previously performed single-cell sequencing of ACV-only-treated organoids and we will include this control in the manuscript, and we will repeat all the immunostaining and western blot analyses with such control as well.

However, based on the single-cell data, we can conclude that there is no significant change in cell type composition introduced by ACV treatment alone - as shown below (UMAP) and no activation of TNF pathway observed (box plot).

2. *Figure 3D is not mentioned in the text.*

Will be corrected.

3. *Single cell reconstruction of cerebral organoids has been published by others (Kanton et al, Fiorenzano et al, Camp et al) and the authors should provide a more detailed discussion regarding these studies.*

We in fact used these papers as an important source to define the cell-types in our organoid model, however since our paper is focused on the molecular consequences of the infection, we preferred to not go into excessive detail in this regard. We can however, more clearly state in the paper that these papers were used as a reference and add some comparisons in the supplement.

***Reviewer #2 (Remarks to the Author):***

*The manuscript by Rybak-Wolf et al. describes human stem cell derived cerebral organoids demonstrating morphological as well as functional tissue integrity changes upon HSV-1 infection. HSV-1 is one of the most common pathogens leading to fatal human encephalitis. The pathogenesis is largely unclear. However, treatment with acyclovir improves outcomes of the disease, but patients still suffer from severe neurological sequelae. The authors of the present study particularly figured out that in contrast to antiviral therapy with acyclovir alone, tissue damage of organoids is minimal or even absent with combined anti-viral and anti- inflammatory therapy.*

*The results of this study are presented in a comprehensive and comprehensible manner, although the progress in knowledge regarding better therapy options is not entirely new (please see Boivin et al. 2013). Boivin and colleagues used a murine mouse model of herpes simplex virus encephalitis and made similar conclusions on combined therapy for HSE. Nevertheless, I recommend to accept the manuscript for publication with minor points which have to be modified as listed below.*

We cited Boivin et al. in the discussion (reference 46): “Our data suggest that, at a stage of acute infection, a combination of antiviral and anti-inflammatory agents (necrostatin-1, bardoxolone methyl) significantly reduced organoid damage, strongly suggesting that a combinatorial therapy targeting viral replication and an overzealous inflammatory response may be beneficial in a clinical setting. It has been previously shown in a mouse model that

HSV-1 induces expression of TNF-α (44), and that co-treatment with etanercept (an anti-TNF- α antibody) or glucocorticoids increases the survival rate of HSV-1-infected mice compared to these only treated with antiviral drugs (45,46).” We want to stress here that despite this the reviewer recommended to accept the manuscript with minor revision, and that besides the finding of anti-TNF treatment in mice being in agreement with our findings, our work presents an unbiased analysis of a human model and comes to the conclusion that leveraging the TNF pathway in a therapeutic setting might not only ameliorate the outcome in terms of survival as observed in mice (which is already high for ACV in HSE patients), but in terms of impacting on specific molecular, cellular and tissue-level consequences of the infection which are not rescued with ACV.

*Lines 59-61: It is well-known that animal models for HSE largely lack analogies to human disease. Please describe the most important weaknesses of animal models in more detail and emphasize the advantage of your organoid model compared to murine models for instance.*

Several mouse models with different age, genetic background, inoculation routes, and virus strains of variable neurovirulence have been developed to investigate the pathogenesis of HSV1 infections in vivo (Mancini et al., 2018). So far, mostly intranasal or intracerebral infection models have been used to study HSE in mice and the infection pattern observed was more diffuse than that observed in HSE patients, indicating that the mechanism of virus spreading can be different in human. For example, the virus did not spread to the temporal and frontal lobes, which are the typical locations in HSE in humans.

HSV-1 is a human specific virus, therefore in order to precisely model its pathogenesis, host response and to develop preventive and therapeutic treatments we need to use human models. iPSC derived organoids recapitulate partially the complexity and many characteristics of in vivo disease. For example, some aspects such as pre-long infections or latency are difficult to model in 2D culture, but were possible in organoids. (D’Aiuto et al., 2019)

Organoids are emerging as the future preclinical tools for disease modeling and drug testing. Although still relatively simple models, they are easier to handle and genetically manipulate compared to mice and reflect closer human genetics. Therefore, they serve as meaningful translational tool for testing drugs for clinical applications.

We can add these considerations (in a concise way) in the discussion of our manuscript.

*Line 97: The organoids revealed dorsal forebrain region. Is it morphologically comparable to the temporal/frontal lobe which is commonly affected in HSE?*

With the unguided cerebral organoid protocol that we implemented, we predominantly model the dorsal forebrain region, but also subsets of subcortical neuronal types as shown in Figure

1. Since the organoid model reflects only an early stage of brain development it is difficult to compare it to adult temporal/frontal lobe. It is rather similar to the earlier stage of diencephalon and telencephalon, from which these regions originate. Of note, brain organoids are still the most complex human-like brain tissue models currently available for in vitro studies.

*Line 118: I would prefer to designate the full name of the virus strain used.*

*>>> Lines 152-154: Figure 2E indicates immunohistochemistry/immunofluorescence. How did the authors quantify the signals? Please clarify/modify the corresponding figure (as indicated in the material and methods section).*

*>>> Lines 155-161: Neuroepithelial identity changes were analyzed by ZO-1 immunostaining. To underline this finding, is there another useful cell polarity marker?*

*>>> Line 169: The term “affects cellular identities” is confusing. Would it be better to use “cellular composition” instead of “cellular identities”?*

*>>> Lines 199: Please specify what “highly infected” means.*

*>>> Lines 213-215: How long are cells “highly infected”? Did the authors look beyond 3 dpi?*

*>>> Lines 264-265: Please specify “tissue and cellular damage”. For the reader it is not clear which morphological changes or lesions are meant: necrosis, apoptosis, etc.*

*>>> Line 425: Please modify: “For immunostaining, … and fixed in 4%...”*

*>>> Line 435: Please modify: “…overnight, and washed three times…”*

*>>> Line 436: Please modify: “…at RT for 2h, stained…”*

*>>> Lines 432-437: Please shorten this long sentence.*

*>>> Figure 6: Please change figure caption: “B” is designated as “G”.* We will correct these sentences and add all the relevant information. ***Reviewer #3 (Remarks to the Author):***

*Mechanisms driving HSV-1 infection of the human brain and associated pathologies are poorly understood. Given the fact that HSV-1 causes encephalitis and the possibility that it may affect neurodegeneration in the CNS, multiple attempts have been made to model the consequences of HSV-1 infection via human iPSC or ESC 3D cultures often termed as brain organoids or in this case, cerebral organoids. Past studies (summarized in Depla et al., Viruses, 2022; Cairns et al., 2020, Sci Adv) and a 2021 bioRxiv paper by the authors (Rybak-Wolf et al.) elegantly demonstrate the use of such 3D brain organoid in HSV-1 infection and infection-associated neuronal loss. A major strength of the manuscript is that it is well presented and well written. A few major weaknesses however significantly reduce enthusiasm. A major problem is that the findings are mostly descriptive, lack proper controls and often fail to go too much beyond what is already known in the field. In other words, it lacks depth and the novelty is incremental. Some other issues are listed below.*

1. *Fig. 1, very little new information is gained from this figure. Similar information can also be found in their 2021 bioRxiv paper.*

Our current manuscript originates from that preprint, which was presenting a rather general description of multiple modalities of molecular characterization of HSV1 infection of brain organoids. In a subsequent effort, we focused on the TNF pathway which was the strongest signature observed from these data, and collected our additional results in the manuscript submitted here. For this reason, figure 1 of this manuscript presents similar (but more thorough and clearer) results than that in our preprint, as this manuscript is a sort of “version 2” of that preprint. We will not submit this bioRxiv preprint but replace it with the current version.

1. *Fig. 2, a large portion of the results shown here have already been described elsewhere using 3D brain organoids (please see the references above). Calcium imaging data lack evidence whether active viral replication is required or not. Long time ago Cheshenko et al. (JCB, 2003) showed that Ca signaling is needed during viral entry. Use of a replication deficient virus can provide more knowledge on this phenomenon and better explain the observations made in this figure.*

The purpose of calcium imaging experiment was to demonstrate the loss of coordinated neuronal activity upon HSV-1 infection.

With our experimental setup, we model the current clinical scenario, where the ACV treatment is implemented, when the virus spreads already through the brain tissue and when the viral replication with ACV occurs in the management of HSE patients. We therefore do not expect a similar overzealous inflammatory response in case viral replication is inhibited from the start of the infection, as it would happen with a replication deficient virus. As a matter of fact, in our 2021 preprint we have used the ICP27 knockout virus (replication deficient), and did not observe strong morphological changes related to neuroepithelium disintegration, and only partial depletion of proliferating radial glia cells (Rybak-Wolf et al., bioRxiv 2021, see below image from that preprint).

2. *Fig. 3, did the authors use sucrose density gradient purified virus here and in the previous figure? If infected cell lysates were used (most common), then many inflammatory/toxic substances present in the lysates can also cause the observations made here. Their control is no viral infection; a true scientific control would be lysates from noninfected cells if the infected cell lysates were used for the infection. Fig 3D, ACV alone control is missing.*

We performed the experiments with lysates as well as with concentrated purified virus, observing similar effects. Also, the volume of added lysate was never exceeding 5% of the total culture medium. However, to address the reviewer’s comment we can include an additional non-infected cells lysate as a control for immunostainings/Western analyses.

3. *Figs. 4/5, It is gratifying that TNFa signaling and the inability of ACV to reduce its activity are also recapitulated well in this model. However, this knowledge already exists from previous animal studies and human clinical observations. The data shown here are descriptive and provide very little new knowledge. Again, ACV alone control is missing, which makes this study less rigorous.*

To our knowledge, there are no clinical data pointing towards TNFalpha activation after ACV treatment, only clinical observation related to neurological sequelae. Also, in Bovin et al., 2013, only a survival analysis was performed in mice, without any molecular characterization.

It is true that it was shown previously that TNFalpha is activated after HSV-1 infection, but there is no paper showing that this activation persists after ACV treatment, nor that targeting this pathway ameliorates molecular, cellular or tissue-level effects of the infection. In our paper, we could clearly demonstrate using scRNAseq analysis, immunostaining and western blotting that not only TNF alpha activation remains after ACV treatment, but also the cellular and tissue changes (neuroepithelial integrity, STMN2 expression) associated with viral infection are only partially rescued by current treatments while they are better resolved by complementary treatments.

4. *Fig. 5C/D GFP intensity for the treated organoids never reaches the control levels. Still the data in Fig.6 claims near complete inhibition of the infection. How to truly reconcile the two apparently contrasting results? If GFP intensity has no real meaning, then why to show the data?*

In all the data presented in our study with the ACV treatment, we observe a significant downregulation of the GFP expression. When comparing immunofluorescence (IF) and Western blots, it is important to note that IF images are usually centered on the external part of the organoids, which is more accessible and better infected, while Westerns are performed on lysates from whole organoids. Besides this difference, which may introduce a systematic bias between the two readouts, we must point out the following:

- GFP does not reach control levels even in the Western blots in Fig. 6;
- We provide immunofluorescence quantifications in Fig. S5 for low and high MOI; in these two cases, as we would expect, the viral GFP signal is differently impacted by the treatment: with low MOI we can much better control viral replication than with high MOI;
- In Fig. 6, we show Western blots from experiments performed with a similar MOI than that of the “low MOI” treatment in Fig. S5;
- To quantify whether the Western and Immunostainings are in good agreement (and therefore that the GFP signal is indeed meaningful), we show below a scatterplot of the average GFP intensity from the two readouts (Western blots with low MOI and immunofluorescence with low and high MOI) in each condition: the low MOI IF GFP signal is indeed extremely well correlated to the Western GFP signal (Pearson = 0.979).

To further address this comment, we can better discuss this in the results section of the manuscript and show this analysis in the supplement.

(1) Furthermore, we can measure GFP and expression of other viral genes by qRT-PCR if necessary in order to convince people that GFP is a good proxy for viral replication.

*How deep into the tissue the drugs can penetrate? What would be the mechanism?*

Drug penetration is an issue that we might have difficulties to fully resolve. There are variants of ACV that are thought to have better permeability, but from our data we can conclude that drug penetration is sufficient to stop viral replication, which is not restricted to the organoids surface.

*Since they are basically reproducing the prior knowledge from other model systems, the authors should deliver the neurovirulent Y34.5 protein of HSV-1 to demonstrate a viral determinant of the cytopathic/inflammatory effects observed in their cerebral organoids. This way the study will demonstrate a molecular mechanism by which HSV-1 causes damage in the organoids and be less descriptive. Alternatively, the non-neurovirulent HSV-1 Y34.5 deletion virus should be compared with the wildtype virus.*

The claim of our paper is that the exaggeration of the initial inflammatory response induced by viral infection damages organoids at different levels. If the virus is not replicating, there should not be induction of inflammation and tissue damage. This is also in line with for example this previous study performed in mouse models: Orvedahl et al., Cell Host Microbe (2007), where the knockout of ICP34.5 reduced encephalitis in mice, indicating that a non-replicating virus has less inflammatory effects.

To prove that ICP34.5 virus has also less inflammatory effects in our system, we propose to:

(1) Repeat the experiments with an ICP34.5 knockout virus along with the ICP27 knockout virus, and check for inflammatory response (activation of p65 phosphorylation). We will use BAC-derived knockout viruses in order to ensure identical genetic backgrounds of knockout and wildtype virus.

Furthermore, to support our claim, we can perform the following experiments:

(2) Incubate the organoids with the medium/lysate from infected organoids after inactivation of viral replication with ACV and then perform p-p65 analysis to demonstrate if the observed activation of the TNF pathway is driven by cytokines produced during viral infection;
(3) Perform the analysis of the cytokines produced during viral infection and after the treatment, to narrow down which specific cellular responses are involved.

**Decision Letter, first revision:**

| **Message:** | 18th November 2022  Dear Professor Rajewsky,  Thank you for your letter asking us to reconsider our decision on your Article entitled "Modeling herpes simplex virus 1 infection in cerebral organoids reveals new potential therapeutic approaches for viral encephalitis". After careful consideration we have decided that we would be willing to consider a revised version of your manuscript.  Please revise your manuscript according to the points outlined in your rebuttal including the listed experiments.  Along with your revised manuscript, you should also submit a separate point-by-point response to all of the concerns raised by the referees, in each case describing what changes have been made to the manuscript or, alternatively, if no action has been taken, providing a compelling argument for why that is the case.  Regarding the issue raised by reviewer #3: "How deep into the tissue the drugs can penetrate? What would be the mechanism?", please indicate in your point-by-point response to the reviewer exactly, which data in your study supports sufficient drug penetration for fully stopping viral replication.  If we feel that a substantial attempt has been made to address the referees' comments, this response will be sent back to the referees - along with the revised manuscript - so that they can judge whether their concerns have been addressed satisfactorily or otherwise.  I should stress, however, that we would be reluctant to trouble our referees again unless we thought that their comments had been addressed in full.  When revising your paper:  - ensure it complies with our format requirements for Letters as set out in our guide to authors at www.nature.com/nmicrobiol/authors/index.html  - state in a cover note the length of the text, methods and legends; the number of references and the number of display items.  Please ensure that all correspondence is marked with your Nature Microbiology reference number in the subject line.  Please use the following link to submit your revised manuscript:  https://mts-nmicrobiol.nature.com/cgi-bin/main.plex?el=A5Cg7UQ3A1RSS4J2A9ftdAcn25xll4VnMv62eI2sXxQZ  We hope to receive your revised paper within four weeks. If you cannot send it within this time, please let us know so that we can close your file. In this event, we will still be happy to reconsider your paper at a later date so long as nothing similar has been accepted for publication at Nature Microbiology or published elsewhere in the meantime. Should you miss the four-week deadline and your paper is eventually published, the received date will be that of the revised, not the original, version.  I would appreciate it if you could tell me if you think you will be able to submit a revised manuscript, and also the likely timescale.  I look forward to hearing from you soon.  Yours sincerely, |
| --- | --- |

**Author Rebuttal, first revision:**

Dear Reviewers,

We thank you very much for your thoughtful evaluation of our work. We went carefully through all the point raised and we are happy to read that:

- The manuscript is overall well-done and the most of the conclusions justified by the data provided.
- The results of this study are presented in a comprehensive and comprehensible manner.
- I recommend to accept the manuscript for publication with minor points.

It is our understanding the main concerns regarding the suitability of our paper for publication in Nature Microbiology lie in the lack of novelty of our findings, specifically:

- “[…] the progress in knowledge regarding better therapy options is not entirely new (please see Boivin et al. 2013).”
- “Boivin and colleagues used a murine mouse model of herpes simplex virus encephalitis and made similar conclusions on combined therapy for HSE.”
- “A major problem is that the findings are mostly descriptive, lack proper controls and often fail to go too much beyond what is already known in the field.”

*We provide below* ***a summary of the major experimental data added*** *to the paper in response to the referees’ comments. We then provide a* ***detailed and exhaustive point-by-point response****. However, we would first like to comment on* ***the novelty*** *and medical significance of our work (which builds on Boivin et al):*

1) ***Generation of the first human model of ACV-treated HSE****. Acyclovir (ACV) revolutionized the treatment of patients with Herpes induces Encephalitis (HSE), increasing survival from ~30% to*

~90% (Riera-Mestre *et al*. 2009). Nevertheless, 70% of the surviving patients retain neurological damage. This shows a great clinical need, namely not only to ensure survival but also to reduce long-term consequences of the infection. Unfortunately, this need is very difficult to address since live brain tissues from these patients obviously cannot be procured. For these reasons, models are needed to study the molecular mechanisms involved. Mouse models are problematic because, as referee 2 rightly points out, as “*It is well-known that animal models for HSE largely lack analogies to human disease*”. Here, we present the **first human model for ACV treatment** in a neural tissue, and demonstrate that our infected organoids show molecular and cellular phenotypes which are **consistent with HSE pathology in patients**. We also show that co-treatment with anti-inflammatory drugs prevents these phenotypes, providing evidence that co-treatment is relevant for HSE patients.

2) *In our system, comprehensive single-cell analyses show that* **TNF */NFkB is likely the only pathway by which the clinical outcome can be improved****. This is, we think, a very important result as it suggests that co-treatment with anti-inflammatory drugs that act on that pathway can potentially prevent neuronal damage caused by HSV despite treatment with ACV.*
3) ***On the molecular and tissue level, we show that HSV-1 causes phosphorylation of NFkB subunit p65 and tissue damage (STMN2 depletion and neuroepithelium disintegration)****. We also show that these damages are* ***not rescued by ACV****, but that they are (almost completely)* ***prevented when*** *treating infected organoids* ***with ACV and anti-inflammation drugs****. This represents the first cellular and molecular analysis of HSV1 infection and ACV treatment in a human HSE model. Of note, Boivin et al 2013 reported a benefit of VCV and anti-TNF cotreatment in a mouse HSE model essentially only measuring mouse survival, while our study is a human model and presents a basis for understanding the molecular mechanisms involved.*
4) *Lastly, it seems that Ref 2 commented on the novelty of our paper based on comparison to our preprint Rybak-Wolf et al bioRxiv. This is unfortunate since the current manuscript* ***simply is the originally preprint, just (we think) vastly improved****.*

***We would like to highlight the new experimental data we generated and added to the manuscript to address the reviewers’ concerns:***

1) *We included all the missing controls suggested by reviewers, namely: a)* ***ACV-only-treated organoids*** *for the scRNA-seq analysis and for WB analysis. We observed no significant changes in cell type composition ACV-only-treated organoids (****new Fig S3B****, also reported in the point-by-point below) and no activation of TNF pathway introduced by ACV treatment alone, as expected (****new Fig S4B and S6A****, also reported here below and in the point-by-point); b)* ***mock medium treated controls*** *for immunostaining and WB analysis, to demonstrate no effect on the TNF pathway activation by mock medium alone (****new Fig S6A****, also reported here below).*
2) *We performed* ***experiments with a replication-deficient ICP27 knockout virus****. We could demonstrate that even the virus with impaired replication capacity is able to induce the inflammatory responses, as seen by activation of p65 phosphorylation, indicating that activation of NF-kB is independent of viral replication (****new Fig 6C, D****, also reported here below). Also damaged of neuronal processes (STMN2 expression) and changes in the neuroepithelial cells integrity were observed in organoids infected with ICP27 knockout virus, although to lower degree than in organoids infected with WT HSV-1 virus (****new Fig S6D-E****, also reported here below)****.***
3) *We* ***exposed naïve brain organoids to the supernatant collected from infected organoids*** *depleted of virus in order to assess whether p65 phosphorylation in infected organoids is mediated by signaling molecules (most likely cytokines) released during viral infection. The p65 phosphorylation in these organoids was significantly elevated compared to supernatant from mock-exposed organoids, suggesting that* ***infected organoids release signaling molecules capable of NF-kB pathway activation independently from viral infection****, providing a molecular mechanism for our observations (****new Fig S6F-I****, also reported here below).*
4) *We* ***added spatial transcriptomics data*** *(10x “Visium” technology) which show how the virus is spreading from the exterior to the interior of organoids (****new Fig S2A****), and how well these data correlate with GFP protein immunostaining (Fig 2B).*
5) *We clarified all remaining concerns, particularly regarding the GFP protein quantification as a proxy for viral load/replication and performed thorough quantifications of viral GFP at the DNA, RNA and protein level (****new Fig. S5B and H****, also reported in the point-by-point below), the comparison with existing organoids single-cell datasets (****new Fig. S1E****), and the STMN2 quantification (****new Fig. S2G****).*

**Fig.6**

**A.	B.**

***** p=0.01

***** p=0.03

+HSV-1

P-P65

GAPDH GFP (HSV-1)

**C.	D**

*p=0.025*

~~*~~

*p=0.013*

~~*~~

p-p65

ICP0

GAPDH

**Figure S6**

**A.	B.	C.**

p-P65

GAPDH GFP

p-P65

GAPDH GFP

p-P65 GAPDH

ICP0

**D.**	MOCK	HSV-1 (WT)	HSV-1 (ICP27 MUT)	**E.**

HSV-1-ICP0

STMN2

HSV-1-ICP0

ZO-1

MOCK	HSV-1 (WT)	HSV-1 (ICP27 MUT)

**F.	G.**

HSV-1-GFP

3days

medium 2x0.1µm filtered

3days

HSV-1 MEDIUM

HSV-1

MOCK

medium+ACV

MERGE

MERGE

**H.**

p-P65

GFP

BF

GAPDH GFP

**I.**

*p=0.039*

*~~*~~*

# Reviewer #1 (Remarks to the Author):

## In this manuscript, Wolf, Wyler, and Pentimali et al establish cerebral organoids as models for HSV-1 infection. The author apply single cell RNASeq of infected organoids to define the host pathways induces by HSV-1 infection. In addition, they show that while treatment with acyclovir inhibited viral replication, as expected, it was unable to control the induction of signaling pathways such as the TNF pathway, indicating that antiviral therapy alone may not be sufficient to prevent HSV-1-mediated neuronal damage. The manuscript is overall well-done and the most of the conclusions justified by the data provided. However, the lack of data to define the pathway(s) by which inflammatory pathways are activated in the absence of replication and the lack of depth in the studies regarding the combinatorial approaches suggested (e.g., in vivo) detract from the other aspects of the manuscript. My specific comments are below.

1. Cerebral organoids vary by size greatly and it is unclear how the authors calculate MOI based on "estimated number of cells at the surface of the organoid". More details are required regarding the calculation of MOI, which may be quite difficult given the organoid system.

We have calculated the surface cell number based on the total number of cells, assuming that the organoid is a sphere. For example, an organoid with 1 million cells has a radius of 62 cells (4/3*π*62^3^

= 10^6^). The surface consists therefore of about 4*π*62^2^=50,000 cells. This of course is only an estimation. We have rephrased the corresponding part in the methods section and now simply state that we used certain number of viral particles per organoid (p14, section: Viral infections).

## 1. An important control is missing as a ACV alone condition would be important to ensure that the pathways being induced are not associated with this treatment.

We agree with the referee that this is an important control, and we have now included single-cell sequencing and western blot data of ACV-only-treated organoids in the manuscript.

Based on these data, we conclude that there is no change in cell identity introduced by ACV treatment alone - as shown below (UMAP) (now in new **Fig S3B**) and no activation of TNF pathway observed (box plot) (now in new **Fig S4B**). Furthermore, we also confirmed this by western blot analysis (now in new **Figure S6A**).

CTRL CTRL+ACV

HSV-1 1dpi HSV-1 3dpi

HSV-1 3dpi +ACV

p-P65

GAPDH GFP

## 2. Figure 3D is not mentioned in the text.

We apologize for the oversight and have now corrected this mistake (p7, line 6).

## 3. Single cell reconstruction of cerebral organoids has been published by others (Kanton et al, Fiorenzano et al, Camp et al) and the authors should provide a more detailed discussion regarding these studies.

We in fact carefully compared our data to the mentioned papers when we defined the cell types and annotated the clusters obtained from our scRNA-seq data. We have now more clearly reported this in our manuscript and added comparisons in the supplement (**new Figure S1E)** (text p4, lines 14-15).

# Reviewer #2 (Remarks to the Author):

## The manuscript by Rybak-Wolf et al. describes human stem cell derived cerebral organoids demonstrating morphological as well as functional tissue integrity changes upon HSV-1 infection. HSV-1 is one of the most common pathogens leading to fatal human encephalitis. The pathogenesis is largely unclear. However, treatment with acyclovir improves outcomes of the disease, but patients still suffer from severe neurological sequelae. The authors of the present study particularly figured out that in contrast to antiviral therapy with acyclovir alone, tissue damage of organoids is minimal or even absent with combined anti-viral and anti-inflammatory therapy.

The results of this study are presented in a comprehensive and comprehensible manner, although the progress in knowledge regarding better therapy options is not entirely new (please see Boivin et al. 2013). Boivin and colleagues used a murine mouse model of herpes simplex virus encephalitis and made similar conclusions on combined therapy for HSE. Nevertheless, I recommend to accept the manuscript for publication with minor points which have to be modified as listed below.

We thank reviewer #2 for the positive evaluation of our work. We fully agree regarding Boivin et al. which we have, in our opinion appropriately, cited in the discussion (reference 54). However, we would like to stress, that we came to these conclusions using a human model and a completely unbiased approach, as our single-cell transcriptomic analysis indicates that the TNF/NFkB pathway is essentially the **only** one upregulated across all cell types analyzed in both infected and ACV-treated organoids. Furthermore, the molecular or cellular characterization of the infected and treated human model points towards specific changes related to neuronal damage (STMN2 depletion), neuroepithelium disintegration (ZO-1 stainings) and activation of NFkB pathways (p65 phosphorylation), which we think are important steps forward and building on what was published in 2013 by Boivin et al.

## Lines 59-61: It is well-known that animal models for HSE largely lack analogies to human disease. Please describe the most important weaknesses of animal models in more detail and emphasize the advantage of your organoid model compared to murine models for instance.

We have added some considerations on that matter in the discussion (p12, line 26-32, p13, line 1-5): “Several mouse models with different age, genetic background, inoculation routes, and virus strains of variable neurovirulence have been developed to investigate the pathogenesis of HSV1 infections in vivo (Mancini et al., 2018). So far, mostly intranasal or intracerebral infection models have been used to study HSE in mice and the infection pattern observed was more diffuse than that observed in HSE patients, indicating that mechanism of virus spreading can be different in human. For example, the virus did not spread to the temporal and frontal lobes, which are the typical locations in HSE in humans. HSV-1 is a human specific virus, therefore in order to precisely model its pathogenesis, host response and to develop preventive and therapeutic treatments we need to use human models. iPSC derived organoids partially recapitulate the complexity and many characteristics of in vivo disease. For example, some aspects such as pre-long infections or latency are difficult to model in 2D culture, but were possible in organoids. (D’Aiuto et al., 2019)”

## Line 97: The organoids revealed dorsal forebrain region. Is it morphologically comparable to the temporal/frontal lobe which is commonly affected in HSE?

With the unguided cerebral organoid protocol that we implemented, we mainly model the dorsal forebrain region, but also subsets of subcortical neurons as shown in Figure 1. Since organoid models only reflect early brain development, it is difficult to compare them to the adult temporal/frontal lobes. They are rather similar to the earlier diencephalic and telencephalic stages, from which these regions originate. Of note, brain organoids are still the most complex human brain tissue models currently available for in vitro studies. We have added a clarifying sentence to the results section (p4, line 5-8) and in the discussion (p12, line 13-16).

## Line 118: I would prefer to designate the full name of the virus strain used.

We now added the full strain name (17 syn+) to the text (page 4, line 21).

## >>> Lines 152-154: Figure 2E indicates immunohistochemistry/immunofluorescence. How did the authors quantify the signals? Please clarify/modify the corresponding figure (as indicated in the material and methods section).

We modified the text and added quantification of STMN2 mRNA expression to **new Fig S2G**. The protein quantification is done later on in **Fig S5H**.

## >>> Lines 155-161: Neuroepithelial identity changes were analyzed by ZO-1 immunostaining. To underline this finding, is there another useful cell polarity marker?

In Krenn et al, 2021, N-cadherin was used to determine neuroepithelial integrity in the very early stage of organoid development. N-cadherin, similarly to ZO-1 is enriched at the apical side in cells surrounding the lumen. Both markers are valid markers to determine neuroepithelial polarity or integrity.

Due to the limited time frame for this revision, we could only test one N-cad antibody which unfortunately did not work (no signal detected). However, we would like to emphasize that not only ZO- 1 staining itself can be used for the identity changes in the neuroepithelial identity, but the overall structural changes in the neuroepithelial rosettes can also be determined by SOX-2 staining or by DAPI nuclear staining, as shown in **Fig 4,** in addition to ZO-1 staining.

## >>> Line 169: The term “affects cellular identities” is confusing. Would it be better to use “cellular composition” instead of “cellular identities”?

and

## >>> Lines 199: Please specify what “highly infected” means.

We have changed the title of this section to “HSV-1 infection affects cellular compositions in cerebral organoids”. Furthermore, we have added some clarifications on the observations regarding the changes in cell characteristics and “highly infected” cells (page 7, line3-6).

## >>> Lines 213-215: How long are cells “highly infected”? Did the authors look beyond 3 dpi?

We have not looked beyond 3 dpi with a wild type virus, as organoids start to decompose. The “highly infected” state likely ends up in apoptosis/necrosis at later time points. We added a sentence to clarify this (page 7, line 22-23).

## >>> Lines 264-265: Please specify “tissue and cellular damage”. For the reader it is not clear which morphological changes or lesions are meant: necrosis, apoptosis, etc.

We have assessed the apoptosis by analyzing the cleaved Caspase-3 expression level in infected organoids (3dpi). We have not observed an increase in the level of cleaved Caspase-3 expression (see **new Fig. S4D**) (which is in line with previous publications showing that HSV-1 can effectively inhibit apoptosis- for example You et al., 2017), therefore we assume that apoptosis is not the main cause/driver of tissue damage in infected organoids. We can speculate that there are several changes which can contribute to it, namely damaged neuronal processes (STMN2 staining **Fig2E**) and changes in the neuroepithelial cells integrity (as shown by ZO-1 staining **Fig. 2F**) and we now defined this better in the text (p8, line 26-28).

>>> Line 425: Please modify: “For immunostaining, … and fixed in 4%...”

>>> Line 435: Please modify: “…overnight, and washed three times…”

>>> Line 436: Please modify: “…at RT for 2h, stained…”

>>> Lines 432-437: Please shorten this long sentence.

>>> Figure 6: Please change figure caption: “B” is designated as “G”.

We thank the referee for the careful reading, we have now corrected these sentences.

# Reviewer #3 (Remarks to the Author):

## Mechanisms driving HSV-1 infection of the human brain and associated pathologies are poorly understood. Given the fact that HSV-1 causes encephalitis and the possibility that it may affect neurodegeneration in the CNS, multiple attempts have been made to model the consequences of HSV- 1 infection via human iPSC or ESC 3D cultures often termed as brain organoids or in this case, cerebral organoids. Past studies (summarized in Depla et al., Viruses, 2022; Cairns et al., 2020, Sci Adv) and a 2021 bioRxiv paper by the authors (Rybak-Wolf et al.) elegantly demonstrate the use of such 3D brain organoid in HSV-1 infection and infection-associated neuronal loss. A major strength of the manuscript is that it is well presented and well written. A few major weaknesses however significantly reduce enthusiasm. A major problem is that the findings are mostly descriptive, lack proper controls and often fail to go too much beyond what is already known in the field. In other words, it lacks depth and the novelty is incremental. Some other issues are listed below.

1. Fig. 1, very little new information is gained from this figure. Similar information can also be found in their 2021 bioRxiv paper.

Our current manuscript originates from that preprint, which was presenting a rather general description of multiple modalities of molecular characterization of HSV1 infection of brain organoids. In a subsequent effort, we focused on the TNF pathway which was the strongest signature observed

from these data, and collected our additional results in the manuscript submitted here. For this reason, Figure 1 of this manuscript presents similar (but more thorough and clearer) results than that in our preprint, as this manuscript is a “version 2” of that preprint. We will replace the old version with the current manuscript version on bioRxiv after re-submission of our manuscript.

## 1. Fig. 2, a large portion of the results shown here have already been described elsewhere using 3D brain organoids (please see the references above). Calcium imaging data lack evidence whether active viral replication is required or not. Long time ago Cheshenko et al. (JCB, 2003) showed that Ca signaling is needed during viral entry. Use of a replication deficient virus can provide more knowledge on this phenomenon and better explain the observations made in this figure.

The purpose of calcium imaging experiment was only to demonstrate the loss of coordinated neuronal activity upon HSV-1 infection. However, we thank the referee regarding the replication deficient virus comment and we now used such a virus (ICP27 knockout) in experiments introduced below.

## 2. Fig. 3, did the authors use sucrose density gradient purified virus here and in the previous figure? If infected cell lysates were used (most common), then many inflammatory/toxic substances present in the lysates can also cause the observations made here. Their control is no viral infection; a true scientific control would be lysates from noninfected cells if the infected cell lysates were used for the infection. Fig 3D, ACV alone control is missing.

We performed the experiments with cell lysates as well as with concentrated purified virus (all experiments with WT and ICP27 mutant in Fig 6 and S6), observing similar effects. Also, the volume of added lysate was never exceeding 1-5% of the total culture medium, and was less than 0.03% for concentrated virus. Therefore, the concentration of

inflammatory/toxic substances added together with virus was too low to induce inflammatory response. However, to better address this concern, we included an additional non-infected cells lysate as a control for immunostainings/Western analyses (**new Fig 6C-D and Fig S6**). Furthermore, we performed an experiment where we exposed organoids to irradiated (inactive) HSV-1-GFP virus and non-irradiated HSV- 1-GFP(active) virus with the same volume of lysate (5%) added and observed an activation of inflammatory responses (p-P65) only with active virus. This indicates that small volume of cell lysate added to organoids cannot drive observed inflammatory responses (**new Fig S6B**).

## 3. Figs. 4/5, It is gratifying that TNF signaling and the inability of ACV to reduce its activity are also recapitulated well in this model. However, this knowledge already exists from previous animal studies and human clinical observations. The data shown here are descriptive and provide very little new knowledge. Again, ACV alone control is missing, which makes this study less rigorous.

We now added the ACV alone control (**new Fig S6A**) and we thank the referee for pointing out this important control.

However, regarding the other points, to our knowledge, there are no clinical data pointing towards TNF activation after ACV treatment, only clinical observations related to neurological sequelae. Also, in Boivin et al., 2013, only a survival analysis was performed in mice, without any molecular characterization. It is true that it was shown previously that TNF is activated after HSV-1 infection, but there is no study showing neither (a) that this activation persists after ACV treatment nor (b) that

targeting this pathway **prevents** molecular, cellular or tissue-level effects of the infection in a neural tissue. In our paper, we could clearly demonstrate using scRNAseq analysis, immunostaining and western blotting that not only TNF pathway activation remains after ACV treatment, but also that cellular and tissue changes (neuroepithelial integrity, STMN2 expression) associated with viral infection are only partially prevented by current treatments, while complementary treatments that target the inflammatory response better prevent such changes.

## 4. Fig. 5C/D GFP intensity for the treated organoids never reaches the control levels. Still the data in Fig.6 claims near complete inhibition of the infection. How to truly reconcile the two apparently contrasting results? If GFP intensity has no real meaning, then why to show the data?

In all the data presented in our study, ACV treatment causes a significant downregulation of GFP expression, although not a complete depletion (not even in the Western in Fig. 6A), since ACVonly inhibits viral replication but not infection. IF images are usually centered on the external part of the organoids, which is more accessible and more infected (more GFP detected), while Westerns are performed on lysates from whole organoids, where GFP expression is averaged over the entire organoids tissue. To further address this point:

- *We now provide immunofluorescence quantifications in* ***new Fig. S5H*** *for two MOIs; in these two cases, as we would expect, the viral GFP signal is differently impacted by the treatment: with viral replication being controlled much better at lower MOI, compared to higher MOI;*
- *In Fig. 6A, we show Western blots from experiments performed with a similar MOI than that of the “low MOI” treatment in Fig. S5G;*
- *To quantify whether the Western and Immunostainings are in good agreement (and therefore that the GFP signal is indeed meaningful), we show below a scatterplot of the average GFP intensity from the two readouts (Western blots with low MOI and immunofluorescence with low and high MOI) in each condition: the low MOI IF GFP signal is indeed very well correlated to the Western GFP signal (Pearson = 0.979, green dots).*

Furthermore, we have now measured viral GFP expression on DNA, RNA and protein level in organoids from the same batch and could show that there is a good correlation between GFP DNA, mRNA and protein, therefore we can assume that GFP protein levels as assessed by Western or IF is a good proxy for viral load. We added these data to a supplementary figure in our manuscript (**new Fig S5B and S5H**). Furthermore, we included also the visium spatial transcriptomic data showing the GFP RNA expression thought the organoids ( **new Fig S2A**).

## How deep into the tissue the drugs can penetrate? What would be the mechanism?

We have added sections of organoids infected with the GFP virus that show clearly that upon ACV treatment, no GFP signal is seen in the interior of the organoid, which suggests that we have complete drug penetration, and therefore also repressed viral replication in the interior of the organoid (**new Fig. S3A and S5A**)**.**

HSV-1 GFP	MERGE

DAPI HSV-1

DAPI HSV-1

HSV-1 3dpi

## Since they are basically reproducing the prior knowledge from other model systems, the authors should deliver the neurovirulent Y34.5 protein of HSV-1 to demonstrate a viral determinant of the cytopathic/inflammatory effects observed in their cerebral organoids. This way the study will demonstrate a molecular mechanism by which HSV-1 causes damage in the organoids and be less descriptive. Alternatively, the non-neurovirulent HSV-1 Y34.5 deletion virus should be compared with the wildtype virus.

HSV-1 3dpi+ACV

The main claim of our paper is that the damage to the organoids is to a substantial degree mediated by the inflammatory response caused by the virus. In order to further substantiate this claim, we have now added two new experiments. First, we tested the reaction of organoids to filtered supernatant from infected organoids, i.e. we transfer inflammatory mediators such as cytokines, but not the virus itself. We have indeed seen that this induces substantial significant effects on p-65 phosphorylation (**new Fig**. **6E-H**). Second, we use a replication-deficient ICP27 knockout virus (along with the KOS strain wt virus, since the ICP27 knockout is done in the KOS background). As expected, the ICP27 knockout virus can only be seen in the outer cell layer of the organoids, since it cannot replicate and invade the inner parts of the organoids. Still, we observed strong NF-kB activation in the ∆ICP27 HSV-1 infected organoids, as well as substantial structural damage in the vicinity of the infected cell layer (**new Fig. 6 C, D** and **S6C-E**). This again argues that non-cell autonomous effects cause organoid tissue damage.

**Decision Letter, second revision:**

| **Message:** | 17th January 2023  *Please ensure you delete the link to your author homepage in this e-mail if you wish to forward it to your co-authors.  Dear Nikolaus and Agnieszka,  Thank you for providing such detailed answers to my question and for providing the additional analysis. We are very interested in the possibility of publishing your study in Nature Microbiology, and think it's best to revise your manuscript and the point-by-point response according to your suggestions to address the points by reviewer #1, before we send it for another round of peer-review.  In particular, please add your:  - bulk RNA-seq data analysis from HSV-1 infected organoids showing increased expression of TNF-alpha ligands upon viral infection.  - sc-RNA-seq data showing that expression of TNF-alpha ligands remain high even with ACV treatment.  - WB analysis showing phosphorylation of ERK in organoids infected with WT and ICP27 mutant HSV-1.  We are committed to providing a fair and constructive peer-review process. Do not hesitate to contact us if there are specific requests from the reviewers that you believe are technically impossible or unlikely to yield a meaningful outcome.  If you have not done so already please begin to revise your manuscript so that it conforms to our Article format instructions at http://www.nature.com/nmicrobiol/info/final-submission/  The usual length limit for a Nature Microbiology Article is six display items (figures or tables) and 3,000 words. We have some flexibility, and can allow a revised manuscript at 3,500 words, but please consider this a firm upper limit. There is a trade-off of ~250 words per display item, so if you need more space, you could move a Figure or Table to Supplementary Information.  Some reduction could be achieved by focusing any introductory material and moving it to the start of your opening ‘bold’ paragraph, whose function is to outline the background to your work, describe in a sentence your new observations, and explain your main conclusions. The discussion should also be limited. Methods should be described in a separate section following the discussion, we do not place a word limit on Methods.  Nature Microbiology titles should give a sense of the main new findings of a manuscript, and should not contain punctuation. Please keep in mind that we strongly discourage active verbs in titles, and that they should ideally fit within 90 characters each (including spaces).  We strongly support public availability of data. Please place the data used in your paper into a public data repository, if one exists, or alternatively, present the data as Source Data or Supplementary Information. If data can only be shared on request, please explain why in your Data Availability Statement, and also in the correspondence with your editor. For some data types, deposition in a public repository is mandatory - more information on our data deposition policies and available repositories can be found at https://www.nature.com/nature-research/editorial-policies/reporting-standards#availability-of-data.  Please include a data availability statement as a separate section after Methods but before references, under the heading "Data Availability”. This section should inform readers about the availability of the data used to support the conclusions of your study. This information includes accession codes to public repositories (data banks for protein, DNA or RNA sequences, microarray, proteomics data etc…), references to source data published alongside the paper, unique identifiers such as URLs to data repository entries, or data set DOIs, and any other statement about data availability. At a minimum, you should include the following statement: “The data that support the findings of this study are available from the corresponding author upon request”, mentioning any restrictions on availability. If DOIs are provided, we also strongly encourage including these in the Reference list (authors, title, publisher (repository name), identifier, year). For more guidance on how to write this section please see: http://www.nature.com/authors/policies/data/data-availability-statements-data-citations.pdf  To improve the accessibility of your paper to readers from other research areas, please pay particular attention to the wording of the paper’s opening bold paragraph, which serves both as an introduction and as a brief, non-technical summary in about 150 words. If, however, you require one or two extra sentences to explain your work clearly, please include them even if the paragraph is over-length as a result. The opening paragraph should not contain references. Because scientists from other sub-disciplines will be interested in your results and their implications, it is important to explain essential but specialised terms concisely. We suggest you show your summary paragraph to colleagues in other fields to uncover any problematic concepts.  If your paper is accepted for publication, we will edit your display items electronically so they conform to our house style and will reproduce clearly in print. If necessary, we will re-size figures to fit single or double column width. If your figures contain several parts, the parts should form a neat rectangle when assembled. Choosing the right electronic format at this stage will speed up the processing of your paper and give the best possible results in print. We would like the figures to be supplied as vector files - EPS, PDF, AI or postscript (PS) file formats (not raster or bitmap files), preferably generated with vector-graphics software (Adobe Illustrator for example). Please try to ensure that all figures are non-flattened and fully editable. All images should be at least 300 dpi resolution (when figures are scaled to approximately the size that they are to be printed at) and in RGB colour format. Please do not submit Jpeg or flattened TIFF files. Please see also 'Guidelines for Electronic Submission of Figures' at the end of this letter for further detail.  Figure legends must provide a brief description of the figure and the symbols used, within 350 words, including definitions of any error bars employed in the figures.  When submitting the revised version of your manuscript, please pay close attention to our href="https://www.nature.com/nature-research/editorial-policies/image-integrity">Digital Image Integrity Guidelines.</a> and to the following points below:  -- that unprocessed scans are clearly labelled and match the gels and western blots presented in figures. -- that control panels for gels and western blots are appropriately described as loading on sample processing controls -- all images in the paper are checked for duplication of panels and for splicing of gel lanes.   Finally, please ensure that you retain unprocessed data and metadata files after publication, ideally archiving data in perpetuity, as these may be requested during the peer review and production process or after publication if any issues arise.   Please include a statement before the acknowledgements naming the author to whom correspondence and requests for materials should be addressed.  Finally, we require authors to include a statement of their individual contributions to the paper -- such as experimental work, project planning, data analysis, etc. -- immediately after the acknowledgements. The statement should be short, and refer to authors by their initials. For details please see the Authorship section of our joint Editorial policies at http://www.nature.com/authors/editorial_policies/authorship.html  When revising your paper:  * include a point-by-point response to any editorial suggestions and to our referees. Please include your response to the editorial suggestions in your cover letter, and please upload your response to the referees as a separate document.  * ensure it complies with our format requirements for Letters as set out in our guide to authors at www.nature.com/nmicrobiol/info/gta/  * state in a cover note the length of the text, methods and legends; the number of references; number and estimated final size of figures and tables  * resubmit electronically if possible using the link below to access your home page:  [redcated]  *This url links to your confidential homepage and associated information about manuscripts you may have submitted or be reviewing for us. If you wish to forward this e-mail to co-authors, please delete this link to your homepage first.  Please ensure that all correspondence is marked with your Nature Microbiology reference number in the subject line.  Nature Microbiology is committed to improving transparency in authorship. As part of our efforts in this direction, we are now requesting that all authors identified as ‘corresponding author’ on published papers create and link their Open Researcher and Contributor Identifier (ORCID) with their account on the Manuscript Tracking System (MTS), prior to acceptance. This applies to primary research papers only. ORCID helps the scientific community achieve unambiguous attribution of all scholarly contributions. You can create and link your ORCID from the home page of the MTS by clicking on ‘Modify my Springer Nature account’. For more information please visit please visit <a href="http://www.springernature.com/orcid">www.springernature.com/orcid</a>.  We hope to receive your revised paper within three weeks. If you cannot send it within this time, please let us know.  We look forward to hearing from you soon.  Yours sincerely, |
| --- | --- |

**Author Rebuttal, second revision:**

Dear Reviewers,

We thank you very much for your thoughtful evaluation of our work. We went carefully through all the point raised and we are happy to read that:

- “The manuscript is overall well-done and the most of the conclusions justified by the data provided.”
- “The results of this study are presented in a comprehensive and comprehensible manner.”
- “I recommend to accept the manuscript for publication with minor points.”

It is our understanding the main concerns regarding the suitability of our paper for publication in Nature Microbiology lie in the lack of novelty of our findings, specifically:

- “[…] the progress in knowledge regarding better therapy options is not entirely new (please see Boivin et al. 2013).”
- “Boivin and colleagues used a murine mouse model of herpes simplex virus encephalitis and made similar conclusions on combined therapy for HSE.”
- “A major problem is that the findings are mostly descriptive, lack proper controls and often fail to go too much beyond what is already known in the field.”

*We provide below* ***a summary of the major experimental data added*** *to the paper in response to the referees’ comments. We then provide a* ***detailed and exhaustive point-by-point response****. However, we would first like to comment on* ***the novelty*** *and medical significance of our work:*

1) ***Generation of the first human model of ACV-treated HSE****. Acyclovir (ACV) revolutionized the treatment of patients with Herpes induces Encephalitis (HSE), increasing survival from ~30% to*

~90% (Riera-Mestre *et al*. 2009). Nevertheless, 70% of the surviving patients retain neurological damage. This shows a great clinical need, namely not only to ensure survival but also to reduce long-term consequences of the infection. Unfortunately, this need is very difficult to address since live brain tissues from these patients obviously cannot be procured. For these reasons, models are needed to study the molecular mechanisms involved. Mouse models are problematic because, as referee 2 rightly points out, as “*It is well-known that animal models for HSE largely lack analogies to human disease*”. Here, we present the **first human model for ACV treatment** in a neural tissue, and demonstrate that our infected organoids show molecular and cellular phenotypes which are **consistent with HSE pathology in patients**. We also show that co-treatment with anti-inflammatory drugs prevents these phenotypes, providing evidence that co-treatment is relevant for HSE patients.

2) *In our system, comprehensive single-cell analyses show that* ***TNFα/NFKB is likely the only pathway by which the clinical outcome can be improved****. This is, we think, a very important result as it suggests that co-treatment with anti-inflammatory drugs that act on that pathway can potentially prevent neuronal damage caused by HSV despite treatment with ACV.*
3) ***On the molecular and tissue level, we show that HSV-1 causes phosphorylation of NFKB subunit p65 and tissue damage (STMN2 depletion and neuroepithelium disintegration)****. We also show that these damages are* ***not rescued by ACV****, but that they are (almost completely)* ***prevented when*** *treating infected organoids* ***with ACV and anti-inflammatory drugs****. This represents the first cellular and molecular analysis of HSV1 infection and ACV treatment in a human HSE model. Of note, Boivin et al. (2013) reported a benefit of VCV and anti-TNFα* *co-treatment in a mouse HSE model essentially only measuring mouse survival, while our study presents a human model and an unbiased molecular characterization of infection and treatment. Furthermore, with the new data included in this revision, we provide the basis for understanding the molecular mechanisms involved.*
4) *Lastly, it seems that Ref 2 commented on the novelty of our paper based on comparison to our preprint Rybak-Wolf et al. (bioRxiv, 2021). Our current manuscript originates from that preprint,*

which was presenting a rather general description of multiple modalities of molecular characterization of HSV-1 infection of brain organoids. We have no intention to submit this bioRxiv preprint elsewhere, but replace it with the current manuscript, which contains vastly more data and conceptual insights.

***We would like to highlight the new experimental data we generated and added to the manuscript to address the reviewers’ concerns:***

1) *We included all the missing controls suggested by reviewers, namely: a)* ***ACV-only-treated organoids*** *for the scRNA-seq analysis and for WB analysis. We observed no significant changes in cell type composition ACV-only-treated organoids (****new Fig S3b****, also reported in the point-by-point below) and no activation of TNF pathway introduced by ACV treatment alone, as expected (****new Fig S4b and S6a****, also reported here below and in the point-by-point); b)* ***mock medium treated controls*** *for immunostaining and WB analysis, to demonstrate no effect on the TNF pathway activation by mock medium alone (****new Fig S6a****, also reported here below).*
2) *We generated and analyzed new bulk RNA-seq data, as well as reanalyzed our single-cell RNAseq data, to quantify the expression of ligands/cytokines activating the TNFα/NFKB pathway. We found* ***robust up-regulation of pretty much all TNF receptor ligands upon HSV-1 infection, with few such as TNFSF9 and TNF*** *itself standing out as extremely highly induced (****new Fig S4d,e****). This upregulation of TNF ligands was confirmed in scRNA-seq data also for ACV-treated organoids.*
3) *We performed* ***experiments with a replication-deficient ICP27 knockout virus****. We could demonstrate that even the virus with impaired replication capacity is able to induce the inflammatory response, as seen by activation of p65 phosphorylation, indicating that activation of NFKB is independent of viral replication (****new Fig 6c,d****, also reported here below). Also, damage of neuronal processes (STMN2 expression) and changes in the neuroepithelial cells integrity were observed in organoids infected with the ICP27 knockout virus, although to a lower degree with respect to organoids infected with the WT HSV-1 virus (****new Fig S6d,e****, also reported here below)****.***
4) *We further investigated the activation of several upstream factors leading to NFKB p65 phosphorylation. We could show that both wild-type and ICP27 deletion mutant lead to activation of pAKT and ERK1/2 (new* ***Fig S6f****).*
5) *We* ***exposed naïve brain organoids to the supernatant collected from infected organoids*** *depleted of virus in order to assess whether p65 phosphorylation in infected organoids is mediated by signaling molecules (most likely cytokines) released during viral infection. The p65 phosphorylation in these organoids was significantly elevated compared to supernatant from mock-exposed organoids, suggesting that* ***infected organoids release signaling molecules capable of NFKB pathway activation independently from viral infection****, providing a molecular mechanism for our observations (****new Fig S6g-j****, also reported here below).*
6) *We* ***added spatial transcriptomics data*** *(10x “Visium” technology) which show how the virus is spreading from the exterior to the interior of organoids (****new Fig S2a****), and how well these data correlate with GFP protein immunostaining (Fig 2b).*
7) *We clarified all remaining concerns, particularly regarding the GFP protein quantification as a proxy for viral load/replication and performed thorough quantifications of viral GFP at the DNA, RNA and protein level (****new Fig. S5b and h****, also reported in the point-by-point below), the comparison with existing organoids single-cell datasets (****new Fig. S1e****), and the STMN2 quantification (****new Fig. S2g****).*

**Fig.6**

**A.	B.**

***** p=0.01

***** p=0.03

+HSV-1

P-P65

GAPDH GFP (HSV-1)

**C.	D**

*p=0.025*

~~*~~

*p=0.013*

~~*~~

p-p65

ICP0

GAPDH

**Fig. S6**

**a	b	c**

p-P65

GAPDH GFP

p-P65

GAPDH GFP

p-P65 GAPDH

ICP0

**d**	MOCK	HSV-1 (WT)	HSV-1 (ICP27 MUT)	**e**

HSV-1-ICP0

ZO-1

MOCK	HSV-1 (WT)	HSV-1 (ICP27 MUT)

HSV-1-ICP0

STMN2

**f	g**

MERGE

**h**

HSV-1-GFP

3days

medium 2x0.1µm filtered

3days

HSV-1 MEDIUM

HSV-1

MOCK

MERGE

ICP0

BF

p-P65

GFP

AKT

pAKT

pERK1/2

*p=0.039*

*~~*~~*

**i	j**

TRAF6

medium+ACV

GAPDH

p-P65

GAPDH GFP

# Reviewer #1 (Remarks to the Author):

In this manuscript, Wolf, Wyler, and Pentimali et al establish cerebral organoids as models for HSV-1 infection. The author apply single cell RNASeq of infected organoids to define the host pathways induces by HSV-1 infection. In addition, they show that while treatment with acyclovir inhibited viral replication, as expected, it was unable to control the induction of signaling pathways such as the TNF pathway, indicating that antiviral therapy alone may not be sufficient to prevent HSV-1-mediated neuronal damage. The manuscript is overall well-done and the most of the conclusions justified by the data provided. However, the lack of data to define the pathway(s) by which inflammatory pathways are activated in the absence of replication and the lack of depth in the studies regarding the combinatorial approaches suggested (e.g., in vivo) detract from the other aspects of the manuscript. My specific comments are below.

To address referee’s question regarding inflammatory pathways activated by HSV-1*,* we looked into bulk RNA-seq data to check for the expression of ligands/cytokines activating the TNFα/NFKB pathway. We found robust up-regulation of pretty much all TNF receptor ligands upon HSV1 infection, with few such as TNFSF9 and TNF itself standing out as extremely highly induced (**new Fig S4d**). We also checked our single-cell RNAseq data, which are more sparse (so we cannot detect as many ligands as in the bulk data) but for which we also have ACV-treated organoids, and we confirmed that not only most of the detected ligands are up-regulated upon infection, but also upon ACV treatment (**new Fig S4e**). We further investigated the activation of several upstream factors leading to NFKB p65 phosphorylation. We could show that both wild-type and ICP27 deletion mutant lead to activation of pAKT and ERK1/2 (new Fig S6f).These data are also reported here below.

Regarding the in vivo data, we cited in the discussion a study (Boivin et al., 2013) where a mouse model of herpes simplex virus encephalitis was used for modeling a combined therapy and which demonstrated better mouse survival after combining VCV and anti-TNFα. We think that our unbiased molecular analysis, taken together with prior in vivo data, makes our conclusions even more relevant with respect to the potential therapeutic outcome of combining antiviral and anti-inflammatory treatments.

**Fig. S4 d**

**e	f**

1. Cerebral organoids vary by size greatly and it is unclear how the authors calculate MOI based on "estimated number of cells at the surface of the organoid". More details are required regarding the calculation of MOI, which may be quite difficult given the organoid system.

We have calculated the surface cell number based on the total number of cells, assuming that the organoid is a sphere. For example, an organoid with 1 million cells has a radius of 62 cells (4/3*π*62^3^

= 10^6^). The surface consists therefore of about 4*π*62^2^=50,000 cells. This of course is only an estimation. We have rephrased the corresponding part in the methods section and now simply state that we used certain number of viral particles per organoid (p14, section: Viral infections).

1. An important control is missing as a ACV alone condition would be important to ensure that the pathways being induced are not associated with this treatment.

We agree with the referee that this is an important control, and we have now included single-cell sequencing and western blot data of ACV-only-treated organoids in the manuscript.

Based on these data, we conclude that there is no change in cell identity introduced by ACV treatment alone - as shown below (UMAP) (now in new **Fig S3b**) and no activation of TNF pathway observed (box plot) (now in new **Fig S4b**). Furthermore, we also confirmed this by western blot analysis (now in new **Figure S6b**).

CTRL CTRL+ACV

HSV-1 1dpi HSV-1 3dpi

HSV-1 3dpi +ACV

p-P65

GAPDH GFP

2. Figure 3D is not mentioned in the text.

We apologize for the oversight and have now corrected this mistake (p7, line 11).

3. Single cell reconstruction of cerebral organoids has been published by others (Kanton et al, Fiorenzano et al, Camp et al) and the authors should provide a more detailed discussion regarding these studies.

We in fact carefully compared our data to the mentioned papers when we defined the cell types and annotated the clusters obtained from our scRNA-seq data. We have now more clearly reported this in our manuscript and added comparisons in the supplement (**new Figure S1e)** (text p4, lines 14-16).

# Reviewer #2 (Remarks to the Author):

The manuscript by Rybak-Wolf et al. describes human stem cell derived cerebral organoids demonstrating morphological as well as functional tissue integrity changes upon HSV-1 infection. HSV-1 is one of the most common pathogens leading to fatal human encephalitis. The pathogenesis is largely unclear. However, treatment with acyclovir improves outcomes of the disease, but patients still suffer from severe neurological sequelae. The authors of the present study particularly figured out that in contrast to antiviral therapy with acyclovir alone, tissue damage of organoids is minimal or even absent with combined anti-viral and anti-inflammatory therapy.

The results of this study are presented in a comprehensive and comprehensible manner, although the progress in knowledge regarding better therapy options is not entirely new (please see Boivin et al. 2013). Boivin and colleagues used a murine mouse model of herpes simplex virus encephalitis and

made similar conclusions on combined therapy for HSE. Nevertheless, I recommend to accept the manuscript for publication with minor points which have to be modified as listed below.

We thank reviewer #2 for the positive evaluation of our work. We fully agree regarding Boivin et al. (2023) which we have, in our opinion appropriately, cited in the discussion (reference 54). However, we would like to stress that we came to these conclusions using a human model and a completely unbiased approach, as our single-cell transcriptomic analysis indicates that the TNFα/NFKB pathway is essentially the **only** one upregulated across all cell types analyzed in both infected and ACV-treated organoids. Furthermore, the molecular or cellular characterization of the infected and treated human model points towards specific changes related to neuronal damage (STMN2 depletion), neuroepithelium disintegration (ZO-1 stainings) and activation of NFKB (p65 phosphorylation), which we think are important steps forward with respect to, and building on what was published in 2013 by Boivin et al.

Lines 59-61: It is well-known that animal models for HSE largely lack analogies to human disease. Please describe the most important weaknesses of animal models in more detail and emphasize the advantage of your organoid model compared to murine models for instance.

We have added some considerations on this matter in the discussion (p12, line 28-32, p13, line 11-22): “Several mouse models with different age, genetic background, inoculation routes, and virus strains of variable neurovirulence have been developed to investigate the pathogenesis of HSV1 infections in vivo (Mancini et al., 2018). So far, mostly intranasal or intracerebral infection models have been used to study HSE in mice and the infection pattern observed was more diffuse than that observed in HSE patients, indicating that mechanism of virus spreading can be different in human. For example, the virus did not spread to the temporal and frontal lobes, which are the typical locations in HSE in humans. HSV-1 is a human specific virus, therefore in order to precisely model its pathogenesis, host response and to develop preventive and therapeutic treatments we need to use human models. iPSC derived organoids partially recapitulate the complexity and many characteristics of in vivo disease. For example, some aspects such as pre-long infections or latency are difficult to model in 2D culture, but were possible in organoids. (D’Aiuto et al., 2019)”

Line 97: The organoids revealed dorsal forebrain region. Is it morphologically comparable to the temporal/frontal lobe which is commonly affected in HSE?

With the unguided cerebral organoid protocol that we implemented, we mainly model the dorsal forebrain region, but also subsets of subcortical neurons as shown in Figure 1. Since organoid models only reflect early brain development, it is difficult to compare them to the adult temporal/frontal lobes. They are rather similar to the earlier diencephalic and telencephalic stages, from which these regions originate. Of note, brain organoids are still the most complex human brain tissue models currently available for in vitro studies. We have added a clarifying sentence to the results section (p4, line 5-8) and in the discussion (p12, line 28-30).

Line 118: I would prefer to designate the full name of the virus strain used.

We now added the full strain name (17 syn+) to the text (page 4, line 22).

>>> Lines 152-154: Figure 2E indicates immunohistochemistry/immunofluorescence. How did the authors quantify the signals? Please clarify/modify the corresponding figure (as indicated in the material and methods section).

We modified the text and added quantification of STMN2 mRNA expression to **new Fig S2g**. The protein quantification is done later on in **Fig S5h**.

>>> Lines 155-161: Neuroepithelial identity changes were analyzed by ZO-1 immunostaining. To underline this finding, is there another useful cell polarity marker?

In Krenn et al., (2021), N-cadherin was used to determine neuroepithelial integrity in the very early stage of organoid development. N-cadherin, similarly to ZO-1, is enriched at the apical side in cells surrounding the lumen. Both markers are valid markers to determine neuroepithelial polarity or integrity. Due to the limited time frame for this revision, we could only test one N-cad antibody which unfortunately did not work (no signal detected). However, we would like to emphasize that not only ZO- 1 staining itself can be used for the identity changes in the neuroepithelial identity, but the overall structural changes in the neuroepithelial rosettes can also be determined by SOX-2 staining or by DAPI nuclear staining, as shown in **Fig 4,** in addition to ZO-1 staining.

>>> Line 169: The term “affects cellular identities” is confusing. Would it be better to use “cellular composition” instead of “cellular identities”?

and

>>> Lines 199: Please specify what “highly infected” means.

We have changed the title of this section to “HSV-1 infection affects cellular compositions in cerebral organoids”. Furthermore, we have added some clarifications on the observations regarding the changes in cell characteristics and “highly infected” cells (page 7, line 8-11).

>>> Lines 213-215: How long are cells “highly infected”? Did the authors look beyond 3 dpi?

We have not looked beyond 3 dpi with a wild type virus, as organoids start to decompose. The “highly infected” state likely ends up in apoptosis/necrosis at later time points. We added a sentence to clarify this (page 7, line 27-29).

>>> Lines 264-265: Please specify “tissue and cellular damage”. For the reader it is not clear which morphological changes or lesions are meant: necrosis, apoptosis, etc.

We have assessed the apoptosis by analyzing the cleaved Caspase-3 expression level in infected organoids (3dpi). We have not observed an increase in the level of cleaved Caspase-3 expression (see **new Fig. f**) (which is in line with previous publications showing that HSV-1 can effectively inhibit apoptosis- for example You et al., 2017), therefore we assume that apoptosis is not the main cause/driver of tissue damage in 1-3 dpi organoids. We can speculate that there are several changes which can contribute to it, namely damaged neuronal processes (STMN2 staining **Fig2e**) and changes in the neuroepithelial cells integrity (as shown by ZO-1 staining **Fig. 2f**) and we now defined this better in the text (p9, line 5-7).

>>> Line 425: Please modify: “For immunostaining, … and fixed in 4%...”

>>> Line 435: Please modify: “…overnight, and washed three times…”

>>> Line 436: Please modify: “…at RT for 2h, stained…”

>>> Lines 432-437: Please shorten this long sentence.

>>> Figure 6: Please change figure caption: “B” is designated as “G”.

We thank the referee for the careful reading, we have now corrected these sentences.

# Reviewer #3 (Remarks to the Author):

Mechanisms driving HSV-1 infection of the human brain and associated pathologies are poorly understood. Given the fact that HSV-1 causes encephalitis and the possibility that it may affect neurodegeneration in the CNS, multiple attempts have been made to model the consequences of HSV- 1 infection via human iPSC or ESC 3D cultures often termed as brain organoids or in this case, cerebral organoids. Past studies (summarized in Depla et al., Viruses, 2022; Cairns et al., 2020, Sci Adv) and a 2021 bioRxiv paper by the authors (Rybak-Wolf et al.) elegantly demonstrate the use of such 3D brain

organoid in HSV-1 infection and infection-associated neuronal loss. A major strength of the manuscript is that it is well presented and well written. A few major weaknesses however significantly reduce enthusiasm. A major problem is that the findings are mostly descriptive, lack proper controls and often fail to go too much beyond what is already known in the field. In other words, it lacks depth and the novelty is incremental. Some other issues are listed below.

1. Fig. 1, very little new information is gained from this figure. Similar information can also be found in their 2021 bioRxiv paper.

Our current manuscript originates from that preprint, which was presenting a rather general description of multiple modalities of molecular characterization of HSV-1 infection of brain organoids. In a subsequent effort, we focused on the TNF pathway which was the strongest signature observed from these data, and collected our additional results in the manuscript submitted here. For this reason, Figure 1 of this manuscript presents similar (but more thorough and clearer) results than that in our preprint, as this manuscript is a “version 2” of that preprint. We will replace the old version with the current manuscript version on bioRxiv after re-submission.

1. Fig. 2, a large portion of the results shown here have already been described elsewhere using 3D brain organoids (please see the references above). Calcium imaging data lack evidence whether active viral replication is required or not. Long time ago Cheshenko et al. (JCB, 2003) showed that Ca signaling is needed during viral entry. Use of a replication deficient virus can provide more knowledge on this phenomenon and better explain the observations made in this figure.

The purpose of calcium imaging experiment was only to demonstrate the loss of coordinated neuronal activity upon HSV-1 infection. However, we thank the referee regarding the replication deficient virus comment and we now used such a virus (ICP27 knockout) in the experiments introduced below.

2. Fig. 3, did the authors use sucrose density gradient purified virus here and in the previous figure? If infected cell lysates were used (most common), then many inflammatory/toxic substances present in the lysates can also cause the observations made here. Their control is no viral infection; a true scientific control would be lysates from noninfected cells if the infected cell lysates were used for the infection. Fig 3D, ACV alone control is missing.

We performed the experiments with cell lysates as well as with concentrated purified virus (all experiments with WT and ICP27 mutant in Fig 6 and S6), observing similar effects. Also, the volume of added lysate was never exceeding 1-5% of the total culture medium, and was less than 0.03% for the concentrated virus. However, to better address this

concern, we included an additional non-infected cells lysate as a control for immunostainings/Western analyses (**new Fig 6c,d and Fig S6**). Furthermore, we performed an experiment where we exposed organoids to irradiated (inactive) HSV-1-GFP virus and non- irradiated HSV-1-GFP(active) virus with the same volume of lysate (5%) added and observed an activation of inflammatory responses (p-P65) only with the viable virus. This indicates that small volume of cell lysate added to the organoids cannot drive the observed inflammatory responses (**new Fig S6b**).

3. Figs. 4/5, It is gratifying that TNF signaling and the inability of ACV to reduce its activity are also

recapitulated well in this model. However, this knowledge already exists from previous animal studies

and human clinical observations. The data shown here are descriptive and provide very little new knowledge. Again, ACV alone control is missing, which makes this study less rigorous.

We now added the ACV alone control (**new Fig S6a**) and we thank the referee for pointing out this important control.

However, regarding the other points, to our knowledge, there are no clinical data pointing towards TNF activation after ACV treatment, only clinical observations related to neurological sequelae. Also, in Boivin et al., (2013) only a survival analysis was performed in mice, without any molecular characterization. It is true that it was shown previously that TNF is activated after HSV-1 infection, but there is no study showing that (a) this activation persists after ACV treatment nor (b) that targeting this pathway **prevents** molecular, cellular or tissue-level effects of the infection in a neural tissue. In our paper, we could clearly demonstrate using scRNAseq analysis, immunostaining and western blotting that not only TNF pathway activation remains after ACV treatment, but also that cellular and tissue changes (neuroepithelial integrity, STMN2 expression) associated with viral infection are only partially prevented by current treatments, while complementary treatments that target the inflammatory response better prevent such changes.

4. Fig. 5C/D GFP intensity for the treated organoids never reaches the control levels. Still the data in Fig.6 claims near complete inhibition of the infection. How to truly reconcile the two apparently contrasting results? If GFP intensity has no real meaning, then why to show the data?

In all the data presented in our study, ACV treatment causes a significant downregulation of GFP expression, although not a complete depletion (not even in the Western in Fig. 6a), since ACV only inhibits viral replication but not infection. IF images are usually centered on the external part of the organoids, which is more accessible and more infected (more GFP detected), while Westerns are performed on lysates from whole organoids, where GFP expression is averaged over the entire organoids tissue. To further address this point:

- *We now provide immunofluorescence quantifications in* ***new Fig. S5h*** *for two MOIs; in these two cases, as we would expect, the viral GFP signal is differently impacted by the treatment: with viral replication being controlled much better at lower MOI, compared to higher MOI;*
- *In Fig. 6a, we show Western blots from experiments performed with a similar MOI than that of the “low MOI” treatment in Fig. S5G;*
- *To quantify whether the Western and Immunostainings are in good agreement (and therefore that the GFP signal is indeed meaningful), we show below a scatterplot of the average GFP intensity from the two readouts (Western blots with low MOI and immunofluorescence with low and high MOI) in each condition: the low MOI IF GFP signal is indeed very well correlated to the Western GFP signal (Pearson = 0.979, green dots).*

Furthermore, we have now measured viral GFP expression on DNA, RNA and protein level in organoids from the same batch and could show that there is a good correlation between GFP DNA,

mRNA and protein, therefore we can assume that GFP protein levels as assessed by Western or IF is a good proxy for viral load. We added these data to a supplementary figure in our manuscript (**new Fig S5b and S5h**). Furthermore, we included also the visium spatial transcriptomic data showing the GFP RNA expression through the organoids (**new Fig S2a**).

How deep into the tissue the drugs can penetrate? What would be the mechanism?

We have added sections of organoids infected with the GFP virus that show clearly that upon ACV treatment, no GFP signal is seen in the interior of the organoid, which suggests that we have complete drug penetration, and therefore also repressed viral replication in the interior of the organoid (**new Fig. S3a and S5a**)**.** The mechanisms of acyclovir penetration are not well studied, especially in 3D tissue culture. There are only human skin penetration studies using ACV creams (Nallagundla S. et al., 2014).

HSV-1 GFP	MERGE

DAPI HSV-1

DAPI HSV-1

HSV-1 3dpi

Since they are basically reproducing the prior knowledge from other model systems, the authors should deliver the neurovirulent Y34.5 protein of HSV-1 to demonstrate a viral determinant of the cytopathic/inflammatory effects observed in their cerebral organoids. This way the study will demonstrate a molecular mechanism by which HSV-1 causes damage in the organoids and be less descriptive. Alternatively, the non-neurovirulent HSV-1 Y34.5 deletion virus should be compared with the wildtype virus.

HSV-1 3dpi+ACV

The main claim of our paper is that the damage to the organoids is to a substantial degree mediated by the inflammatory response caused by the virus. In order to further substantiate this claim, we have now added two new experiments. First, we tested the reaction of organoids to filtered supernatant from infected organoids, i.e. we transfer inflammatory mediators such as cytokines, but not the virus itself.

We have indeed seen that this induces substantial significant effects on p-65 phosphorylation (**new Fig**. **6e-h**). Due to the short time frame allowed for the revision, we were not able to obtain and perform experiments with an ICP34.5 knockout virus. However, we use a replication-deficient ICP27 knockout virus (along with the KOS strain wt virus, since the ICP27 knockout is done in the KOS background). As expected, the ICP27 knockout virus can only be seen in the outer cell layer of the organoids, since it cannot replicate and invade the inner parts of the organoids. Still, we observed strong NF-kB activation in the ∆ICP27 HSV-1 infected organoids, as well as substantial structural damage in the vicinity of the infected cell layer (**new Fig. 6 c, d** and **S6c-e**). This again argues that non-cell autonomous effects cause organoid tissue damage.

| **Message:** | Our ref: NMICROBIOL-22071658D  15th March 2023  Dear Nikolaus and Agnieszka,  Thank you for submitting your revised manuscript "Modeling herpes simplex virus 1 infection in cerebral organoids reveals new potential therapeutic approaches for viral encephalitis" (NMICROBIOL-22071658D). It has now been seen by the original referees and their comments are below and thank you for sending a point-by-point response to the comments of reviewer #4. The reviewers find that the paper has improved in revision, and therefore we'll be happy in principle to publish it in Nature Microbiology, pending minor revisions to comply with our editorial and formatting guidelines.  - Please also discuss your choice of 60 days old brain organoids (similar to the response to the points of reviewer #4) and provide the references accordingly. - Please email us a copy of the file in an editable format (Microsoft Word or LaTex)-- we can not proceed with PDFs at this stage.  We will then perform detailed checks on your paper and will send you a checklist detailing our editorial and formatting requirements in several weeks. Please do not upload the final materials and make any revisions until you receive this additional information from us.  Thank you again for your interest in Nature Microbiology Please do not hesitate to contact me if you have any questions.  Sincerely,   [redacted]    Reviewer #2 (Remarks to the Author):  In the present revised version Rybak-Wolf et al. have responded very extensively to all critical points and further conducted multiple appropriate experiments supporting the results and the overall outcome of the study. Based on that I recommend acceptance of the manuscript for publication.   Reviewer #3 (Remarks to the Author):  Addition of new experiments have helped answer many of my original concerns. Novelty remains an issue, but new data helps to circumvent the problem somewhat.   Reviewer #4 (Remarks to the Author):  The manuscript titled “Modeling herpes simplex virus 1 infection in cerebral organoids reveals new potential therapeutic approaches for viral encephalitis” is focused on modeling herpes simplex encephalitis (HSE) using human iPSC-derived cerebral organoids. HSE is caused by herpes simplex viruses (HSV). Because HSV-1 is a human-specific virus, the human iPSC-based organoids provide a great cellular platform for investigating HSV-1-inducd pathology. The authors have addressed multiple concerns raised by the reviewers in the revised manuscript. The revised manuscript has improved over the previous version. However, the major concerns of the previous reviewers, including the concern of the novelty and the lack of depth remain to be addressed. Additional comments: 1. Single cell RNA-seq was performed from two lines. Increasing the sample size to n=3 by including 3 lines will enhance the rigor. 2. The rationale to select a pathway that is consistently upregulated in all analyzed clusters for further study is not clear. Cell-type-specific pathways can play very important roles in the pathogenesis of various diseases. 3. Because glial cells are important for neuroinflammation and GFAP+ glial cells just start to emerge in the organoids around day 60 (Fig. 1c and S1b, d), using day 60 organoids for sc-RNA-seq and infection experiments seem not ideal. Using later stage organoids that have more abundant astrocytes and more mature astrocytes may be more suitable for studying inflammation. Adding microglia, another glial cell type that is important for neuroinflammation will further strengthen the study. 4. At the end of the result section, it was indicated that “the p65 phosphorylation in these organoids was significantly elevated compared to mock medium exposed organoids, suggesting that infected organoids release signaling molecules capable of NF-kB pathways activation independently from viral infection”. It will be interesting to know what signaling molecules capable of NF-kB pathways activation were released from the infected organoids independently from viral infection. |
| --- | --- |

Final Decision Letter

| **Message:** | Our ref: NMICROBIOL-22071658D  15th March 2023  Dear Nikolaus and Agnieszka,  Thank you for submitting your revised manuscript "Modeling herpes simplex virus 1 infection in cerebral organoids reveals new potential therapeutic approaches for viral encephalitis" (NMICROBIOL-22071658D). It has now been seen by the original referees and their comments are below and thank you for sending a point-by-point response to the comments of reviewer #4. The reviewers find that the paper has improved in revision, and therefore we'll be happy in principle to publish it in Nature Microbiology, pending minor revisions to comply with our editorial and formatting guidelines.  - Please also discuss your choice of 60 days old brain organoids (similar to the response to the points of reviewer #4) and provide the references accordingly. - Please email us a copy of the file in an editable format (Microsoft Word or LaTex)-- we can not proceed with PDFs at this stage.  We will then perform detailed checks on your paper and will send you a checklist detailing our editorial and formatting requirements in several weeks. Please do not upload the final materials and make any revisions until you receive this additional information from us.  Thank you again for your interest in Nature Microbiology Please do not hesitate to contact me if you have any questions.  Sincerely,  [redacted]    Reviewer #2 (Remarks to the Author):  In the present revised version Rybak-Wolf et al. have responded very extensively to all critical points and further conducted multiple appropriate experiments supporting the results and the overall outcome of the study. Based on that I recommend acceptance of the manuscript for publication.   Reviewer #3 (Remarks to the Author):  Addition of new experiments have helped answer many of my original concerns. Novelty remains an issue, but new data helps to circumvent the problem somewhat.   Reviewer #4 (Remarks to the Author):  The manuscript titled “Modeling herpes simplex virus 1 infection in cerebral organoids reveals new potential therapeutic approaches for viral encephalitis” is focused on modeling herpes simplex encephalitis (HSE) using human iPSC-derived cerebral organoids. HSE is caused by herpes simplex viruses (HSV). Because HSV-1 is a human-specific virus, the human iPSC-based organoids provide a great cellular platform for investigating HSV-1-inducd pathology. The authors have addressed multiple concerns raised by the reviewers in the revised manuscript. The revised manuscript has improved over the previous version. However, the major concerns of the previous reviewers, including the concern of the novelty and the lack of depth remain to be addressed. Additional comments: 1. Single cell RNA-seq was performed from two lines. Increasing the sample size to n=3 by including 3 lines will enhance the rigor. 2. The rationale to select a pathway that is consistently upregulated in all analyzed clusters for further study is not clear. Cell-type-specific pathways can play very important roles in the pathogenesis of various diseases. 3. Because glial cells are important for neuroinflammation and GFAP+ glial cells just start to emerge in the organoids around day 60 (Fig. 1c and S1b, d), using day 60 organoids for sc-RNA-seq and infection experiments seem not ideal. Using later stage organoids that have more abundant astrocytes and more mature astrocytes may be more suitable for studying inflammation. Adding microglia, another glial cell type that is important for neuroinflammation will further strengthen the study. 4. At the end of the result section, it was indicated that “the p65 phosphorylation in these organoids was significantly elevated compared to mock medium exposed organoids, suggesting that infected organoids release signaling molecules capable of NF-kB pathways activation independently from viral infection”. It will be interesting to know what signaling molecules capable of NF-kB pathways activation were released from the infected organoids independently from viral infection. |
| --- | --- |
